# Controlling the Photophysical Properties of a Series of Isostructural d^6^ Complexes Based on Cr^0^, Mn^I^, and Fe^II^

**DOI:** 10.1021/jacs.3c11580

**Published:** 2024-02-09

**Authors:** Christina Wegeberg, Daniel Häussinger, Stephan Kupfer, Oliver S. Wenger

**Affiliations:** † Department of Chemistry, University of Basel, St. Johanns-Ring 19, 4056 Basel, Switzerland; ‡ Institute of Physical Chemistry, Friedrich Schiller University Jena, Helmholtzweg 4, 07743 Jena, Germany

## Abstract

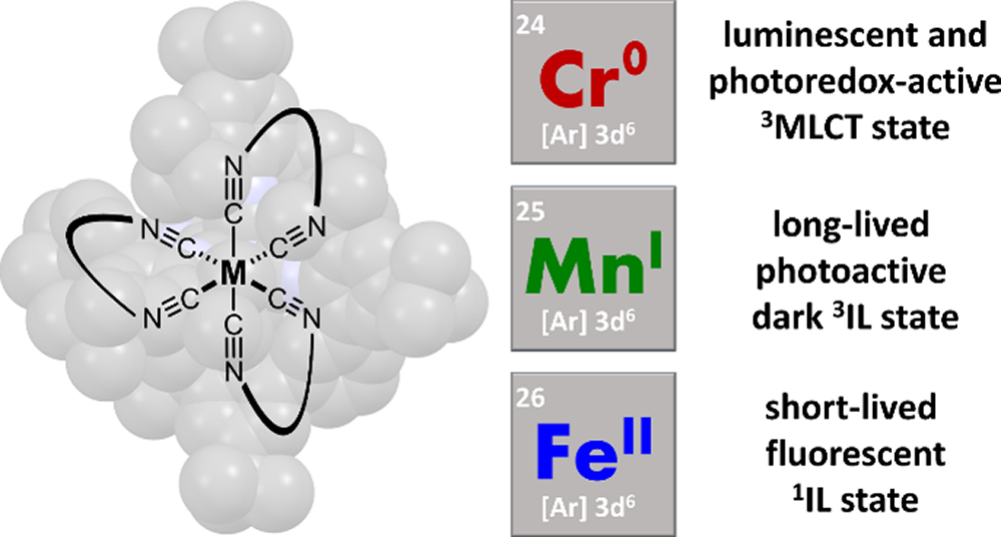

Development of first-row transition metal complexes with similar luminescence and photoredox properties as widely used Ru^II^ polypyridines is attractive because metals from the first transition series are comparatively abundant and inexpensive. The weaker ligand field experienced by the valence d-electrons of first-row transition metals challenges the installation of the same types of metal-to-ligand charge transfer (MLCT) excited states as in precious metal complexes, due to rapid population of energetically lower-lying metal-centered (MC) states. In a family of isostructural tris(diisocyanide) complexes of the 3d^6^ metals Cr^0^, Mn^I^, and Fe^II^, the increasing effective nuclear charge and ligand field strength allow us to control the energetic order between the ^3^MLCT and ^3^MC states, whereas pyrene decoration of the isocyanide ligand framework provides control over intraligand (IL_Pyr_) states. The chromium(0) complex shows red ^3^MLCT phosphorescence because all other excited states are higher in energy. In the manganese(I) complex, a microsecond-lived dark ^3^IL_Pyr_ state, reminiscent of the types of electronic states encountered in many polyaromatic hydrocarbon compounds, is the lowest and becomes photoactive. In the iron(II) complex, the lowest MLCT state has shifted to so much higher energy that ^1^IL_Pyr_ fluorescence occurs, in parallel to other excited-state deactivation pathways. Our combined synthetic-spectroscopic-theoretical study provides unprecedented insights into how effective nuclear charge, ligand field strength, and ligand π-conjugation affect the energetic order between MLCT and ligand-based excited states, and under what circumstances these individual states become luminescent and exploitable in photochemistry. Such insights are the key to further developments of luminescent and photoredox-active first-row transition metal complexes.

## Introduction

**Figure 1:**
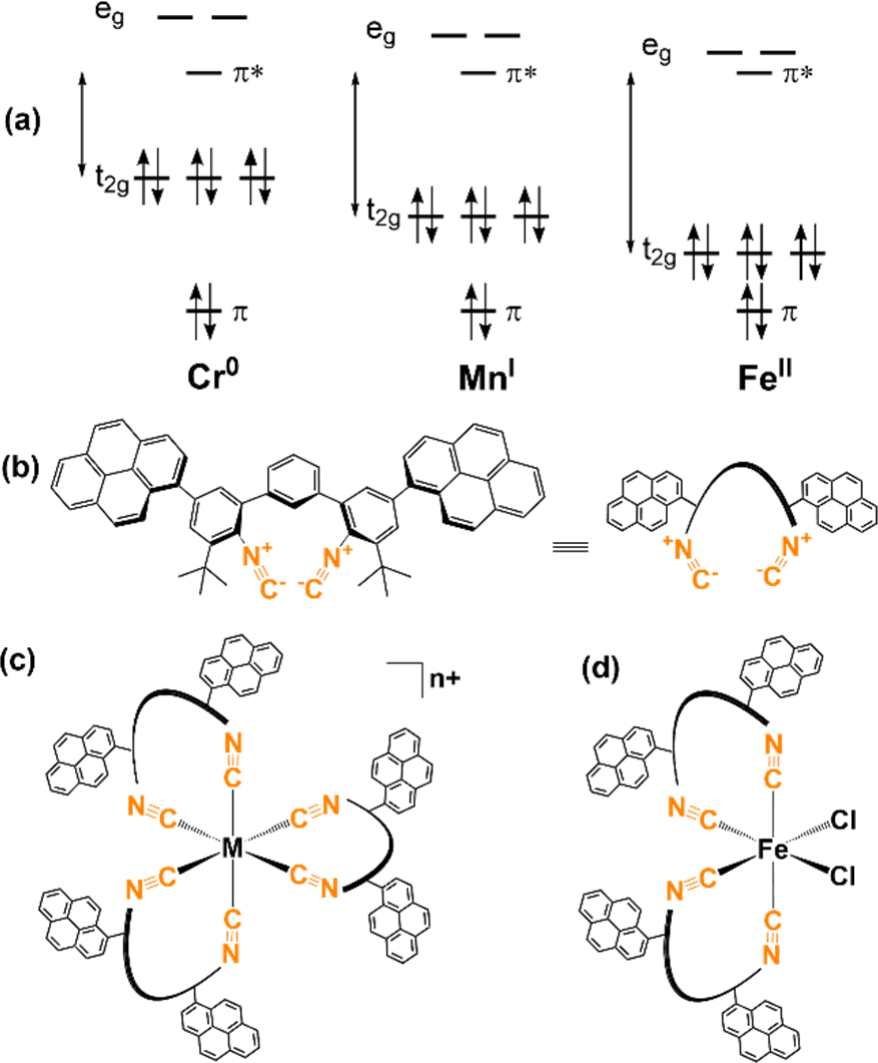
(a) Anticipated trends for frontier orbital energy changes in low-spin 3d^6^ complexes as a function of metal oxidation state based on simple ligand field theory arguments. The emphasis here is on the d-orbital energy trends, whereas the influence of increasing Lewis acidity on ligand-based π and π* orbitals is neglected in this picture. The vertical double arrows mark the energy difference between metal-based t_2g_ and ligand-based π* orbitals, relevant for the lowest-energetic MLCT excited state. (b) Molecular structure of the pyrene-decorated diisocyanide ligand L^Pyr^. (c) Generic molecular structure of the homoleptic complexes [Cr] (M = Cr^0^, *n* = 0), [Mn]^+^ (M = Mn^I^, *n* = 1), and [Fe]^2+^ (M = Fe^II^, *n* = 2). (d) Molecular structure of the [FeCl_2_] reference complex used for structural analysis.

Large interest has emerged in tuning the ligand design of 3d^6^ metal complexes with the goal of obtaining emissive metal-to-ligand charge transfer (MLCT) excited states, analogous to those in ruthenium(II) polypyridine (4d^6^) and cyclometalated iridium(III) (5d^6^) complexes, useful in applications such as dye-sensitized solar cells^1,2^ and photoredox^3−5^ and energy transfer catalysis.^6^ In octahedral d^6^ complexes, where the lowest excited state is of MLCT character, three degenerate metal-based t_2g_ orbitals are each occupied with one electron pair, and empty ligand-based π* orbitals are energetically below the two degenerate metal-based e_g_ orbitals (Figure 1a). The fundamental key challenge in installing photoactive MLCT states for complexes based on 3d^6^ metals is that the ligand field experienced by the first-row transition metals is much weaker relative to that of second- and third-row transition metals in a given coordination environment.^7,8^ As a consequence, the energy order between unoccupied ligand π* and metal e_g_ orbitals can invert, and metal-centered (MC) excited states can start to interfere. MC states typically play a detrimental role in the unwanted deactivation of the MLCT states as soon as the MC states become of comparable energies to the lowest MLCT states,^9−12^ which can lead to nonemissive compounds with very short-lived MLCT states that are not amenable to applications in lighting or photocatalysis.^13^

Iron is the most abundant transition metal in Earth’s crust, and therefore, the development of photoactive first-row transition metal complexes has for decades focused on iron(II) complexes with the same low-spin d^6^ valence electron configuration as the above-mentioned well-known ruthenium(II) and iridium(III) compounds.^14−19^ Some of the most successful approaches have exploited iron(II) complexes with strongly σ-donating ligands such as carbene or cyclometalating ligands, which has allowed for extension of MLCT lifetimes from around 80 fs in the prototypical [Fe(bpy)_3_]^2+^ (bpy = 2,2′-bipyridine) to the nanosecond regime in solution at room temperature.^20−25^ Recently, ligands with either π-donating^26,27^ or π-accepting^26−28^ properties have also shown useful for obtaining long-lived charge-transfer excited states.

Despite the obvious goal of obtaining photoactive complexes based on abundant iron(II),^29^ it is appealing to furthermore expand the search to chromium(0), manganese(I), and cobalt(III) as these ions likewise possess the privileged low-spin d^6^ valence electron configuration, and the respective metals are all far more abundant than the above-mentioned noble elements.^13^ The effective nuclear charge will increase along the series from chromium(0) to cobalt(III) as the oxidation state of the metal increases, resulting in contraction of the d orbitals and entailing an overall energetic stabilization of both the t_2g_ and e_g_ orbitals (Figure 1a). Commonly, the ligand field strength parameter (10 Dq) increases with increasing metal oxidation state for a given valence electron configuration, for instance from 10 Dq = 21,000 cm^–1^ in [Fe(bpy)_3_]^2+^ to 10 Dq = 24,480 cm^–1^ for [Co(bpy)_3_]^3+^.^30^ The absolute energies of the t_2g_ and e_g_ orbitals will be governed by the individual metal–ligand combination, whereby the effective nuclear charge of the metal is expected to play a decisive role. The molecular orbital energy diagrams presented in Figure 1a provide a simplistic picture illustrating the approximately expected outcome when changing the metal from chromium(0) to manganese(I) and iron(II) in isostructural octahedral complexes based on simple ligand field theory arguments. A more elaborate picture to assess the complex interplay between σ- and π-interactions among the series of metal centers and the ligand framework, and the resulting implications with respect to ground and excited state properties, will be provided by *state-of-the-art* quantum chemical simulations further below.^31,32^

Recent work compared isostructural low-spin d^6^ complexes of iron(II) and cobalt(III) in polypyridine coordination environments, yielding important insights regarding the MC excited states within the low-spin d^6^ valence electron configuration.^30,33−35^ Here, in the present study, the focus is on MLCT excited states and on lower-valent d^6^ complexes in isocyanide ligand fields, which are substantially stronger than the ligand fields provided by polypyridines. Following early studies of group 6 metal complexes with isocyanide ligands^36,37^ and more recent reports on luminescent tungsten(0) complexes with monodentate isocyanide,^38−40^ our group discovered that chelating diisocyanide ligands facilitate access to luminescent chromium(0),^41^ molybdenum(0)^42−46^ and manganese(I)^47^ complexes. An initially investigated chromium(0) isocyanide complex absorbed strongly in the red part of the visible spectrum, whereas a later explored manganese(I) complex absorbed primarily UV light, tailing into the blue part of the visible spectrum.^41,47,48^ This drastic difference in UV–vis absorption properties seems largely attributable to the energetic stabilization of the t_2g_ orbitals in manganese(I) relative to chromium(0) (Figure 1a),^49^ but the ligands were not identical in the two previous studies and the influence of changes in ligand-based π* orbital energies could not be elucidated. In other studies, we found that attachment of π-extended pyrene substituents in the bidentate isocyanide ligand scaffold resulted in massive improvements of the photophysical properties of chromium(0) complexes, due to the delocalization of the excited electron in the photoactive MLCT state.^50,51^ The homoleptic chromium(0) complex [Cr] (Figure 1c) displayed a ^3^MLCT lifetime (τ_MLCT_) of 47 ns and a luminescence quantum yield (φ_MLCT_) of 1.04% in deaerated cyclohexane at 20 °C, competitive with benchmark osmium(II) polypyridine compounds.^51^ These figures of merit for [Cr] are unmatched by any 3d^6^ compound to date and have enabled photocatalytic behavior in both photoredox catalysis^51^ and photochemical upconversion.^52^

The lowest ^1^MLCT absorption band of [Cr] is red-shifted by ∼3700 cm^–1^ relative to a structurally similar Cr^0^ tris(diisocyanide) complex without the pyrene decoration primarily due to stabilization of the π*-orbitals.^41^ Such energetically stabilized π*-orbitals are favorable to lower the energies of MLCT excited states relative to MC excited states, and could potentially give access to long-lived and emissive MLCT states in manganese(I) and iron(II) tris(diisocyanide) complexes. Against this background, it seemed interesting to explore the electronic structures and detailed photophysical properties of the previously unknown homoleptic [Mn]PF_6_ and [Fe](PF_6_)_2_ compounds (Figure 1c), in addition to the previously reported [Cr] complex. The heteroleptic iron(II) complex [FeCl_2_] (Figure 1d) serves as a structural reference compound. By mapping the photophysical properties of the isoelectronic and isostructural complexes [Cr], [Mn]^+^, and [Fe]^2+^, our study provides new insights on how to control the order of MLCT, intraligand (IL) and MC states in 3d^6^ compounds. Our work furthermore showcases how completely different photoactivity can be switched on by combining the same set of ligands with different 3d^6^ metals.

## Results and Discussion

### Synthesis and Ground State Characterization

The 2-fold pyrene-decorated ligand L^Pyr^ (Figure 1b) and the [Cr] complex (Figure 1c) were synthesized according to our previously published procedure.^51^ The new [Mn]PF_6_, [Fe](PF_6_)_2_, and [FeCl_2_] compounds (Figure 1c,d) were prepared from L^Pyr^ and the commercially available metal precursors Mn(CO)_5_Br, Fe(OTf)_2_ and FeCl_2_, respectively (Scheme S1). The syntheses of the two iron(II) complexes were carried out in THF at room temperature. [FeCl_2_] was readily obtained in 35% yield by precipitation from the reaction mixture with *n*-pentane. Following an anion exchange from triflate to hexafluorophosphate and precipitation with diethyl ether, [Fe](PF_6_)_2_ was obtained in 46% yield. The synthesis of [Mn]PF_6_ required harsher reaction conditions in order to avoid contamination with manganese carbonyl byproducts, and therefore the complexation step was performed in refluxing toluene. Under these reaction conditions, [Mn]Br precipitated from the reaction mixture, and following anion exchange, [Mn]PF_6_ was obtained in 18% yield.

High-resolution mass spectrometry exhibited the expected peaks at *m*/*z* 2432.9863 and 1216.9924 assigned to [Mn]^+^ and [Fe]^2+^, respectively. The dominant peak for [FeCl_2_] was detected at *m*/*z* 1676.6044, corresponding to [FeCl_2_] after loss of one chloride ligand to generate a positively charged ion.

**Figure 2:**
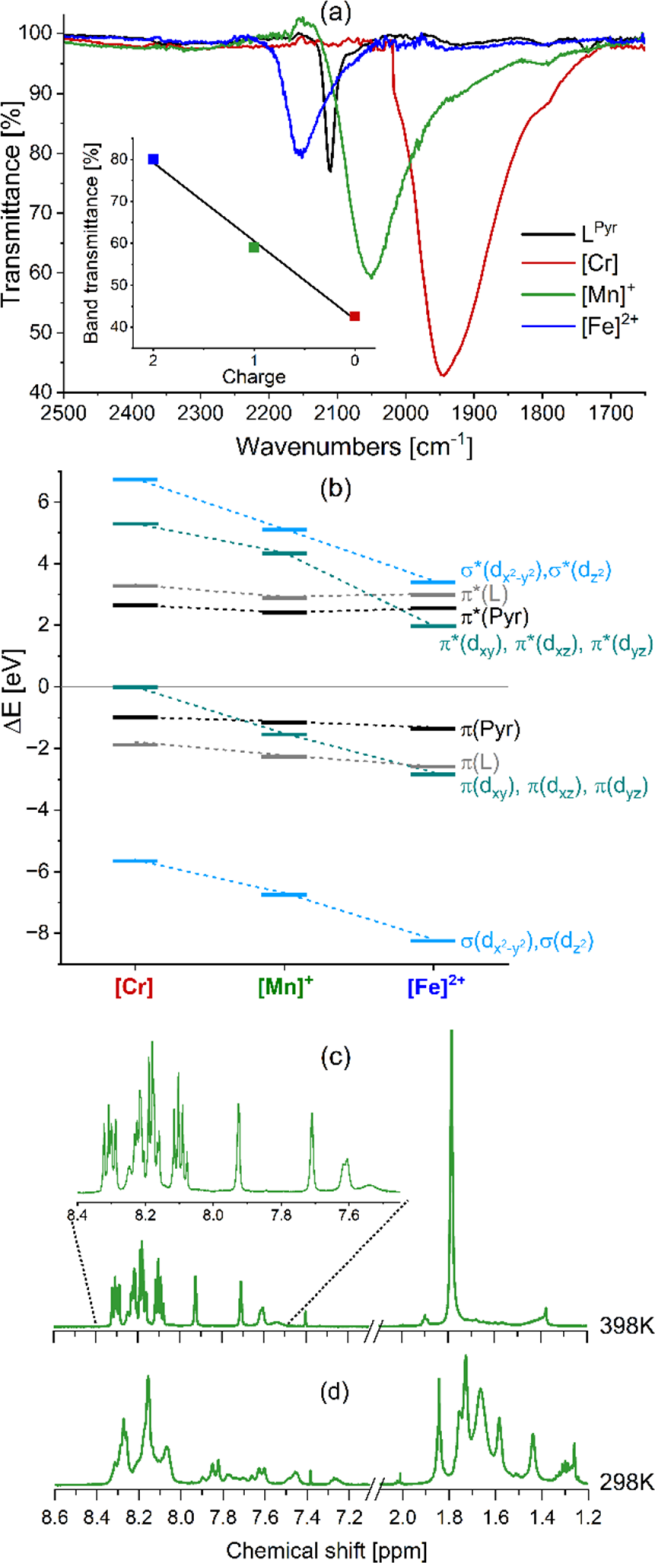
(a) Solid-state FTIR spectra of L^Pyr^, [Cr], [Mn]PF_6_, and [Fe](PF_6_)_2_ in the C≡N stretching frequency region. (b) Frontier orbital energy diagram for [Cr], [Mn]^+^ and [Fe]^2+^ obtained at the B3LYP/def2-SVP level of theory (in THF); *tert*-butyl groups were simplified to methyl groups (see computational details in SI, high-resolution orbital plots are available for download in ref (57)). ^1^H NMR spectra of [Mn]^+^ at 398 K (c) and 298 K (d) in TCE-*d*_2_ (600 MHz) recorded on the same sample. Only the resonance of the residual solvent peak is found between 2.0 and 7.1 ppm (at 6.00 ppm); hence, this spectral region is omitted for clarity. At 398 K (c), the resonance at 1.78 ppm is due to the *tert*-butyl groups, whereas the broader resonances at 7.53 and 7.60 ppm originate from protons on the central *m*-terphenyl ring. For assignment of the individual ^1^H NMR resonances of [Mn]^+^, see Figure S8.

The C≡N stretching (ν_C≡N_) vibrations in the infrared (IR) spectra of isocyanide complexes are instrumental in determining the extent of π-backbonding from the metal to the π* antibonding orbitals of the isocyanide moiety.^53,54^ The C≡N stretching band of [Mn]PF_6_ is found at 2052 cm^–1^ (Figure 2a and Table 1), which is in between the C≡N stretching bands previously reported for L^Pyr^ and [Cr] at 2111 and 1945 cm^–1^, respectively.^51^ The C≡N stretching bands of [FeCl_2_] and [Fe](PF_6_)_2_ are found at higher frequencies than L^Pyr^, namely at 2125 and 2159 cm^–1^, respectively. Similar C≡N stretching frequencies have previously been reported for related manganese(I) and iron(II) isocyanide complexes.^47,49,55,56^ This change in the C≡N stretching frequencies is well reflected by the quantum chemical simulations performed for [Cr], [Mn]^+^, and [Fe]^2+^. Density functional theory (DFT) predicts the three strongly IR-active and almost degenerate C≡N stretching modes at 1942, 2050, and 2147 cm^–1^ for [Cr], [Mn]^+^ and [Fe]^2+^ (Table S15), respectively. Based on the computational modeling, this trend originates from the pronounced decrease in energy of the involved π(d) orbitals (π(d_*xy*_), π(d_*xz*_), π(d_*yz*_) in Figure 2b; equivalent to t_2g_ in Figure 1b) by 1.5 eV for [Mn]^+^ and 2.8 eV for [Fe]^2+^ with respect to [Cr]. All four complexes show much broader C≡N stretching bands than what is observed for L^Pyr^. This feature is presumably due to a somewhat distorted octahedral coordination geometry of the bulky ligands around the 3d^6^ metals, resulting in slightly different metal–ligand bond lengths and angles and, thus, to the three weakly dipole-allowed vibrational normal modes predicted by DFT at slightly higher wavenumbers than the most intense C≡N stretching mode (Table S15). Focusing on the isostructural series [Cr], [Mn]PF_6_, and [Fe](PF_6_)_2_, it is evident that the stepwise increase in metal oxidation state weakens the π-backbonding from the metal to the isocyanide ligand, resulting in the gradual increase of the C≡N bond strength. Moreover, going from Cr^0^ to Mn^I^ and Fe^II^, the strength of the metal–carbon σ-bonding increases as a result of the increasing metal Lewis acidity. This behavior is evident based on the respective σ(d) orbitals of the three isoelectronic complexes (Figure 2b), as the energy of two degenerate σ-orbitals, i.e.,  and , is stabilized significantly from −5.6 eV in the case of [Cr] to −6.7 eV for [Mn]^+^ and further to −8.2 eV for [Fe]^2+^. Effectively, this σ-bonding effect moves electron density from the CNR ligand to the metal, and since the relevant electron density originates from a molecular orbital with substantial σ-antibonding CN character, the C≡N bond gets stronger by this effect. Consequently, the higher energy of the C≡N stretching vibrations of [Fe](PF_6_)_2_ compared to the free ligand indicates that σ-bonding effects dominate over π-backbonding effects in the iron(II) compound. As the C≡N bond strengthens from [Cr] to [Mn]PF_6_ and [Fe](PF_6_)_2_, the intensity of the C≡N stretching band decreases in a linear fashion as a function of metal charge (Figure 2a, inset). A similar trend has been observed for other isostructural 3d^6^ tris(diisocyanide) complexes.^49^

**Table 1:** Overview of Key Ground State Parameters for the Isostructural Series [Cr], [Mn]PF_6_, and [Fe](PF_6_)_2_

complex	ν_C≡N_ (cm^–1^)	Δ*E*_t2g_ (eV)	Δ*G*^‡^ (kJ/mol)	*E*_1/2_ of first metal oxidation vs Fc^+^/Fc^0^ (V)^a^	*E*_1/2_ of ligand reduction vs Fc^+^/Fc^0^ (V)^a^	ref
[Cr]	1945	0	55^b^	–0.68	–2.51	(51), this work
[Mn]PF_6_	2052	–1.5	69^c^	0.68	–2.12	this work
[Fe](PF_6_)_2_	2159	–2.8	>83^c^	d	e	this work

a Data obtained in deaerated THF at 20 °C.

b Data obtained in deaerated toluene-d_8_.

c Data obtained in deaerated TCE-*d*_2_.

d No oxidation wave was identified in the electrochemically accessible window of THF.

e Not determined as [Fe](PF_6_)_2_ is unstable at potentials lower than −1.5 V. Δ*E*_t2g_: energy stabilization of the t_2g_ level relative to that of [Cr].

The atomic/ionic radii decrease in the series chromium(0), manganese(I), and iron(II) from 140 to 74 pm.^58,59^ This decrease in the size of the metal species reflects in the dynamic behaviors of [Cr], [Mn]^+^, [Fe]^2+^, and [FeCl_2_] in solution as monitored by ^1^H NMR spectroscopy. The ^1^H and ^13^C NMR resonances of L^Pyr^ have previously been fully assigned in toluene-d_8_,^51^ but for direct comparison with [Mn]^+^, [Fe]^2+^, and [FeCl_2_], we include here an NMR analysis in tetrachloroethane-d_2_ (TCE-*d*_2_) at room temperature, which yielded the complete assignment of all proton and carbon spins including the isocyanide carbon atoms at 172.7 ppm. As expected, the NMR spectra of L^Pyr^ reflect the symmetrical nature of the uncoordinated ligand, and the NOE pattern indicates free rotation of the three rings of the *m*-terphenyl with respect to each other, as well as unhindered rotation of the pyrene moiety. An entirely different picture is, however, valid upon coordination of L^Pyr^ to the 3d^6^ metals: at room temperature, the ^1^H NMR spectra of the homoleptic complexes [Cr], [Mn]^+^ and [Fe]^2+^ display broad, unstructured proton resonances, indicative of steric clashes between the six *tert*-butyl groups that result in hindered rotations around the central C_Aryl_-C_q_ and the C_q_-CH_3_ bonds (Figures S5, 2d and S14, respectively). These rotations and possibly other dynamic processes on the millisecond time scale, e.g., distortions of the *m*-terphenyl plane, effectively lower the D_3_ symmetry of the complexes in solution at 298 K and give rise to many different rotamers in slow exchange with each other on the NMR time scale. Only the heteroleptic [FeCl_2_] complex with the least crowded coordination environment among the investigated complexes has sharp proton resonances at room temperature (Figure S18). [Cr] has previously been fully characterized at 378 K in toluene-d_8_ as apparently D_3_ symmetric,^51^ and in order to resolve the dynamic behavior of the homoleptic series [Cr], [Mn]^+^, and [Fe]^2+^, we likewise performed variable temperature NMR experiments between 298 and 408 K for [Mn]^+^ (Figure S12) and [Fe]^2+^ (Figure S16) in TCE-*d*_2_. As the temperature is increased from 298 to 398 K (Figure 2c,d), the proton resonances of [Mn]^+^ sharpen. At 398 K, fast conformational exchange in the NMR time regime is reached, and a single set of sharp ^1^H NMR resonances is observed. The line widths at 398 K of the proton resonances on the central ring in the *m*-terphenyl at 7.53, 8.24, and 7.60 ppm remain with 32, 11, and 8 Hz, respectively, however, notably broader than for protons on the two outer rings and the pyrene unit (ca. 3 Hz). This finding suggests that the central ring of the *m*-terphenyl backbone still slowly flips back and forth between at least two different orientations relative to the two outermost arylisocyanide units at this high temperature, as discussed previously for related ligands in Mo^0^ complexes.^45^ In contrast, the resonances of the pyrene substituents coalesce already below 338 K, indicating that they are not crucially involved in the observed conformational exchanges. At 298 K, several individual *tert*-butyl resonances are found, but at 398 K only one single resonance at 1.78 ppm is observed. The coalescence pattern of the *tert*-butyl resonances suggests that the underlying dynamic process of [Mn]^+^ is centered on the *tert*-butyl groups. As a first approximation, we analyzed the coalescence of the *tert*-butyl signals as a two-state exchange by the Eyring equation^60,61^ and found an activation barrier (Δ*G*^‡^) of 69 ± 1 kJ/mol to reach fast conformational exchange in [Mn]^+^ (Tables 1 and S1). A similar behavior was observed for [Cr] (Figure S7) and a related chromium(0) complex with *tert*-butyl groups in the *ortho* position to the isocyanide groups.^50,51^ For the isostructural [Cr], the Eyring analysis yielded an activation barrier of 55 ± 1 kJ/mol. The proton on the central *m*-terphenyl ring pointing toward the metal center has a chemical shift of 8.81 ppm in [Cr], and in [Mn]^+^, this proton resonance has shifted upfield to 8.24 ppm. The chemical shift of this proton is 7.82 ppm for the free L^Pyr^ ligand. Evidently, this proton is more shielded in [Mn]^+^ than in [Cr]. As the two complexes are isostructural, this effect must be caused by the higher charge of manganese(I) and the smaller ionic radius relative to the atomic radius of chromium(0).

The metal radius of [Fe]^2+^ is even smaller, and thus, the ligand environment is expected to be even more contracted in this complex than in [Mn]^+^. In accordance with this hypothesis, [Fe]^2+^ showed broad proton NMR spectra over the temperature range from 298 to 408 K without undergoing coalescence at all (Figure S16). Unfortunately, [Fe]^2+^ decomposed after prolonged heating above 378 K in TCE-*d*_2_. A complete assignment of protons and carbons was, therefore, not feasible (Figure S13), but a lower boundary for the activation barrier of 83 ± 1 kJ/mol could be extracted (Table S1). The trend of the activation barrier from 55 kJ/mol for [Cr] to 69 kJ/mol for [Mn]^+^ and larger than 83 kJ/mol for [Fe]^2+^ thus nicely corroborates the influence of the smaller metal radius in this isostructural series.

The cyclic voltammogram (CV) of [Mn]^+^ in THF shows a quasi-reversible wave at 0.68 V vs Fc^+^/Fc^0^ assigned to the Mn^II^/Mn^I^ couple and an irreversible wave at −2.12 V vs Fc^+^/Fc^0^ attributed to a ligand-based reduction (Figure S25). The potential for oxidation of manganese(I) to manganese(II) is in good agreement with previous reports on related manganese(I) hexakis(arylisocyanide) complexes (Table S3).^47,49,55^ The Cr^I^/Cr^0^ redox couple is at −0.68 V vs Fc^+^/Fc^0^ for [Cr],^51^ which means that the potential of the same d^5^/d^6^ couple increases by 1.4 V between [Cr] and [Mn]^+^. Reversible ligand reduction in [Cr] occurs at −2.51 V vs Fc^+^/Fc^0^,^51^ and coincides with the reduction of unsubstituted (free) pyrene found at −2.5 V vs Fc^+^/Fc^0^.^62^ The reduction of the *m*-terphenyl backbones of the diisocyanide ligands is outside the electrochemical window of suitable electrolytes.^41^ Evidently, the 0.39 V shift between the ligand-centered reductions in [Cr] and [Mn]^+^ suggests that the oxidation state of the metal has a sizable influence on the reduction of the ligand. In other words, there seems to be an electrostatic effect on the ligand’s reduction potential, whereby the + I oxidation state of Mn^I^ facilitates ligand reduction with respect to the zerovalent oxidation state of Cr^0^. [Fe]^2+^ decomposes at potentials below −1.5 V vs Fc^+^/Fc^0^, and the Fe^III^/Fe^II^ redox couple was not detectable in the electrochemical solvent window of THF. Assuming that there is a linear correlation between the charge of the metals and the respective d^5^/d^6^ as well as the ligand-centered redox potentials, the redox potentials found for [Cr] and [Mn]^+^ extrapolate to values of 2.1 and −1.7 V vs Fc^+^/Fc^0^, respectively, for [Fe]^2+^, which helps explain why these redox events were not experimentally observable in THF. It was also not possible to determine the Fe^III^/Fe^II^ reduction potential in related tris(diisocyanide) iron(II) complexes.^49^ These experimental findings agree well with the redox properties simply derived from the energies of the associated molecular orbitals (Figure 2b). Based on DFT calculations, the first oxidation of [Cr] is metal-centered and involves the π-backbonding orbitals, while the first reduction is associated with the pyrene moieties. In the case of [Mn]^+^, the respective π-backbonding orbitals are lowered energetically by 1.5 eV with respect to [Cr], which is in good agreement with the experimentally determined oxidation potential lowered by 1.4 V. However, pyrene-based oxidation at a slightly lower energy of ∼1.1 eV cannot be excluded based on this simple molecular orbital scheme approach. In full agreement with the observed shift of ∼0.3 V to more positive potentials for the reduction wave between [Cr] and [Mn]^+^, the simulations predict the pyrene-centered reduction event to be more favorable by ∼0.2 eV for [Mn]^+^ in comparison to [Cr]. Finally, based on our calculations, metal-centered oxidation for [Fe]^2+^ is expected to be 2.8 eV more energy-demanding than for [Cr], supporting the lack of experimental detection of an Fe^II^ to Fe^III^ oxidation event. The calculations further suggest that the decomposition of [Fe]^2+^ could be related to a metal-centered reduction, i.e., the population of the low-lying LUMO(s) of [Fe]^2+^, which have π*(d) character featuring a nodal plane between iron and the coordinating carbon atoms (see orbitals 610–612 in ref (57) for the isoelectronic [Cr]). Thus, such a reduction event weakens the π-backbonding and can lead to the experimentally observed degradation.

**Figure 3:**
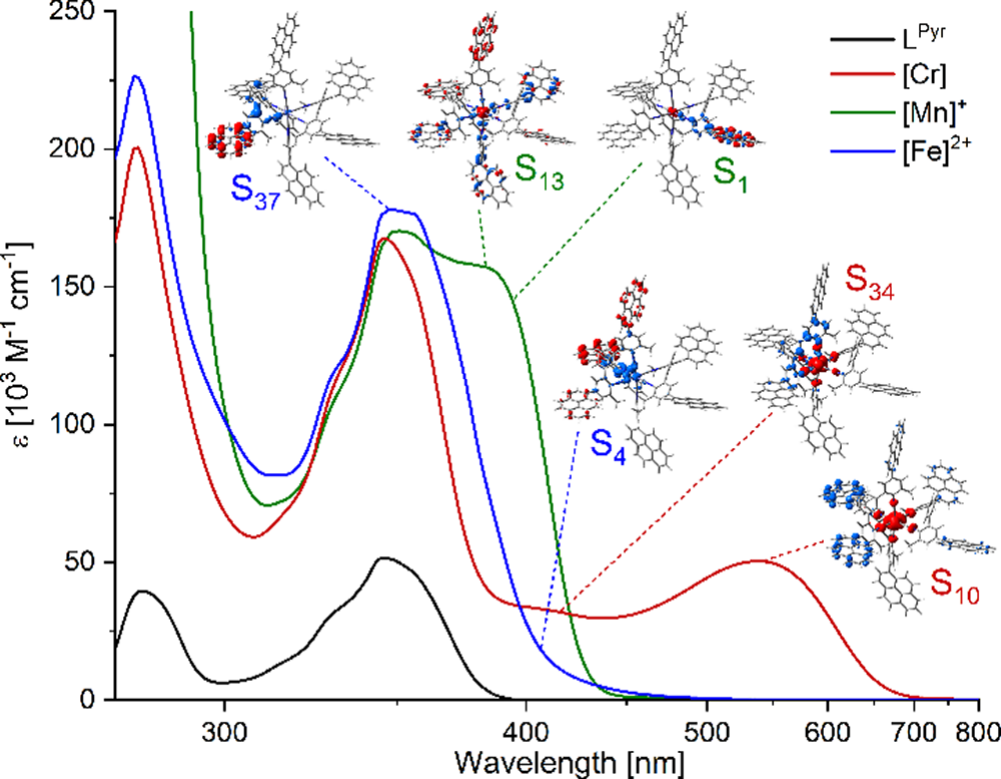
UV–vis absorption spectra of L^Pyr^, [Cr], [Mn]^+^ and [Fe]^2+^ in THF at 20 °C. The wavelength-scale on the horizontal axis is scaled such that the absorption spectra appear correctly on an energy scale. Insets: charge density differences of key singlet–singlet (S_0_ → S_*i*_) excitations within the Franck–Condon geometry (i.e., S_0_ equilibrium) of [Cr] (S_10_, S_34_), [Mn]^+^ (S_1_, S_13_), and [Fe]^2+^ (S_4_, S_37_) as obtained by TDDFT. Charge transfer takes place from red to blue (see Tables S16–S21 for detailed pictures of the electron density differences or high-resolution images in ref (57)).

Dilute solutions of [Mn]^+^ and [Fe]^2+^ in THF are yellow, in stark contrast to the deep purple color of [Cr] caused by its broad MLCT absorption band between 470 and 700 nm (Figure 3). According to the performed time-dependent DFT (TDDFT) simulations (Figure S79, Tables S16 and S19), this broad and intense visible absorption feature of [Cr] stems from a manifold of strongly dipole-allowed ^1^MLCT_Pyr_ transitions from the π(d) orbitals of the chromium(0) atom to the low-lying π_Pyr_^*^ orbitals predicted between 528 and 538 nm (see exemplary S_10_ at 534 nm in Figure 3), while the absorption shoulder at roughly 420 nm is associated with ^1^MLCT_L_ transitions toward the π_L_^*^ orbitals of the *m*-terphenyl part of the coordinating isocyanide framework (S_34_ and S_35_ at 422 and 419 nm, respectively) without any contributions from the pyrene moieties.

The UV–vis absorption spectrum of [Mn]^+^ exhibits a band centered at 388 nm (Figures 3 and S44), which extends into the blue part of the visible spectrum and is not present in the free ligand or any of the other complexes. The molar extinction coefficient of [Mn]^+^ at 410 nm is 75,000 M^–1^ cm^–1^, which is approximately four times larger than for a related tris(diisocyanide) manganese(I) complex without the pyrene decoration,^47^ i.e., the current ligand design has increased the visible-light absorption capacity considerably. TDDFT allows to assign this absorption band at 388 nm to mixed ^1^IL_Pyr_/^1^MLCT_Pyr_ transitions (S_1_ in Figure 3, Tables S17 and S20), which are significantly blue-shifted with respect to ^1^MLCT transitions of the more electron-rich chromium(0) species. The increased energy is predominantly the result of the increased effective nuclear charge felt at the location of the relevant d-orbitals for manganese(I) relative to that of chromium(0), resulting in a significant stabilization of the π(d) orbitals (π(d_*xy*_), π(d_*xz*_), π(d_*yz*_) in Figure 2b; equivalent to t_2g_ in Figure 1a). The assignment of mixed IL/MLCT character of [Mn]^+^ for the lowest-lying transition agrees with related studies on manganese(I) hexakis(arylisocyanide) complexes.^36,47,49,55^ Furthermore, prior computational studies on tungsten(0) and rhenium(I) arylisocyanides have likewise shown that enhanced extinction coefficients can result from the lowest transition having mixed MLCT and π–π* (C≡N–C) character.^40,63^

The UV–vis absorption spectra of [Cr], [Mn]^+^, and [Fe]^2+^ (Figure 3) all display an intense band centered at 350 nm originating primarily from ^1^π–π* transitions involving pyrene moieties, as also seen in the free L^Pyr^ ligand (Table 2). At shorter wavelengths, ^1^π–π* transitions from the *m*-terphenyl backbone of the isocyanide ligands appear.^41^ The molar extinction coefficients of the ^1^IL_Pyr_ absorption at 350 nm are three times larger in the homoleptic complexes relative to L^Pyr^, which is in agreement with the stoichiometry of three ligands per metal. The UV–vis absorption spectra of L^Pyr^ and [Fe]^2+^ are very similar to each other, and no unique bands related to [Fe]^2+^ are evident; however, the ^1^IL_Pyr_ absorbance band at 350 nm is slightly broadened, also relative to [Cr] (compare the blue and red traces in Figure 3). Subtraction of the normalized absorption spectrum of L^Pyr^ from that of [Fe]^2+^ reveals an overlapping band with a maximum at 375 nm (Figure S60), which according to quantum chemical simulations primarily originate from intraligand charge transfer (ILCT) states with some minor admixed metal character (Tables S18 and S21). The theoretical results furthermore suggest that not only IL_Pyr_ states but a broad variety of electronic transitions are associated with the absorption feature at 350 nm—stemming from further MLCT transitions in the case of [Cr], mixed IL/MLCT excitations in [Mn]^+^ to ILCT states with even slight contributions of ligand-to-metal charge transfer (LMCT) character for [Fe]^2+^. Notably, MLCT transitions are not observed at all within the simulated 300 lowest-lying singlet excited states for [Fe]^2+^. This implies that the MLCT energy of [Fe]^2+^ is shifted to much higher energies with respect to [Mn]^+^ and [Cr], as supported by the lack of a metal-centered oxidation event of [Fe]^2+^ by electrochemistry. The significant differences in MLCT energies for [Cr], [Mn]^+^ and [Fe]^2+^ overall reflect the increasing stabilization of the t_2g_ orbitals along the series chromium(0) < manganese(I) < iron(II) (Figure 2b). As a result, the electronic character of the lowest-lying transitions changes significantly along the series [Cr], [Mn]^+^ and [Fe]^2+^ as visualized by the charge density differences of key transitions of the three complexes in Figure 3.

**Table 2:** Overview of Selected Photophysical Parameters of the Pyrene-Decorated Ligand L^Pyr^ and the Isostructural Series [Cr], [Mn]PF_6_, and [Fe](PF_6_)_2_^a^

compound	λ_abs,1ILPyr_ (nm)	λ_em,1ILPyr_ (nm)	τ_1ILPyr_ (ns)	ϕ_1ILPyr_ (%)	λ_abs,CT_ (nm)^b^	λ_em,^3^MLCT_ (nm)	τ_^3^MLCT_	ϕ_^3^MLCT_ (%)	λ_em,max_ at 77 K (nm)^c^	ref
L^Pyr^	345	410	4.2	86	N/A	N/A	N/A	N/A	385	this work
[Cr]	350	N/A	N/A	N/A	540	840	1.1 ns	0.01	727	(51), this work
[Mn]PF_6_	350	430	3.1	2.4	388	N/A	14 ps	dark state	435; 610	this work
[Fe](PF_6_)_2_	350	415	3.8	14	375	N/A	N/A	dark state	435	this work

a Solution state data were recorded in THF at 20 °C using deaerated solvents for [Cr] and [Mn]^+^ and aerated solvents for L^Pyr^ and [Fe]^2+^.

b Absorption band maxima refers to the lowest-lying charge transfer state, i.e., MLCT for [Cr], IL/MLCT for [Mn]^+^ and ILCT for [Fe]^2+^.

c Data obtained in deaerated 2-methyl-THF.

### Excited State Dynamics

**Figure 4:**
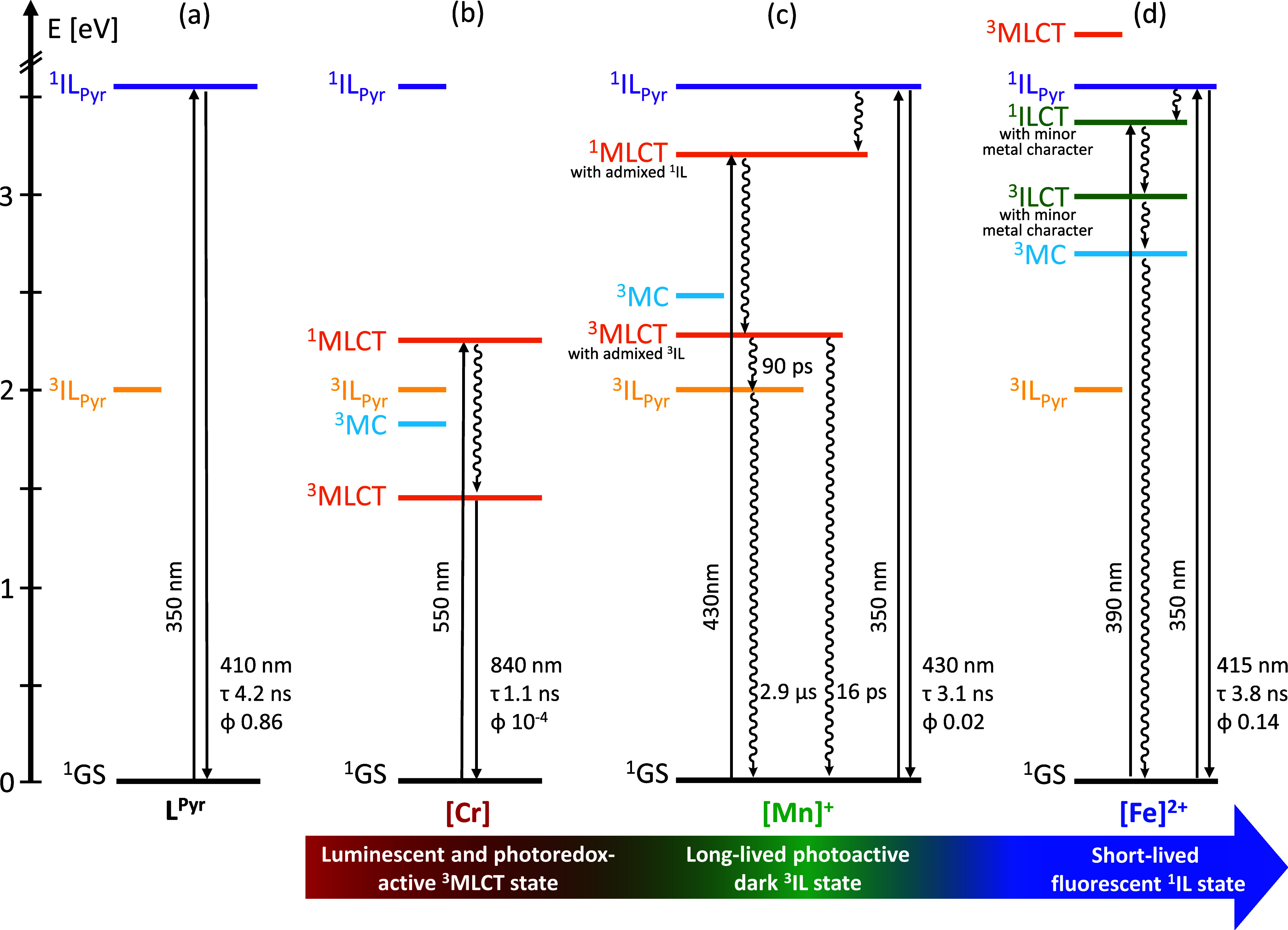
Simplified Jablonski diagrams depicting the photophysical processes of (a) L^Pyr^, (b) [Cr], (c) [Mn]^+^ and (d) [Fe]^2+^ in THF at 20 °C. The photophysical parameters are based on experiments performed in deaerated THF for [Cr] and [Mn]^+^ and aerated THF for [Fe]^2+^ and L^Pyr^. The absolute energies of the ^3^MC states are not experimentally accessible; hence, the ^3^MC states were placed relative to the ^1/3^MLCT manifold to account for the observed deactivation processes. Solid and wavy vertical lines indicate radiative and nonradiative transitions, respectively. Short horizontal lines, e.g., ^3^MC for [Mn]^+^, indicate that there is no evidence for significant involvement of that electronic state in the overall excited-state deactivation processes. ^5^MC states are known to play important roles in iron(II) complexes but have been omitted in the figure here to simplify the overall picture.

The excited state dynamics of [Mn]^+^ and [Fe]^2+^ were investigated in THF at 20 °C. The overlap between the absorption band at 350 nm with predominantly IL_Pyr_ character mixed with charge transfer transitions at slightly lower energies in these two complexes required photophysical characterization of the free L^Pyr^ ligand, to identify possible ligand-based contributions to the excited-state evolution in the metal complexes. The excited state dynamics of [Cr] on the nanosecond time scale have previously been investigated with transient absorption spectroscopy in cyclohexane and toluene.^51^ In the current study, we extend that analysis to include an investigation of the early state dynamics in THF, a solvent in which all of the three investigated complexes solubilize, thus allowing for direct comparison of deactivation pathways and relative energies of MLCT, IL, ILCT, and MC excited states (Figure 4).

#### Free L^Pyr^ Ligand

**Figure 5:**
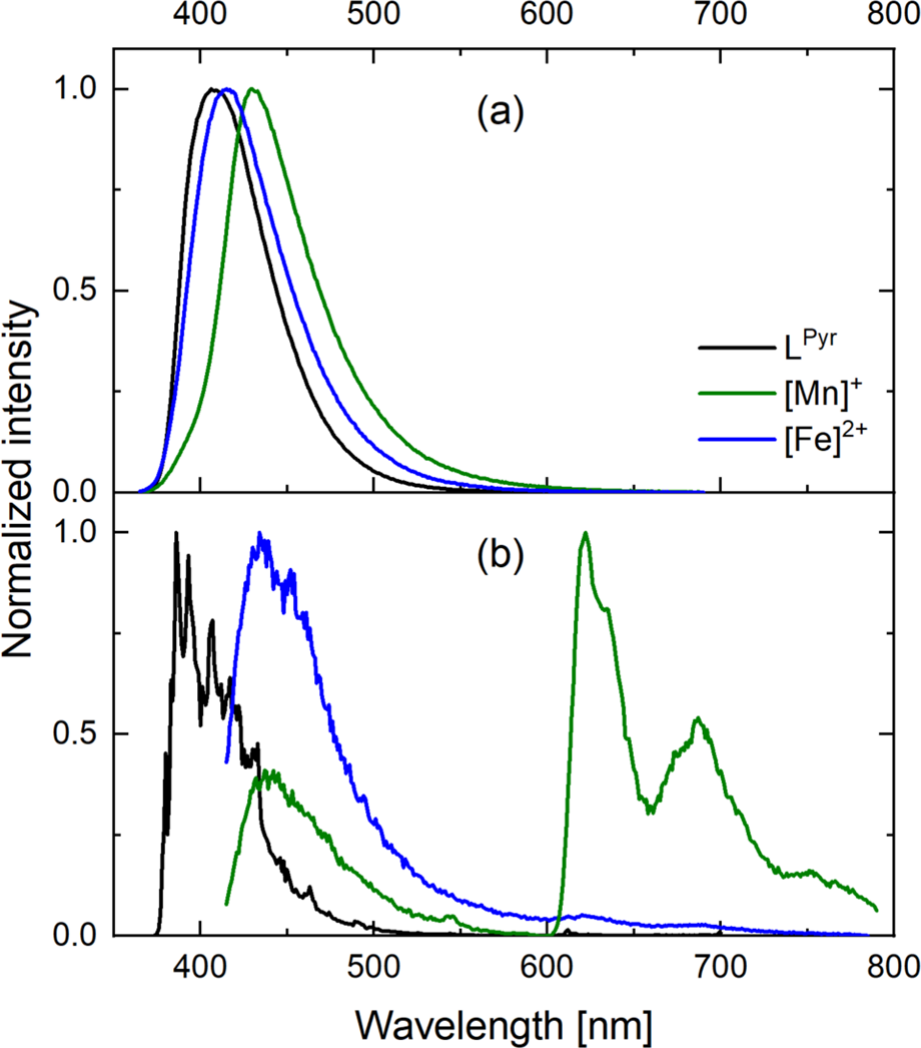
Emission spectra of L^Pyr^, [Mn]^+^, and [Fe]^2+^ in (a) aerated THF at room temperature and (b) frozen deaerated 2-MeTHF at 77 K. Excitation occurred at 350 nm for the room temperature measurements. For the 77 K experiments, excitation occurred at 350 nm for L^Pyr^ and at 400 nm for [Mn]^+^ and [Fe]^2+^.

Excitation of the uncoordinated ligand L^Pyr^ at 350 nm results in blue fluorescence with a band maximum at 410 nm in THF (Figure 5a, black trace). The luminescence lifetime and quantum yield are 4.2 ns and 86%, respectively (Figure S27, Tables S4 and S5). The excited state dynamics of L^Pyr^ was further explored with ultrafast transient absorption spectroscopy (Figure S29), and the following spectral features were identified: A ground state bleach (GSB) at 365 nm, excited state absorptions (ESAs) at 385, 515, and 650 nm, as well as stimulated emission (SE) at 430 nm (Figures S31 and S32). SE is a unique feature of the relaxed S_1_ state, and it is not detected until several picoseconds after excitation of L^Pyr^ (Figure S30). This observation indicates that higher excited states above the S_1_ state are populated at the earliest times. The kinetic profile of the ESA at 650 nm is identical with that of the SE (Figure S30), i.e., the ESA at 650 nm likewise is a spectral feature linked to the population of the relaxed S_1_ state of L^Pyr^. Three time constants were determined in the global fit analysis: 5.7 ps, 125 ps, and 4.8 ns. The slowest time constant is in good agreement with the fluorescence lifetime of 4.2 ns. The shorter two time constants are attributed to internal conversion (IC) from higher-lying S_*n*_ states to higher vibrational levels of S_1_, and to vibrational cooling within the S_1_ state, respectively. Expectedly, the population of higher singlet excited states is diminished when excitation occurs at 370 nm instead of 350 nm, and the fastest time component is reduced to 1.3 ps, whereas the two other time constants are unaffected by this change of the excitation wavelength (Table S6).

#### Chromium(0) Complex

**Figure 6:**
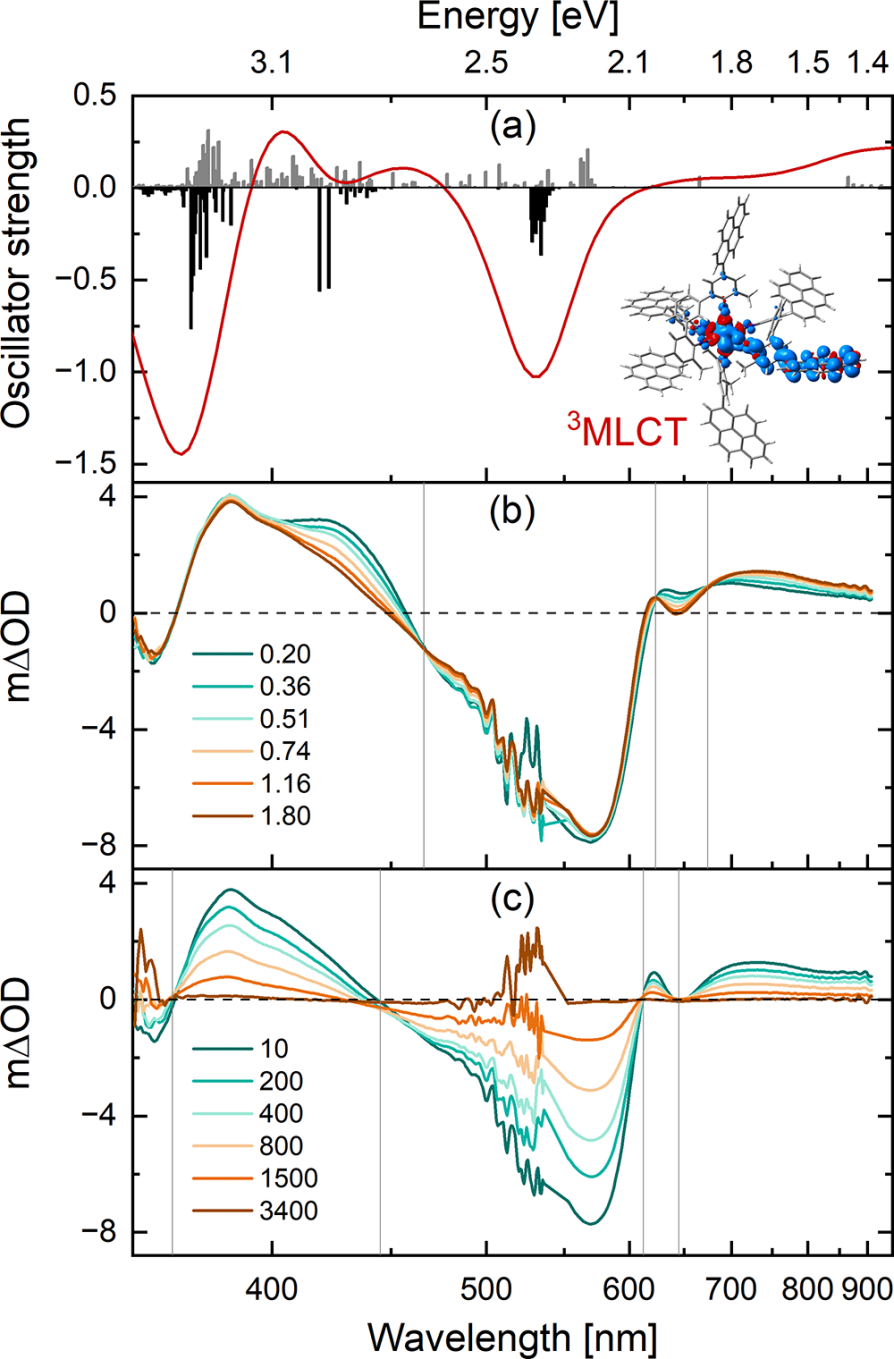
(a) Simulated transient absorption spectrum of [Cr] obtained at the TDDFT level of theory in THF (red, Lorentzian broadening with a full width half-maximum of 0.2 eV). The ESA (gray) is estimated by spin and dipole-allowed triplet–triplet transitions as obtained within the fully relaxed T_1_ structure. Contributions of GSB (black) are given by the dipole-allowed singlet–singlet transitions at the Franck–Condon point. Inset: spin density of the T_1_ state, indicating ^3^MLCT_Pyr_ character. Transient absorption spectra at early (b) and longer (c) time scales of [Cr] in a deaerated solution of THF at 20 °C. Excitation occurred at 550 nm. Delay times are given in picoseconds and their corresponding color codings are shown in the insets. Data points recorded between 0 and 200 fs after the laser pulse were omitted due to coherent artifacts at these short time scales. Gray vertical lines indicate isosbestic points. The wavelength scale on the horizontal axis is scaled such that the spectra appear correctly on an energy scale.

Excitation of [Cr] into the MLCT absorption band at 550 nm results in a broad unstructured luminescence in deaerated THF at 20 °C with a maximum of 840 nm (λ_em,MLCT_, Figure S37). In full agreement, the quantum chemical simulations assign the (fully equilibrated) lowest triplet state in [Cr] to be of ^3^MLCT_Pyr_ character and an emission wavelength of 793 nm is calculated (Δ*S*CF approach, Tables S22 and S25, and T_1_ spin density in Figure 6a). The luminescence quantum yield in deaerated THF is 0.01% (Figure S38), which is 2 orders of magnitude lower than in cyclohexane (1.04%).^51^ This drastic decrease in luminescence quantum yield upon changing the solvent seems attributable to the much lower ^3^MLCT energy in the more polar solvent THF, caused by better stabilization of the charge-redistribution and manifesting in a redshift of the MLCT emission band maximum of roughly 2100 cm^–1^ between cyclohexane and THF. According to the energy gap law, such a significant redshift is normally linked to a decrease in the luminescence quantum yield.^64^ Supporting, the nonradiative decay rate constant in THF is 45 times larger than in cyclohexane (Table S7). In contrast, the radiative decay rate is largely solvent-insensitive. The pronounced solvatochromic effect observed in the emission spectra, but not in the absorption spectra, of [Cr] is evidently due to a large change in the dipole moment between the ground state and the ^3^MLCT_Pyr_ excited state. Corroborating, DFT shows that structural relaxation of the T_1_ state (^3^MLCT, inset in Figure 6a) is associated with a reduction of the molecular symmetry from the initial D_3_ point group within the Franck–Condon geometry. In consequence, this ^3^MLCT state involves only one pyrene moiety, and the large distance between the Cr-centered hole and the electron within the respective π*_Pyr_ orbital leads to a pronounced dipole moment in the ^3^MLCT state, and, thus to the experimentally observed emission solvatochromism.

For [Cr], the lowest-lying ^1^MLCT state involves mostly the pyrene moieties rather than the diisocyanide *m*-terphenyl framework (Table S19), and the lowest ^3^MLCT state (inset in Figure 6a) is delocalized through one branch of the chelating ligands, with strong involvement of the tethered pyrene. This involvement of the pyrene moieties in both the key ^1^MLCT and the ^3^MLCT states of [Cr] is interesting because it stands in contrast to the behavior of pyrene-decorated ruthenium(II) polypyridines, where the pyrene moieties typically serve as isoenergetic triplet ligand-centered states feeding the ^3^MLCT state over time.^65,66^ In that way, the pyrene moieties act as reversible triplet energy reservoirs improving the photophysical properties of the emissive ^3^MLCT states of the ruthenium(II) complexes, but without directly mixing with the MLCT state. For [Cr], it is, however, rather the direct involvement of the pyrene moiety in the MLCT state that improves the photophysical properties through the delocalization effect. The optimized DFT structure of [Cr] shows a noncoplanar arrangement between the pyrene moieties and the aryl ring of the *m*-terphenyl backbone in the ground state. Evidently, and in accordance with our previous report on a related pyrene-decorated chromium(0) complex^50^ and previous studies of aryl-substituted [Ru(bpy)_3_]^2+^ derivatives,^67,68^ the pyrene subunits adopt a coplanar arrangement to the diisocyanide framework upon excitation, thereby allowing for a greater electron delocalization over the entire π-system.

The excited state dynamics of [Cr] at picosecond time scales remained unexplored in our earlier studies;^51^ hence, ultrafast transient absorption spectroscopy studies in deaerated THF were included in the present study (Figures 6 and S39). The transient absorption spectrum recorded immediately after excitation at 550 nm is dominated by GSBs at 360 and 570 nm as well as ESAs at 400 and 700 nm (Figure 6b). The ESA band at 400 nm has a shoulder at 425 nm, which disappears within the first few picoseconds after excitation, alongside a redshift and an increase of the less prominent ESA bands near 700 nm. In this early time window monitoring the excited state dynamics of [Cr], several isosbestic points (gray vertical lines) are identified, pointing to a state-to-state transition. Importantly, the amplitudes of the GSBs do not change during these first few picoseconds, indicating that this state-to-state transition does not involve repopulation of the ground state. On longer time scales (Figure 6c), relaxation back to the electronic ground state takes place, which is reflected in identical decay profiles of the GSBs and ESAs (Figure S43) as well as four isosbestic points at zero differential absorption. Based on the simulated transient absorption spectrum (Figure 6a), we associate the ESA band at ∼700 nm to ^3^IL_Pyr_ and ^3^ILCT transitions involving the photoreduced pyrene ligand (Figure S80a, Tables S22 and S25). The ESA at ∼400 nm originates from a plethora of transitions and was not characterized further.

Two time constants were obtained from the global fit analysis: 0.9 ps and 1.0 ns (Figure S42). The slower component reflects the deactivation of the ^3^MLCT excited state of [Cr] to the ground state and is in good agreement with the previously determined luminescence lifetime of 1.1 ns in THF. The faster component of 0.9 ps is attributed to vibrational relaxation from a “hot” ^3^MLCT excited state to the lowest vibrational level of the ^3^MLCT excited state. The time constant associated with intersystem crossing (ISC) from the ^1^MLCT to the ^3^MLCT excited state for related d^6^ metal complexes has previously been determined to take place within 200 fs,^40,69−73^ which is below the time resolution of our equipment.

#### Manganese(I) Complex

**Figure 7:**
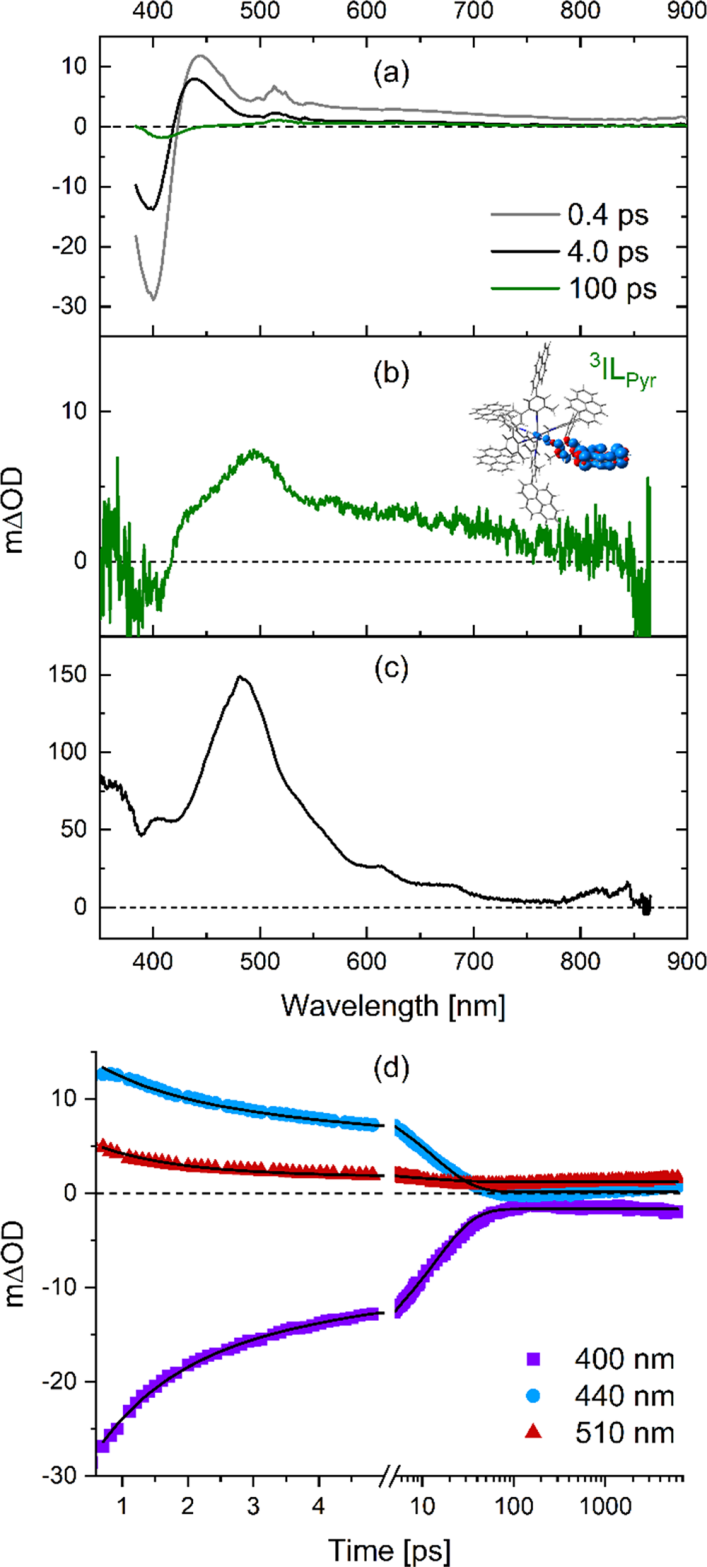
(a) Transient absorption spectra of [Mn]^+^ in deaerated THF at 20 °C at early time scales. Delay times are given in the inset. Excitation occurred at 370 nm. (b) Transient absorption spectrum of [Mn]^+^ in deaerated THF at 20 °C, where the signal was time-integrated for 200 ns immediately after the laser pulse. Excitation occurred at 430 nm. Inset: calculated spin density of the T_1_ state in [Mn]^+^, indicating ^3^IL_Pyr_ character. (c) Sensitized triplet absorption spectrum of L^Pyr^ in deaerated THF, recorded after excitation of an iridium(III) triplet sensitizer at 450 nm and subsequent triplet–triplet energy transfer to L^Pyr^; see Figures S33–S36 for further details. (d) Measured kinetics (symbols) and the result of the global fit analysis (solid lines) at selected wavelengths (see the inset) for [Mn]^+^ in deaerated THF. Excitation occurred at 370 nm.

The excited state dynamics of [Mn]^+^ in deaerated THF were explored by ultrafast transient absorption spectroscopy and TDDFT. Both excitation at 400 and 370 nm allowed for selective excitation of the mixed IL/MLCT excited state (Figures S51 and S52) without any significant population of higher-lying excited states. In the following, we focus on the data obtained with excitation at 370 nm because a larger part of the spectral region of interest is detectable when using this excitation wavelength than when using 400 nm irradiation (due to scattering of the excitation light). The initial transient absorption spectrum shows a GSB at 400 nm and ESAs with maxima at 445 and 520 nm (Figure 7a, gray). A spectral shift of the ESA from 445 to 440 nm occurs within the first few picoseconds (Figure 7a, black), and one isosbestic point at 460 nm is identified (Figure S54a). At later times (Figure 7a, green), the amplitudes of the GSB at 400 nm and ESA at 440 nm decrease; however, full ground state recovery of [Mn]^+^ does not occur within the 6 ns time window of this ultrafast experiment. Transient absorption spectroscopy on longer time scales yielded a lifetime of 2.9 μs in deaerated THF for the more slowly decaying spectral features (Figure S50). The transient absorption spectrum of [Mn]^+^ on the microsecond time scale shows great resemblance to the broad unstructured absorption band at 500 nm associated with the lowest triplet state found in the free L^Pyr^ ligand (compare Figure 7b,c). This observation strongly suggests that the slowest decay component of [Mn]^+^ is due to a low-lying triplet state localized on the L^Pyr^ ligand (Figure 4c). This analysis is further supported by our TDDFT simulations that clearly reveal that the thermally equilibrated T_1_ state is of ^3^IL_Pyr_ character as shown by the spin density in Figure 7b, and the observed ESA at ∼500 nm mainly stems from one ^3^IL_Pyr_ state (T_32_ at 539 nm, Figure S80b, Tables S23 and S26). Consequently, the decay of the T_1_ (^3^IL_Pyr_) state to the singlet ground state is spin-forbidden due to the weak contribution from spin–orbit coupling,^74−76^ leading to the observed microsecond lifetime.

The amplitude of the GSB signal of [Mn]^+^ is significantly reduced within the first 100 ps (Figure 7a) alongside the formation of the spectral feature related to the microsecond component identified as a ^3^IL_Pyr_ state (compare green in Figure 7a with Figure 7b). The ^3^IL_Pyr_ spectrum does not have intense absorption around 400 nm (Figure 7c), suggesting that the decay of the GSB signal cannot be explained by a growing absorption in the same spectra range. From this, we conclude that a significant portion of the ^3^IL/^3^MCLT excited state population returns to the ground state within the first 100 ps, whereas only a minor fraction populates the ^3^IL_Pyr_ state. Based on this interpretation of the experimental observations, the transient absorption data of [Mn]^+^ was modeled with a branching deactivation pathway (see page S49 for further details). Global fit analysis (Figure 7d) of the transient absorption spectra obtained within the first 6 ns resulted in the three time constants: 1.2, 16, and 90 ps (Figure S55). Analogously to [Cr], the shortest time constant is ascribed to vibrational relaxation of a “hot” ^3^IL/^3^MLCT excited state. The 16 ps time constant is associated with decay from the vibrationally cooled ^3^IL/^3^MLCT state directly to the ground state, whereas the 90 ps time constant is attributed to IC to the above-mentioned ^3^IL_Pyr_ state (Figure 4c). The total decay rate constant of the ^3^IL/^3^MLCT state is the sum of the two individual decay components, i.e., 0.072 ps^–1^, equivalent to an overall lifetime of 14 ps, and thus the ^3^IL/^3^MLCT lifetime of [Mn]^+^ is a roughly factor of 100 shorter than the ^3^MLCT lifetime of [Cr] under identical conditions (1.1 ns). The direct decay to the ground state is the dominant deactivation pathway from the ^3^IL/^3^MLCT state of [Mn]^+^, accounting for approximately 85%. The time constant of 90 ps for the IC from ^3^IL/^3^MLCT to ^3^IL_Pyr_ seems relatively slow compared to other metal complexes,^77^ and could have its origin in very weak electronic coupling between the respective two excited states. Corroborating our overall analysis, the species-associated spectrum of the third (temporally nonresolvable) decay component in the global fit analysis of the ultrafast experiment resembles well the transient absorption spectrum of [Mn]^+^ time-integrated over the first 200 ns (Figure S56).

Two manganese(I) complexes with chelating arylisocyanide ligands have previously been shown to have emissive ^3^MLCT excited states at room temperature in solution,^47^ whereas a manganese(I) complex with monodentate arylisocyanide ligands was nonemissive due to photodissociation.^55^ For [Mn]^+^, no ^3^IL/^3^MLCT luminescence was observed at room temperature, but the emission spectrum in a frozen matrix at 77 K shows two structured emission bands at 435 and 610 nm (Figure 5b, green). The shorter-wavelength emission band resembles the fluorescence spectrum of the free ligand L^Pyr^ (Figure 5b, black) and therefore is assigned to ligand-based fluorescence. Similar ligand-based fluorescence has been previously reported for selected iron(II) complexes^78,79^ and likely occurs as a result of electronic decoupling between ligand-based and metal-based electronic excited states, here to the extent that fluorescence becomes competitive. The structured emission at 610 nm is attributed to phosphorescence from the low-lying ^3^IL_Pyr_ state identified for [Mn]^+^ by transient absorption spectroscopy at room temperature (Figure 7b). Corroborating, TDDFT simulations identify a ^3^IL_Pyr_ emission at 627 nm in [Mn]^+^ (Table S23). Analogous ligand-based phosphorescence was also observable at 77 K for the two above-mentioned manganese(I) complexes with arylisocyanide chelates.^47^ In contrast to the manganese(I) complexes, iridium(III) complexes (5d^6^) have previously been reported to show isocyanide-based phosphorescence at room temperature.^80,81^

Evidently, the ligand pyrene decoration improves the visible-light absorption properties for [Mn]^+^ relative to previously used ligand designs for manganese(I) arylisocyanide complexes^47,55^ yet at the same time leads to a low-lying ^3^IL_Pyr_. Population of ^3^IL_Pyr_ from the ^3^IL/^3^MLCT state is a competing deactivation pathway (15%) to relaxation directly back to the ground state (85%). In contrast, there is no evidence for population of a ^3^IL_Pyr_ state in [Cr], indicating that the energy of the ^3^IL_Pyr_ state is higher than that of the emissive ^3^MLCT excited state in [Cr]. The phosphorescence at 610 nm associated with the ^3^IL_Pyr_ state, detected for [Mn]^+^, indirectly supports this conclusion, and TDDFT further clarifies, as the lowest ^3^IL_Pyr_ state in [Cr] is found at 624 nm (Table S16). In [Fe]^2+^, the performed TDDFT simulations predict the lowest-lying transitions to be of dominantly ILCT with minor admixed metal character and of similarly high energy as the mixed IL/MLCT state in [Mn]^+^ (Figures 3 and 4); hence, population of the low-lying ^3^IL_Pyr_ state, analogously as in [Mn]^+^, is expectable for [Fe]^2+^. However, the emission spectrum of [Fe]^2+^ at 77 K (Figure 5b, blue) does not show a phosphorescence band at 610 nm. This observation suggests that low-lying MC states play a key role in the excited-state deactivation of [Fe]^2+^, either leading to no population of the ^3^IL_Pyr_ state at all or involving rapid depopulation of this state even at low temperatures. The energy of the ^3^IL_Pyr_ state is not expected to change significantly among the investigated three homoleptic 3d^6^ complexes, and, in alignment with this expectation, a ^3^IL_Pyr_ emission was computationally predicted at 636 nm for [Fe]^2+^ (Table S24).

#### Iron(II) Complex

The ^1^ILCT absorption band in [Fe]^2+^ overlaps with the ^1^IL_Pyr_ transition at 350 nm (Figures 3, 4d and Table S18); hence, selective excitation of these individual electronically excited states is not possible. Consequently, probing the excited state dynamics of [Fe]^2+^ in THF at room temperature with ultrafast transient absorption spectroscopy reveals strong excitation wavelength-dependent behavior (Figures S64 and S65). Depending on whether excitation occurs at 370, 390, 410, or 430 nm, the observable ESA bands between 500 and 900 nm differ substantially in bandwidth and energetic position, suggesting that on the ultrafast time scale immediately after excitation, the initial population ratio between the ^1^IL_Pyr_ and ^1^ILCT excited states changes. In line with this scenario, an isosbestic point at 700 nm is observed when overlaying the initial spectra at different excitation wavelengths, indicating that two (and likely not more) different excited states are initially populated. IC between the ^1^IL_Pyr_ and ^1^ILCT states appears to be slow; hence, we observe overlapping contributions from the individual depopulation of these two states, including emission from the ^1^IL_Pyr_ state (vide infra). Following excitation of [Fe]^2+^ at 370 nm, the initial transient absorption spectrum with its ESA band maximum at 650 nm resembles the ESA feature at 650 nm observed for free L^Pyr^ ligand after excitation at 370 nm (Figure S65a). The ESA signals of [Fe]^2+^ and L^Pyr^ at 650 nm decay similarly on the nanosecond time scale (Figure S66), but on the subpicosecond time scale an additional short-lived component is present in [Fe]^2+^ (Figure S65b). Nonetheless, it seems plausible to conclude that excitation of [Fe]^2+^ at 370 nm populates predominantly a ligand localized excited state without metal contribution, similar in character to that in the free (uncoordinated) ligand, i.e., ^1^IL_Pyr_. Excitation of [Fe]^2+^ at longer wavelengths than 370 nm leads to ESA band maxima that gradually blueshift with increasing excitation wavelength (Figure S65), likely reflecting an increasing proportion of the initial ^1^ILCT population. Simultaneously with the increasing excitation wavelength, the relative contribution of the initial subpicosecond decay component increases. ISC between charge transfer states of iron(II) complexes occurs below 100 fs,^70,82^ which is faster than the time resolution of our experiment. Indirectly, this suggests that the subpicosecond time component observed for [Fe]^2+^ can be attributed to vibrational cooling from a “hot” ^3^ILCT to the relaxed ^3^ILCT state, analogously to [Cr] and [Mn]^+^ (Figures 6 and 7). The subsequent decay of the relaxed ^3^ILCT of [Fe]^2+^ could potentially take place through the population of a ^3^IL_Pyr_ state, as observed for [Mn]^+^, and/or through low-lying MC states. Extrapolating the relative energies of the ^3^MC states estimated for [Cr] and [Mn]^+^ to [Fe]^2+^ (Figure 4), we expect the population of MC states to play a key role in the deactivation of [Fe]^2+^. We did not investigate relaxed ^3^MC states computationally due to the size of our systems, but the fact that the overall excited state dynamics of [Fe]^2+^ and [Mn]^+^ are different indirectly supports the involvement of MC states for [Fe]^2+^. In other words, we would expect the excited state dynamics of [Fe]^2+^ to resemble those of [Mn]^+^ more closely if the ^3^MC state would not be populated in [Fe]^2+^. Rate constants for the individual excited-state relaxation processes in [Fe]^2+^ cannot be determined unambiguously, but this does not affect the overall semiquantitative picture in Figure 4d.

Upon excitation at 350 nm, both [Fe]^2+^ and [Mn]^+^ show unstructured emission at room temperature similar to L^Pyr^ (Figure 5a and Table 2), but the emission band maxima are red-shifted by 300 and 1300 cm^–1^, respectively. The excitation spectra monitoring the emission intensity as a function of irradiation wavelength match the UV–vis absorption spectra of [Mn]^+^ and [Fe]^2+^ well in the region of the absorption bands at 350 nm (Figures S48 and S62), but the agreement is poorer in the lower-energy spectral region. This is fully compatible with room temperature fluorescence of IL_Pyr_ character in [Mn]^+^ and [Fe]^2+^ (Figure 4), and the red-shifted nature of the emission bands is due to stabilization of the relevant π*-orbitals upon complexation with the Lewis acidic metals.^83^ The luminescence lifetime and the luminescence quantum yield for [Fe]^2+^ are 3.8 ns and 14%, respectively, and 3.1 ns and 2%, respectively, for [Mn]^+^ (Tables S8, S10 and 2), meaning that the fluorescence lifetimes are only slightly shorter in these complexes relative to L^Pyr^ (τ = 4.2 ns). However, the luminescence quantum yields are strongly reduced relative to uncoordinated L^Pyr^ (ϕ = 86%), indicating that only a fraction of the absorbed photons of [Mn]^+^ and [Fe]^2+^ populates the emissive ^1^IL state, for example due to spectral overlap with other transitions or due to initial population of higher excited state(s) whose relaxation branches between populating the ^1^IL state and the energetically lower-lying charge transfer excited state in these two complexes, mixed ^1^IL/^1^MLCT and ^1^ILCT, respectively. Given the nanosecond fluorescence lifetimes, IC from the initially excited ^1^IL_Pyr_ state to the singlet charge transfer states is surprisingly slow, likely due to very weak electronic coupling between the respective excited states. However, once the singlet charge transfer state is populated, ISC to the triplet charge transfer state is likely fast due to significant mixing with MC orbitals. Similarly, onward IC from the ^1^IL/^1^MLCT and ^1^ILCT states for [Mn]^+^ and [Fe]^2+^, respectively, to low-lying ^3^IL_Pyr_/^3^MC states is expected to be comparatively rapid (∼90 ps for [Mn]^+^), hereby providing efficient nonradiative deactivation pathways of the initially excited ^1^IL_Pyr_ state.

## Conclusions

**Figure 8:**
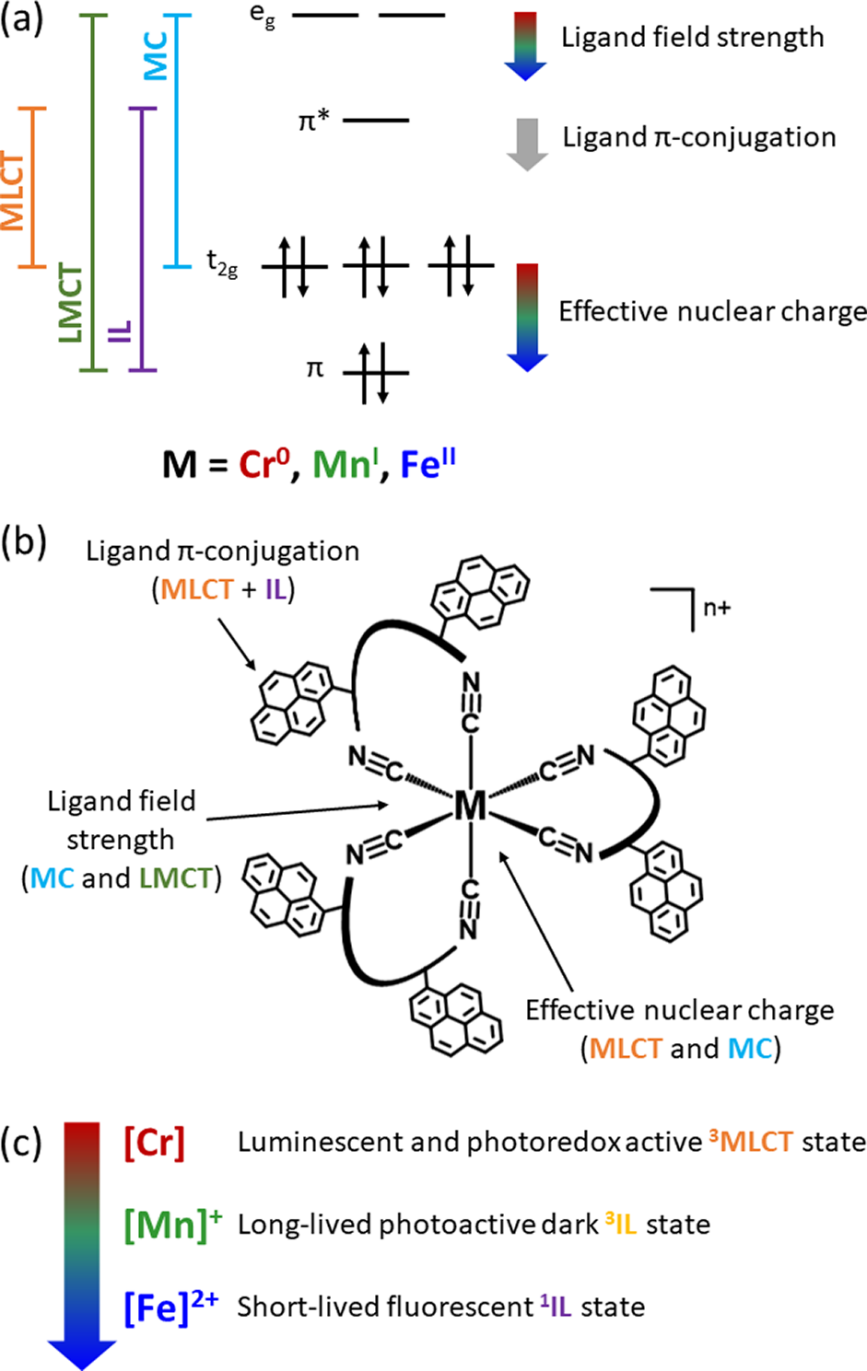
(a) Diagram depicting the frontier orbitals in low-spin 3d^6^ complexes exposed to a strong ligand field and the energy differences related to the MLCT (orange), LMCT (green), IL (purple), and MC (cyan) transitions. The energy levels of the metal-centered t_2g_ and e_g_ orbitals are governed by contributions from the effective nuclear charge of the metal and ligand field strength. Concurrent with the increase of the charge along the series chromium(0), manganese(I), and iron(II), both the t_2g_ and e_g_ orbitals are stabilized (red/green/blue downward arrows). Increased ligand π-conjugation stabilizes the energy of the ligand-centered π*-orbitals (gray arrow). (b) Generic molecular structure of the homoleptic tris(diisocyanide) complexes investigated in this study and the influence of the individual effects on the MLCT, IL, and MC energies (M = Cr^0^, *n* = 0), [Mn]^+^ (M = Mn^I^, *n* = 1), and [Fe]^2+^ (M = Fe^II^, *n* = 2). (c) Overall photophysical properties of [Cr], [Mn]^+^, and [Fe]^2+^ vary because of the combined contributions from the effective nuclear charge, ligand field strength, and ligand π-conjugation.

With classical polypyridine ligands, the comparison of photoactive d^6^ metal complexes with exact identical coordination environment is typically limited to two different oxidation states, for example between [Fe(bpy)_3_]^2+^ and [Co(bpy)_3_]^3+^, or between [Os(tpy)_3_]^2+^ and [Ir(tpy)_3_]^3+^.^30,33,84−87^ Owing to their combined σ-donor and π-acceptor properties,^88^ the isocyanide ligands used herein are able to accommodate a broader range of metal oxidation states, and the chelating nature of our ligands gives particularly robust homoleptic tris(bidentate) complexes of Cr^0^, Mn^I^ and Fe^II^. The combined NMR, theoretical and photophysical studies enabled by this special molecular design provide unusually clear insight into how effective nuclear charge, ligand field strength, and ligand π-conjugation affect the energetic order between MLCT, MC and IL excited states (Figure 8 and Table 2), and how these individual electronically excited states relax to the ground state (Figure 4). Understanding what factors govern these energy orders and elucidating the individual excited-state relaxation pathways is the key to obtaining photoactive metal complexes based on abundant first-row transition metal elements.^8,89,90^ The performed in-depth NMR studies furthermore provide insights into metal atom/ion size and rigidity effects in the electronic ground state.

The MLCT energy increases by 1.0 eV between [Cr] and [Mn]^+^, and by at least 0.5 eV between [Mn]^+^ and [Fe]^2+^ (Figure 4), due to the increasing effective nuclear charge. In parallel, the ligand field strengthens along this series, such that the energies of nonradiatively deactivating MC states raise concomitantly with the MLCT excited states. Pyrene-substitution of the diisocyanide ligands stabilizes the ligand-based π* orbitals, which is further helpful to install low-lying ^3^MLCT_Pyr_, and ^3^IL_Pyr_ states.

In [Cr], the energetic order of excited states is ^3^MLCT < ^3^MC < ^3^IL_Pyr_, leading to long-lived ^3^MLCT luminescence, whereas in [Mn]^+^ the energetic order changes to ^3^IL_Pyr_ < ^3^MLCT < ^3^MC (Figure 4). Consequently, the ^3^MLCT-type state in [Mn]^+^ is nonluminescent and deactivates in a branching fashion concurrently to the ground state (τ = 16 ps, k = 0.0625 ps^–1^) and to a lower-lying dark ^3^IL_Pyr_ state (τ = 90 ps, k = 0.011 ps^–1^). The lowest ^3^MC state remains at comparatively high energies, and population of the ^3^MC state in [Mn]^+^ seems negligible. In [Fe]^2+^, the energetic order further changes to ^3^IL_Pyr_ < ^3^MC < ^3^MLCT, which leads to rapid deactivation along the triplet manifold from the highly distorted ^3^MC excited state, precluding any noticeable population of the ^3^IL_Pyr_ state. Notably, the performed TDDFT simulations do not predict any ^3^MLCT state contribution within the first 300 triplet states (covering an excitation energy of 4.5 eV at the Franck–Condon point).

The comparatively large extended π-system created by pyrene decoration is helpful in [Cr] to reach a ^3^MLCT_Pyr_ lifetime and a photoluminescence quantum yield competitive with the [Os(bpy)_3_]^2+^ benchmark compound,^51^ and it improves the visible light absorption properties of [Mn]^+^ in comparison to related manganese(I) arylisocyanide complexes.^47,49,55^ Furthermore, it enables the installment of a dark ^3^IL_Pyr_ state with a microsecond lifetime in [Mn]^+^. Whereas in second- and third-row transition metal complexes the changeover from luminescent ^3^MLCT to emissive ^3^IL states has been accomplished in several cases, for example when going from [Ru(bpy)_3_]^2+^ to [Rh(bpy)_3_]^3+^, or between [Os(tpy)_3_]^2+^ and [Ir(tpy)_3_]^3+^,^84,86,87,91^ the present study illustrates that ^3^IL room temperature emission is more difficult to obtain from first-row d^6^ metal compounds, an aspect that has so far not received much attention.^13^ This is likely due to the nearby lying ^3^MC state, yet the comparison between [Mn]^+^ and [Fe]^2+^ suggests that as long as the respective ^3^MC state remains energetically above the lowest ^3^MLCT state, (dark) ^3^IL states with microsecond lifetimes are obtainable. The lack of emission from the ^3^IL state despite microsecond lifetimes indicates a very low radiative rate constant for relaxation to the ground state. This seems in line with a minor metal contribution to the respective ^3^IL state.

The IL states of our diisocyanide ligands (and in particular those with pyrene decoration, i.e., IL_Pyr_) appear to be electronically more strongly decoupled from excited states with metal contribution (MLCT, MC) than the IL states of polypyridine ligands in classical d^6^ metal polypyridine complexes, which could explain why IL fluorescence is a competitive deactivation process in some of our isocyanide complexes ([Mn]^+^, [Fe]^2+^), though ligand-based fluorescence has also been reported for other types of iron(II) complexes.^78,79^ The presumed relatively weak electronic coupling between states with different orbital character could furthermore be responsible for the relatively slow IC from the mixed ^3^IL/^3^MLCT state to the low-lying ^3^IL_Pyr_ state in [Mn]^+^ (90 ps).

A multifaceted overall picture emerges, in which effective nuclear charge, ligand field strength, and ligand π-conjugation control the excited-state ordering to an unprecedented extent in 3d^6^ metal complexes (Figure 8), resulting in rich photophysical behavior ranging from MLCT phosphorescence to a long-lived dark IL state and high-energy IL fluorescence (Figure 4). We hope these insights will enable further advances toward making 3d^6^ compounds fit for practical applications in light harvesting, luminescent devices, and photocatalysis.

## Supporting Information Available

Synthetic protocols and characterization data, description of equipment and methods as well as supplementary spectroscopic data (PDF)
