# Supplementary material for: Controlling the Photophysical Properties of a Series of Isostructural d6 Complexes Based on Cr0, MnI, and FeII
Source: J Am Chem Soc. 2024 Feb 9;146(7):4605–19. doi: 10.1021/jacs.3c11580 (PMC10885143; doi:10.1021/jacs.3c11580)
Supplement: Supplementary file 1 — ja3c11580_si_001.pdf [file ja3c11580_si_001.pdf]

Supporting Information to accompany:

Controlling the Photophysical Properties of a Series of Isostructural d^6 Complexes Based on Cr^0, Mn^I, and Fe^{II}

Christina Wegeberg,[1,2,*] Daniel Häussinger,[1] Stephan Kupfer,[3,*] Oliver S. Wenger[1,*]

[1] Department of Chemistry, University of Basel, St. Johanns-Ring 19, 4056 Basel (Switzerland)

[2] Current address: Chemical Physics, Department of Chemistry, Lund University, Box 124, 22100 Lund (Sweden)

[3] Institute of Physical Chemistry, Friedrich Schiller University Jena, Helmholtzweg 4, 07743 Jena (Germany)

Table of Contents

1. **Preparation and Materials** S3
2. **Instrumentation and Methods** S3
3. **Synthetic Procedures** S6
   - **3.1 [Mn]PF_6** S7
   - **3.2 [Fe]$(PF_6)_2$** S8
   - **3.3 [$FeCl_2$]** S8
4. **NMR Characterization** S9
   - **4.1 L^{Pyr}** S9
   - **4.2 [Cr]** S11
   - **4.4 $[Fe]^{2+}$** S18
   - **4.5 [$FeCl_2$]** S21
5. **Solid-State FTIR Spectra** S23
6. **High-Resolution ESI Mass Spectra** S25
7. **Cyclic Voltammetry** S26
8. **Photophysical Characterization** S27
   - **8.1 L^{Pyr}** S27
   - **8.2 [Cr]** S36
   - **8.3 $[Mn]^{+}$** S41
   - **8.4 $[Fe]^{2+}$** S52
   - **8.5 [$FeCl_2$]** S60
9. **Quantum chemistry** S66
   - **9.1 Computational Details** S66
   - **9.2 Franck-Condon Photophysics** S68
10. **References** S90

1. Preparation and Materials

Synthetic procedures were carried out under a nitrogen atmosphere using standard Schlenk techniques. All chemicals were purchased from commercial suppliers and used without further purification unless otherwise noted. Toluene and tetrahydrofuran (THF) were dried in a solvent purification system by Innovative Technology. L^{Pyr} and [Cr] were synthesized according to our previous report.[1] $[Os(bpy)_3](PF_6)_2$ (bpy = 2,2'-bipyridine) and *fac*-$[Ir(ppy)_3]_3$ (ppyH = 2-phenylpyridine) were synthesized following reported procedures.[2,3] [Cr] can as a solid be handled under ambient atmosphere, but upon dissolution, the chromium(0)-complex is unstable towards oxygen, hence solvents were purged with argon (5.0, PanGas) for 10 minutes prior to dissolution. The manganese(I) and the two iron(II) complexes are not air-sensitive.

2. Instrumentation and Methods

Variable temperature ^1H and ^{13}C NMR experiments were recorded on a Bruker Avance III NMR spectrometer operating at 600 MHz using an indirect BBI probe head, equipped with a self-shielded z-gradient. The temperature was calibrated using a methanol sample and showed an accuracy of ± 0.2 K. Routine, gradient selected 2D NMR experiments were used (COSY, NOESY, ROESY, HSQC, HMBC). Chemical shifts (δ) are denoted relative to TMS (δ = 0 ppm) and were calibrated using the residual solvent peak of 1,1,2,2-tetrachloroethane-d_2 (TCE-d_2, δ_H = 6.00 ppm, δ_C = 73.78 ppm). For the variable temperature experiments, the residual solvent peak of TCE-d_2 was fixed at the values given above. The coupling constants (J) of ^1H-^1H couplings are given in Hz and these are described by the following abbreviations: s (singlet), d (doublet), t (triplet) and m (multiplet). Elemental analysis was carried out on a Vario MICRO Cube instrument (Elementar). High resolution ESI-MS analysis (positive mode) was performed on a Bruker maXis 4G QTOF mass spectrometer coupled with Thermo Fischer UPLC. Solid-state FTIR spectroscopy was performed on a Bruker Alpha Platinum-ATR instrument, and the following abbreviations are used to describe the intensity of the vibrational bands: s (strong), m (medium) and w (weak). For cyclic voltammetry, a Versastat3-200 potentiostat from Princeton Applied Research was used. A platinum disk electrode served as a working electrode, the counter electrode and quasi-reference electrode were two silver wires. Internal potential calibration occurred by addition of small amounts of ferrocene. Dry deaerated THF containing 0.2 M ($^nBu_4N)PF_6$ (tetra-*n*-butylammonium hexafluorophosphate) was used as an electrolyte. Potential sweep rates were 100 mV/s. The cyclic voltammetry experiments were performed under an inert atmosphere (Ar or N_2).

UV–Vis absorption spectra were recorded on a Cary 5000 spectrophotometer (Varian). Steady-state emission and excitation spectra were recorded on either a Fluorolog-322 instrument (Horiba Jobin-Yvon)

equipped with iHR320, a Xenon lamp 450 W Illuminator (FL-1039A/40A) and a water-cooled photomultiplier tube (PMT Hamamatsu R13456), where the cuvette holder was equipped with a temperature controller (TC 1, Quantum Northwest) or a FP-8600 spectrophotometer (JASCO). Photoluminescence lifetime studies were performed on a FLS1000 spectrometer (time-correlated single photon counting (TCSPC) technique) from Edinburgh Instruments using a pulsed LED for excitation at 375 nm (Edinburgh Instruments, pulse width: 61.7 ps, linewidth <1.5 nm) or at 405 nm (Edinburgh Instruments, pulse width: 59.7 ps, linewidth <2.0 nm), where the cuvette holder was equipped with a temperature controller (TC 1, Quantum Northwest).

The fluorescence quantum yields (φ_F) of L^{Pyr}, $[Mn]^+$, $[Fe]^{2+}$, $[FeCl_2]$ in THF were determined relative to that of 9,10-diphenylanthracene (DPA) in cyclohexane ($\varphi = 0.91$)[4] after excitation at 350 nm. Eq. S1 was used for fluorescence quantum yield determination, where I is the integrated emission intensity, A is the absorbance at the excitation wavelength and η is the refractive index of the solvent ($\eta_{cyclohexane} = 1.4262$, $\eta_{THF} = 1.4072$).[5] Absorbance and emission measurements at five different concentrations were performed ($0.02 \leq A \leq 0.13$ at the excitation wavelength of 350 nm). The x-axes of the emission spectra were converted from wavelength (nm) to wavenumbers (cm^{-1}), and the y-axes of the emission spectra were adjusted following Eq. S2.[6]

$$\phi_F = \frac{\frac{I_F}{A_F}}{\frac{I_{DPA}}{A_{DPA}}} \cdot \phi_{DPA} \cdot \frac{\eta_{THF}^2}{\eta_{cyclohexane}^2} \qquad \text{(eq. S1)}$$

$$I(\bar{\nu}) = \lambda^2 I(\lambda) \qquad \text{(eq. S2)}$$

Transient absorption spectroscopy studies on time scales of >10 ns were performed on an LP920-KS instrument from Edinburgh Instruments at 20 °C on sample solutions in 1 cm cuvettes, where the sample holder was equipped with a temperature controller (TC 1, Quantum Northwest). Excitation occurred at either 430 nm or 450 nm using a frequency-tripled Nd:YAG laser (Quantel Brilliant, pulse width: ca. 10 ns, pulse frequency: 10 Hz) equipped with an OPO from Opotek, and the typical pulse energy was ca 7 or 15 mJ, respectively. Detection of transient absorption spectra occurred on an iCCD camera (Andor) with the time gates given at the respective measurements. Absorption kinetics at single wavelengths were recorded using a photomultiplier tube. Transient absorption studies with sub-nanosecond time resolution (1 – 20 ns) were performed on sample solutions in 1 cm cuvettes at room temperature using a TRASS instrument from Hamamatsu equipped with a streak camera and a mode-locked picosecond $Nd:YVO_4/YAG$ laser (model PL2251B-20-SH/TH/FH with PRETRIG option) as an excitation source. The laser pulse duration was ~30

ps and the pulse frequency was 10 Hz. The laser pulse energy of 8 mJ at 355 nm powered an Ekspla PG402-264 OPA with an energy output of ~1 mJ at 410 nm. Transient absorption spectra on picosecond time resolution were recorded on a commercial pump-probe setup from LIGHT CONVERSION, which consists of a PHAROS fs-laser, an OPA called ORPHEUS, a HARPIA spectrometer, and a Kymera 193i-B2 iCCD camera from Andor. PHAROS is a compact Yb:KGW (ytterbium-doped potassium gadolinium tungsten) femtosecond pulsed laser with a maximal repetition rate of 50 kHz / 10 W and a central wavelength of 1030 nm with a minimum pulse duration of 50 fs. In the current study repetition rates of 50 kHz / 10 W and 10 kHz / 2 W were used for $[Fe]^{2+}$ and $[Mn]^{+}$, respectively. When pumped with the PHAROS laser, the ORPHEUS OPA can emit radiation between 185 and 2000 nm with pulse energies of up to 0.7 mJ. Probe pulses for the spectral range of 350 nm to 550 nm were generated using the fundamental PHAROS radiation (1030 nm) and a 5 mm thick sapphire white light supercontinuum (WLSc). The spectral range of 550 to 850 nm was generated by using the second optical harmonic of PHAROS (515 nm) with the same 5 mm sapphire WLSc. fs-measurements were performed on sample solutions in 1 mm cuvettes at room temperature. The fs-datasets were chirp- and background-corrected with the CarpetView software provided by LIGHT CONVERSION, and Matlab (R2022b) was used for the global fit analysis.

3. Synthetic Procedures

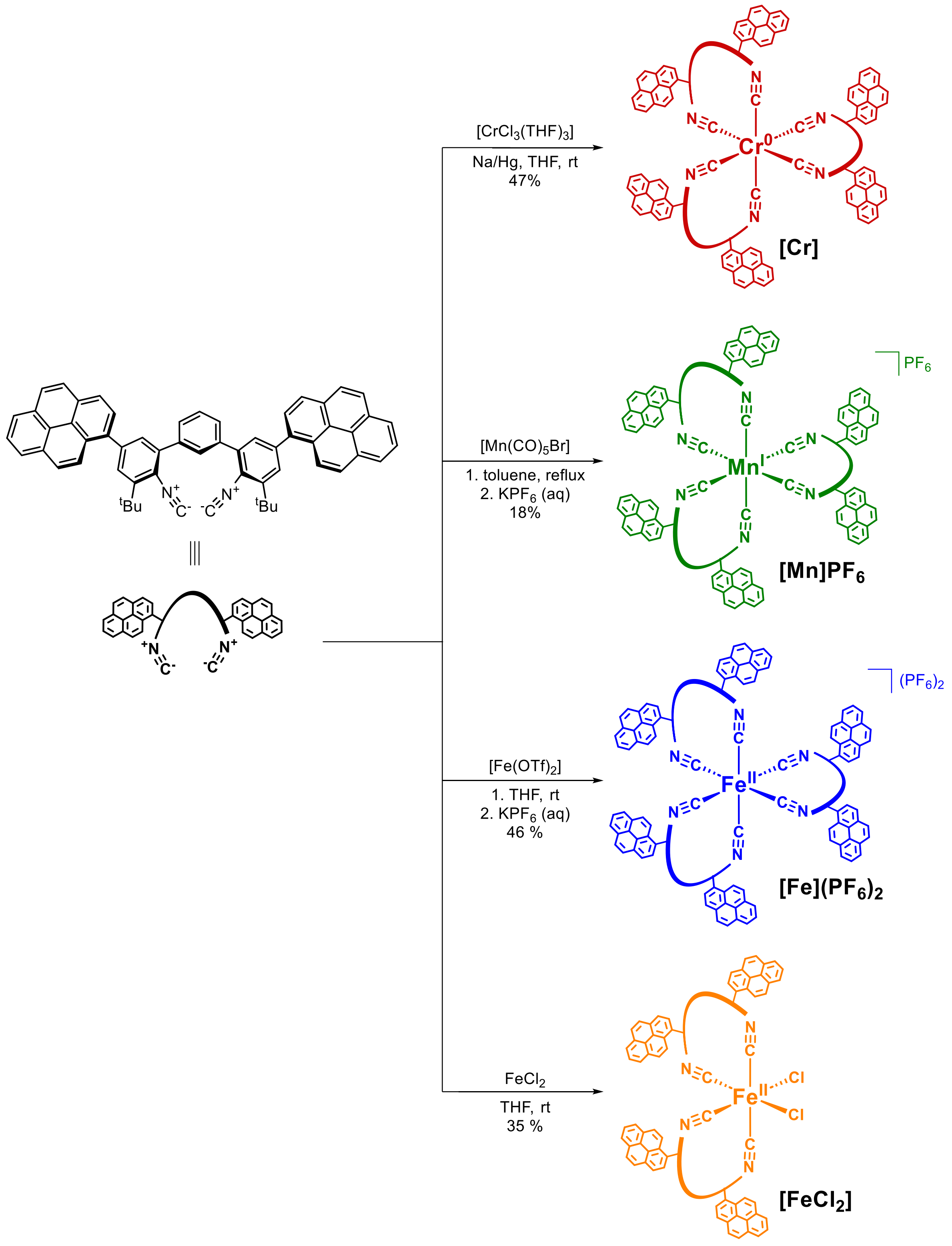

Scheme S1. Synthetic route to [Cr] (red), [Mn]PF_6 (green), [Fe]$(PF_6)_2$ (blue) and $FeCl_2$ (orange).

3.1 [Mn]PF_6

[$Mn(CO)_5Br$] (6.49 mg, 23.6 μmol, 1 eq.) and L^{Pyr} (59.9 mg, 75.5 μmol, 3.2 eq.) were dissolved in dry toluene (4 mL) under N_2 and refluxed at 110 °C overnight, whereupon a pale brown solid precipitated in an orange solution. The orange supernatant was removed by decantation, and the brown solid was dissolved in THF (2 mL). An aqueous solution (1 mL) of KPF_6 (17.4 mg, 94.4 μmol, 4 eq.) was added, and the solution was stirred for 1h. DCM (20 mL) was added, and the organic phase was washed with H_2O (20 mL) and aqueous NH_4PF_6 (20 mL), dried with Na_2SO_4 and concentrated to dryness. The crude was dissolved in THF (1 mL), and slow diffusion of *n*-pentane overnight at room temperature gave [Mn]PF_6 as a yellow solid, which was isolated by vacuum filtration. Yield: 10.9 mg (18 %).

FT-IR: ν 3040 (w), 2957 (w), 2863 (w), 2052 (s), 1587 (w), 1424 (w), 1364 (w), 1269 (w), 1242 (w), 1190 (w), 1064 (w), 895 (w), 837 (s), 817 (s), 806 (s), 750 (m), 722 (m), 710 (m), 683 (m), 661 (w), 632 (w), 598 (m), 590 (m), 579 (s), 556 (s), 521 (m), 503 (m), 450 (m) cm^{-1} .

Elemental analysis for $C_{180}H_{132}F_6MnN_6P$ ([Mn]PF_6) found (calc) C: 83.61% (83.83%), H: 5.19% (5.16%), N: 3.20% (3.26%).

High-resolution mass: *m/z* for $C_{180}H_{132}MnN_6$ ($[Mn]^+$) calc 2432.9922, found: 2432.9863

$E_{½}$ (Mn^I/Mn^{II}, THF): 0.68 V vs. Fc^0/Fc^+. $E_{½}$ ($L^{Pyr\bullet-}/L^{Pyr}$, THF): -2.12 V vs. Fc^0/Fc^+.

3.2 [Fe](PF_6)$_2$

Fe(OTf)$_2$ (7.2 mg, 20.3 µmol, 1 eq.) was dissolved in dry THF (2 mL) under N_2 and a solution of L^{Pyr} (50.2 mg, 63.3 µmol, 3.1 eq.) in dry THF (2 mL) was added, whereupon a color change to orange occurred. The reaction mixture was stirred for 8 hours at room temperature. KPF_6 (18.7 mg, 101 µmol, 5 eq.) in H_2O (1 mL) was added and the reaction mixture was stirred for an additional 1 hour. DCM was added, and the organic phase was extracted with H_2O (2 × 10 mL), dried with Na_2SO_4 and concentrated to dryness. The crude was dissolved in THF (1 mL), and slow diffusion of diethyl ether overnight at room temperature gave [Fe](PF_6)$_2$ as a yellow solid, which was isolated by vacuum filtration. Yield: 22.7 mg (46 %).

FT-IR: ν 3041 (w), 2959 (w), 2867 (w), 2159 (s), 1583 (w), 1482 (w), 1417 (w), 1405 (w), 1365 (w), 1273 (m), 1245 (m), 1153 (m), 1074 (w), 1028 (m), 967 (w), 893 (w), 841 (s), 817 (m), 806 (m), 755 (m), 722 (m), 711 (m), 685 (m), 663 (w), 631 (m), 598 (w), 556 (m), 517 (m), 482 (m) cm^{-1} .

Elemental analysis for $C_{180}H_{132}F_{12}FeN_6P_2$([Fe]($PF_6$)$_2$) found (calc) C: 79.69% (79.34%), H: 5.13% (4.88%), N: 3.14% (3.08%).

High-resolution mass: *m/z* for $C_{180}H_{132}FeN_6$ ($[Fe]^{2+}$) calc 1216.9944, found: 1216.9924.

3.3 [$FeCl_2$]

$FeCl_2$ (2.4 mg, 18.9 µmol, 1 eq.) was dissolved in dry THF (2 mL) under N_2 and a solution of L^{Pyr} (30.0 mg, 37.8 µmol, 2 eq.) in dry THF (2 mL) was added. The reaction mixture was stirred overnight under N_2 at room temperature. The volume was reduced to 1 mL, and slow diffusion of *n*-pentane overnight afforded [$FeCl_2$] as a yellow solid, which was isolated by vacuum filtration. Yield: 16.0 mg (35 %).

FT-IR: ν 3040 (w), 2959 (w), 2911 (w), 2872 (w), 2125 (s), 1584 (m), 1482 (w), 1423 (m), 1406 (w), 1367 (w), 1270 (w), 1244 (w), 1188 (w), 1074 (m), 1036 (w), 972 (w), 893 (w), 841 (s), 819 (m), 800 (m), 7550 (m), 722 (m), 711 (m), 682 (m), 662 (w), 636 (w), 598 (w), 587 (w), 563 (m), 552(m), 539 (m), 521 (w), 499 (w),456 (w) cm^{-1} .

Elemental analysis for $C_{120}H_{96}Cl_2FeN_4O_4$ ([$FeCl_2$](H_2O)$_4$) found (calc) C: 80.42% (80.75%), H: 5.09% (5.42%), N: 3.16% (3.14%).

High-resolution mass: *m/z* for $C_{120}H_{88}FeClN_4$ ($\{[FeCl_2] – Cl\}^+$) calc 1676.6074, found: 1676.6044

4. NMR Characterization

4.1 L^{Pyr}

The ligand L^{Pyr} was fully characterized with ^{1}H and ^{13}C NMR spectroscopy in tetrachloroethane-d$_2$ (TCE-d$_2$) to enable direct comparison with the manganese(I) and iron(II) complexes investigated in this study. L^{Pyr} has previously been characterized in C_6D_6.[1]

^{1}H NMR (600 MHz, TCE-d$_2$, 298 K) δ = 8.27 – 8.23 (m, 4H), 8.22 (d, J = 9.2 Hz, 2H), 8.19 (d, J = 7.6 Hz, 2H), 8.16 – 8.12 (m, 4H), 8.10 (d, J = 9.3 Hz, 2H), 8.07 – 8.04 (m, 4H), 7.82 (t, J = 1.8 Hz, 1H), 7.77 (dd, J = 7.8, 1.8, Hz, 2H), 7.73 (d, J = 1.9 Hz, 2H), 7.70 (d, J = 7.8 Hz, 1H), 7.69 (d, J = 1.9 Hz, 2H), 1.64 (s, 18H) ppm.

^{13}C{^{1}H} NMR (151 MHz, TCE-d$_2$, 298 K) δ = 172.7, 146.3, 141.7, 141.2, 138.3, 136.0, 131.3, 130.8, 130.6, 130.5, 130.3, 129.1, 128.4, 128.3, 128.1, 128.0, 127.8, 127.5, 127.3, 126.2, 125.4, 125.1, 124.74, 124.69, 124.6, 124.5, 122.2, 35.5, 29.4 ppm.

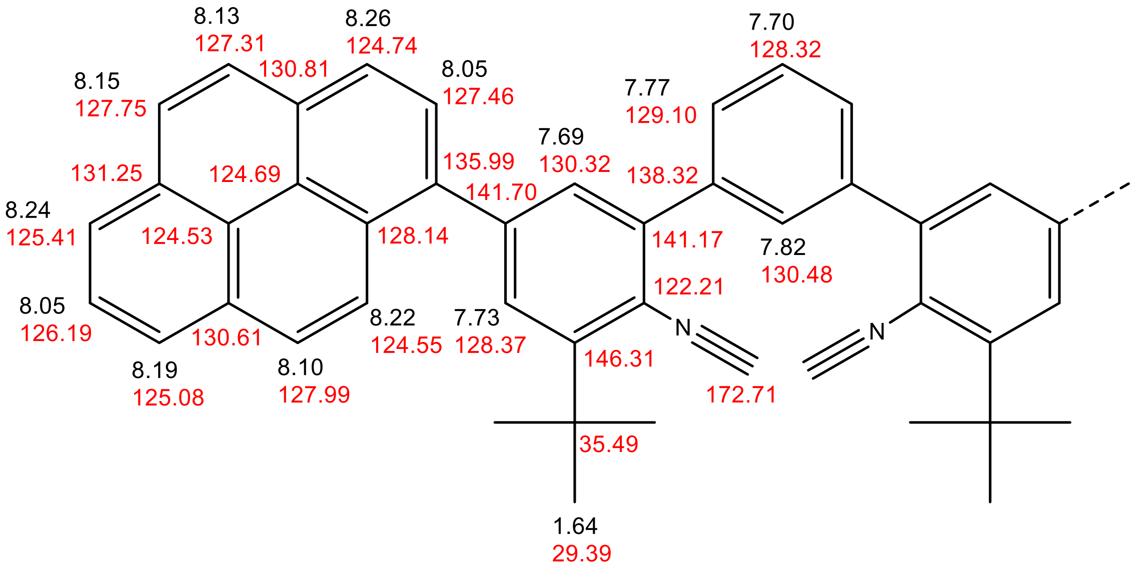

Figure S1. Assignment of the measured ^{1}H-NMR (black) and ^{13}C-NMR (red) resonances of L^{Pyr} in TCE-d$_2$ in Figures S2 and S3, respectively. The 2D-NMR techniques COSY and NOESY were used to assign the protons, and HSQC and HMBC were used to establish the coupling between ^{1}H and ^{13}C.

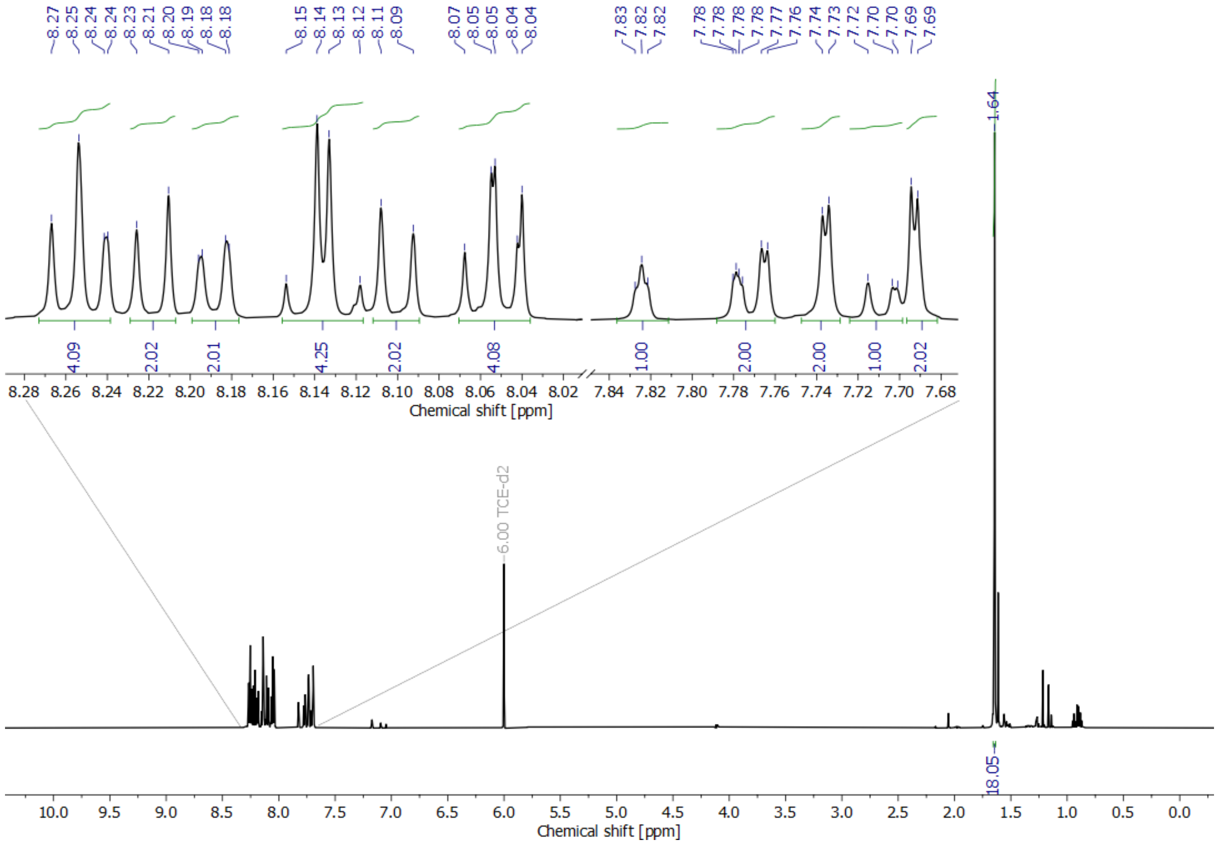

Figure S2. ^{1}H NMR spectrum of L^{Pyr} (600 MHz, TCE-d_2, 298K)

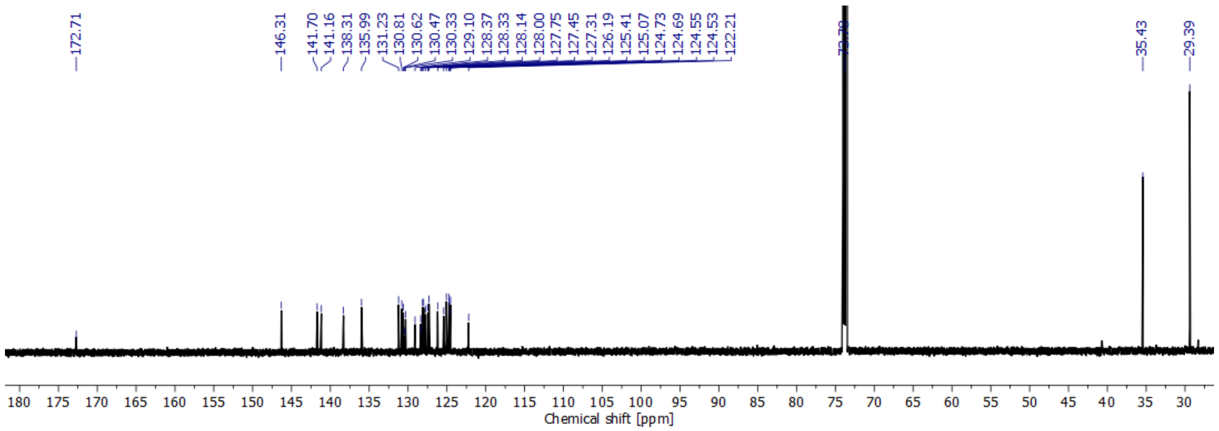

Figure S3. $^{13}C\{^{1}H\}$ NMR spectrum of L^{Pyr} (151 MHz, TCE-d_2, 298K). The resonance at 172.7 ppm originates from the isocyanide carbon.

4.2 [Cr]

The full ^{1}H and ^{13}C NMR analysis of [Cr] has previously been published (Figure S4),[1] and therefore only the temperature-dependent ^{1}H NMR spectra obtained at 298 K and 378 K are included here, enabling direct comparison of the dynamic behavior with the isostructural complexes $[Mn]^{+}$ and $[Fe]^{2+}$.

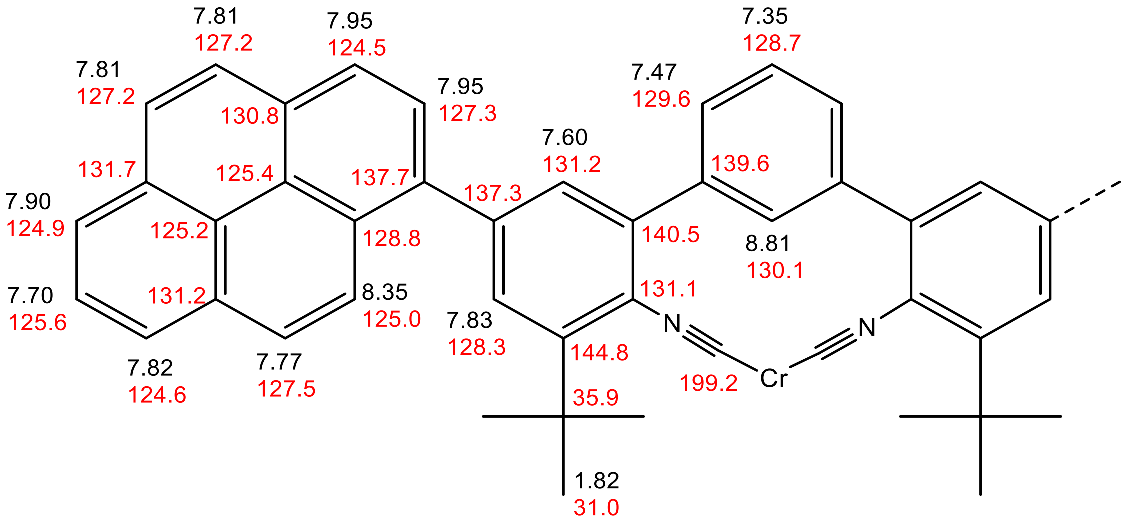

Figure S4. Assignment of the measured ^{1}H-NMR (black) and ^{13}C-NMR (red) resonances of [Cr] in toluene-d_8 (378K).

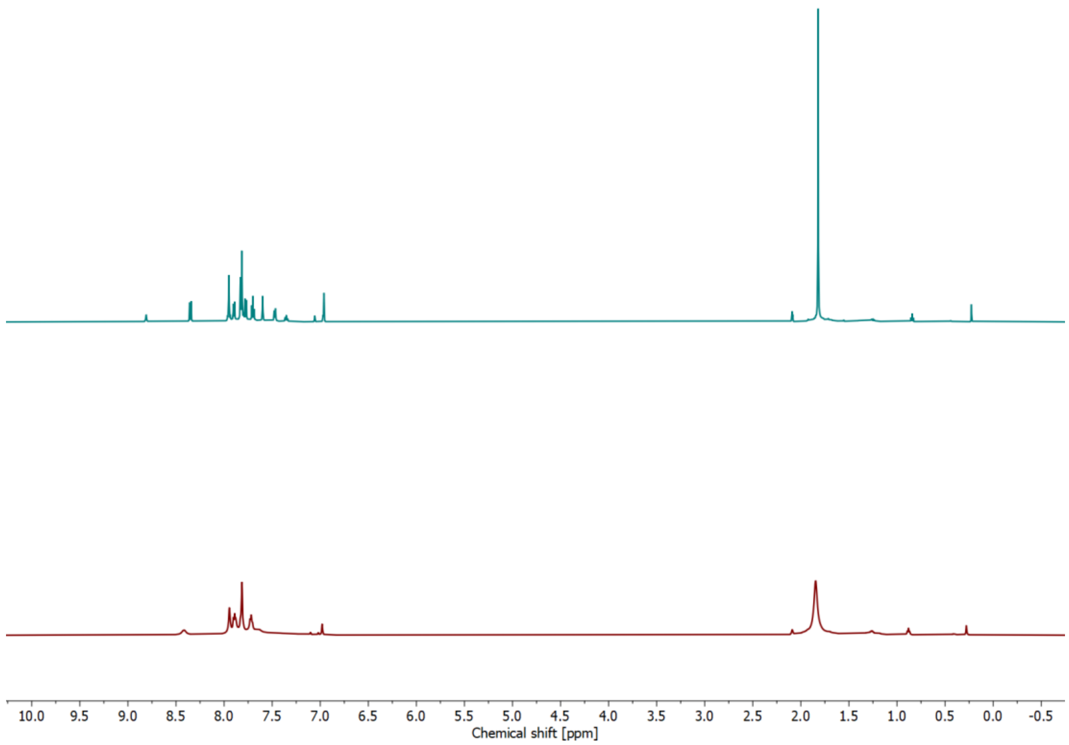

Figure S5. ^{1}H NMR spectrum of [Cr] in toluene-d$_8$ (600 MHz) at 298 K (bottom) and 378 K (top). The resonance at 1.82 ppm (378 K) originates from the *tert*-butyl groups.

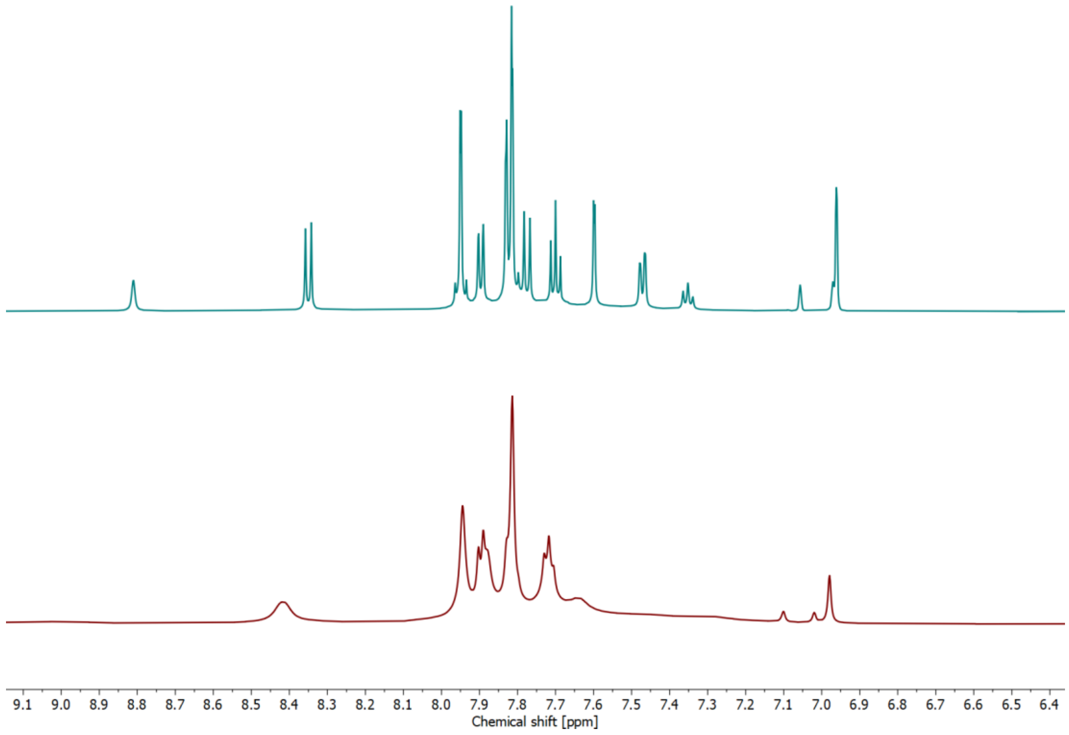

Figure S6. Zoom of the aromatic region in the ^{1}H NMR spectrum of [Cr] in toluene-d$_8$ (600 MHz) at 298 K (bottom) and 378 K (top). The three resonances around 7 ppm originate from the residual solvent peak.

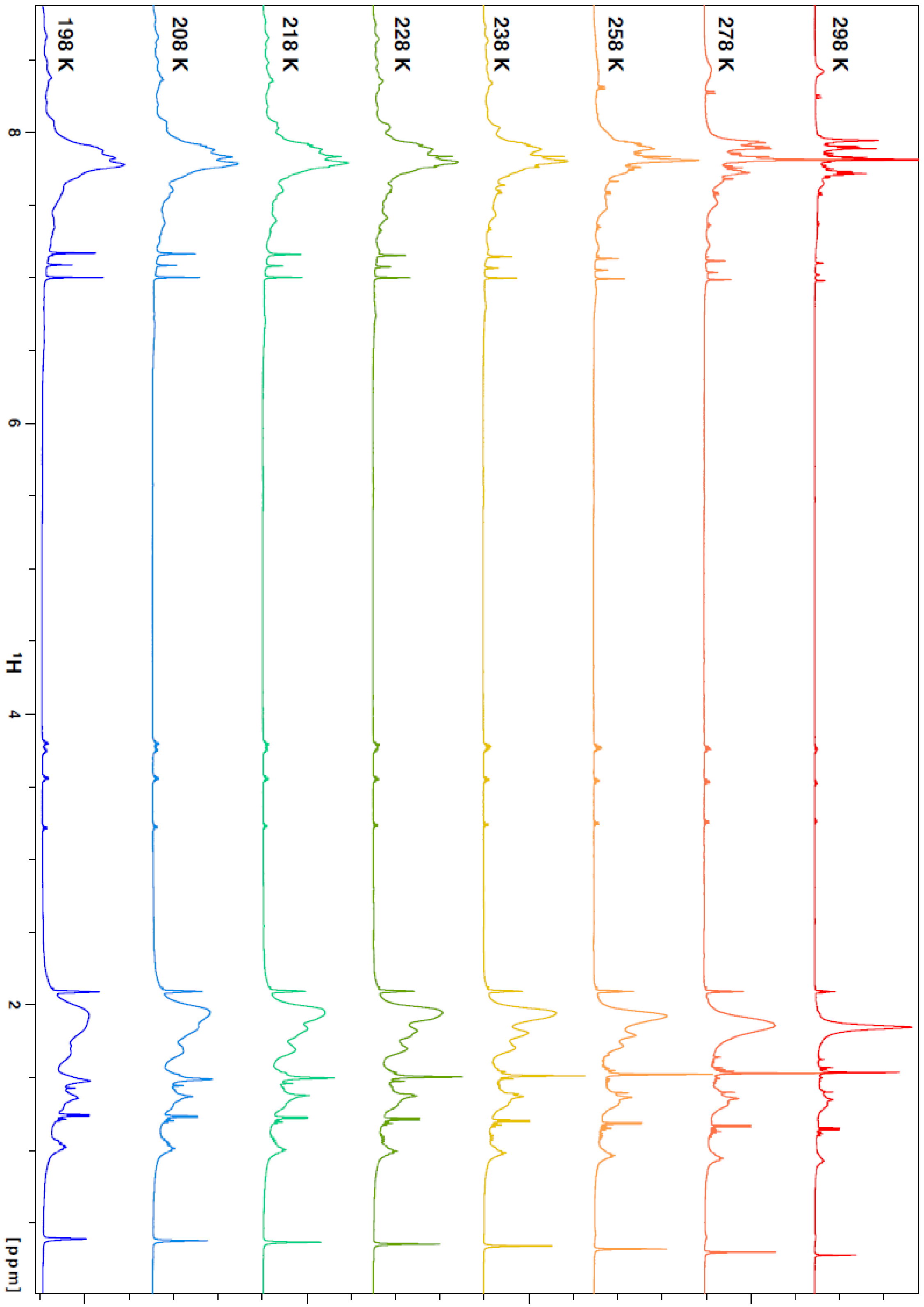

Figure S7. Variable temperature ^{1}H-NMR spectra of [Cr] in toluene-d_8 (600 MHz). The three resonances around 7 ppm originate from the residual solvent peak.

4.3 [Mn]$^+$

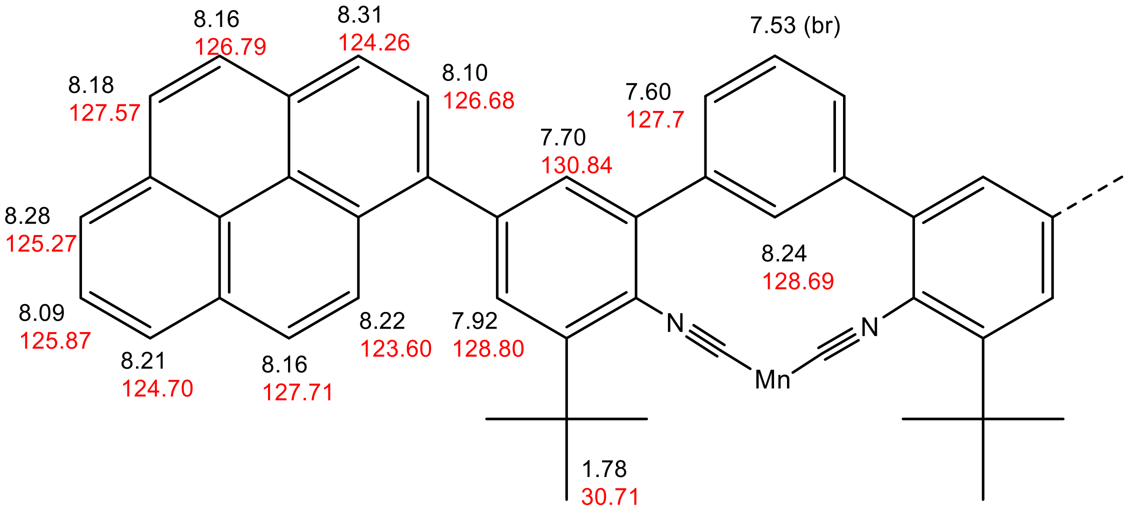

Figure S8. Assignment of the measured ^{1}H-NMR (black) and ^{13}C-NMR (red) resonances of [Mn]$^+$ in TCE-d_2 at 398 K (600 MHz). Due to the large linewidth of the proton signal at 7.53 ppm, no HSQC correlation to the adjacent carbon was observed. Likewise, the low signal to noise ratios in the recorded HMBC spectra prevented unambiguous assignment of all the quaternary carbons.

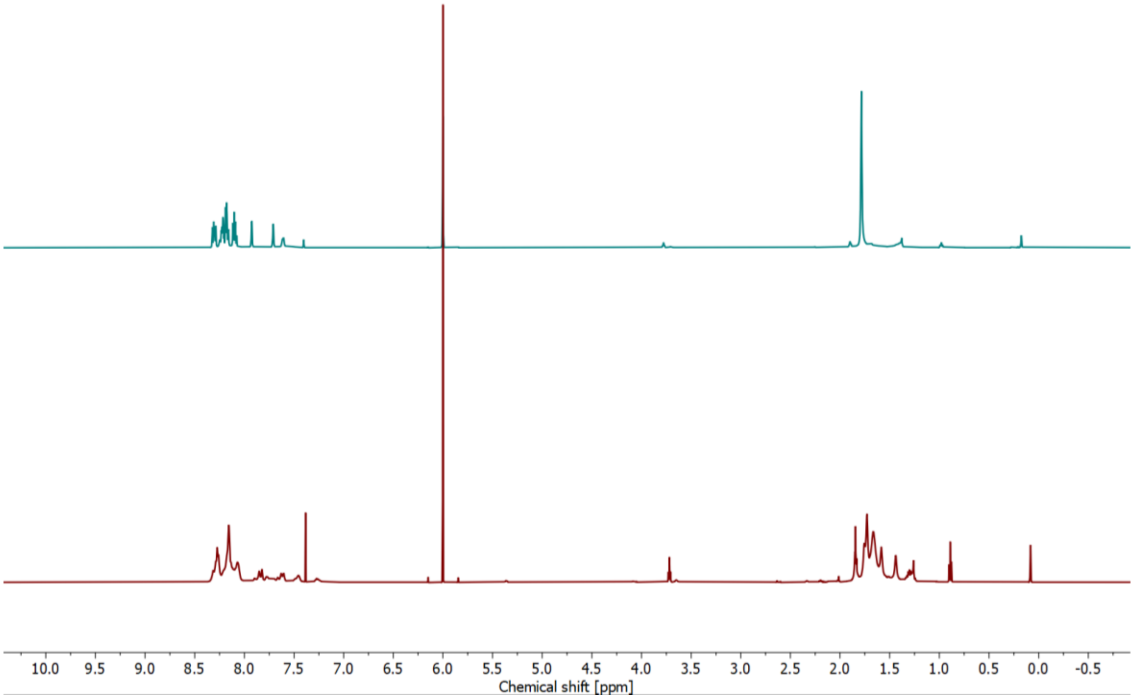

Figure S9. ^{1}H NMR spectrum of $[Mn]^{+}$ in TCE-d_2 (600 MHz) at 298 K (bottom) and 398 K (top). The solvent peak is seen at 6.0 ppm and the resonance at 1.78 ppm (398 K) originates from the *tert*-butyl groups.

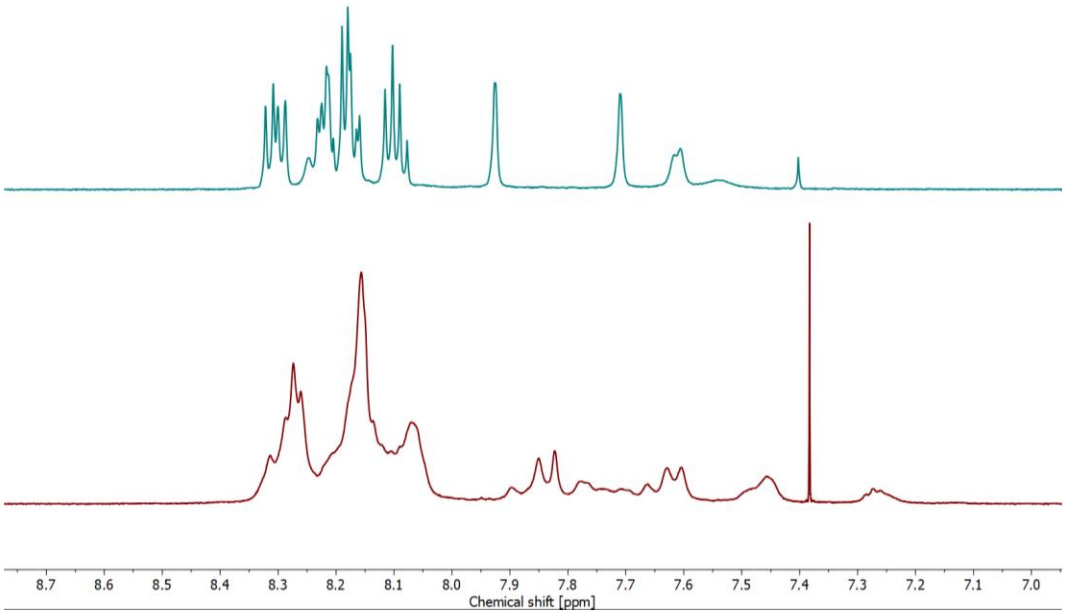

Figure S10. Aromatic region of the ^{1}H NMR spectrum of $[Mn]^{+}$ in TCE-d_2 (600 MHz) at 298 K (bottom) and 398 K (top).

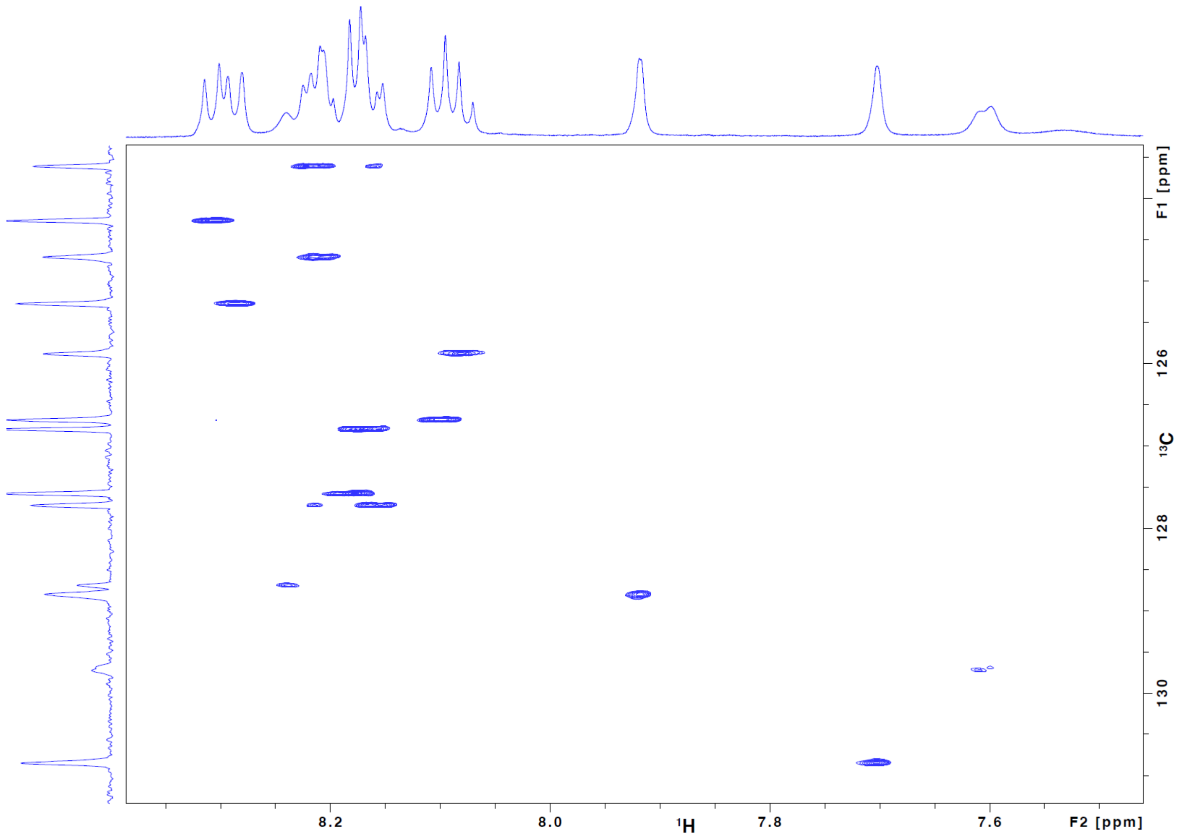

Figure S11. Aromatic region of the ^{1}H-^{13}C HSQC-NMR spectrum of $[Mn]^{+}$ in TCE-d_2 (600 MHz) at 398 K.

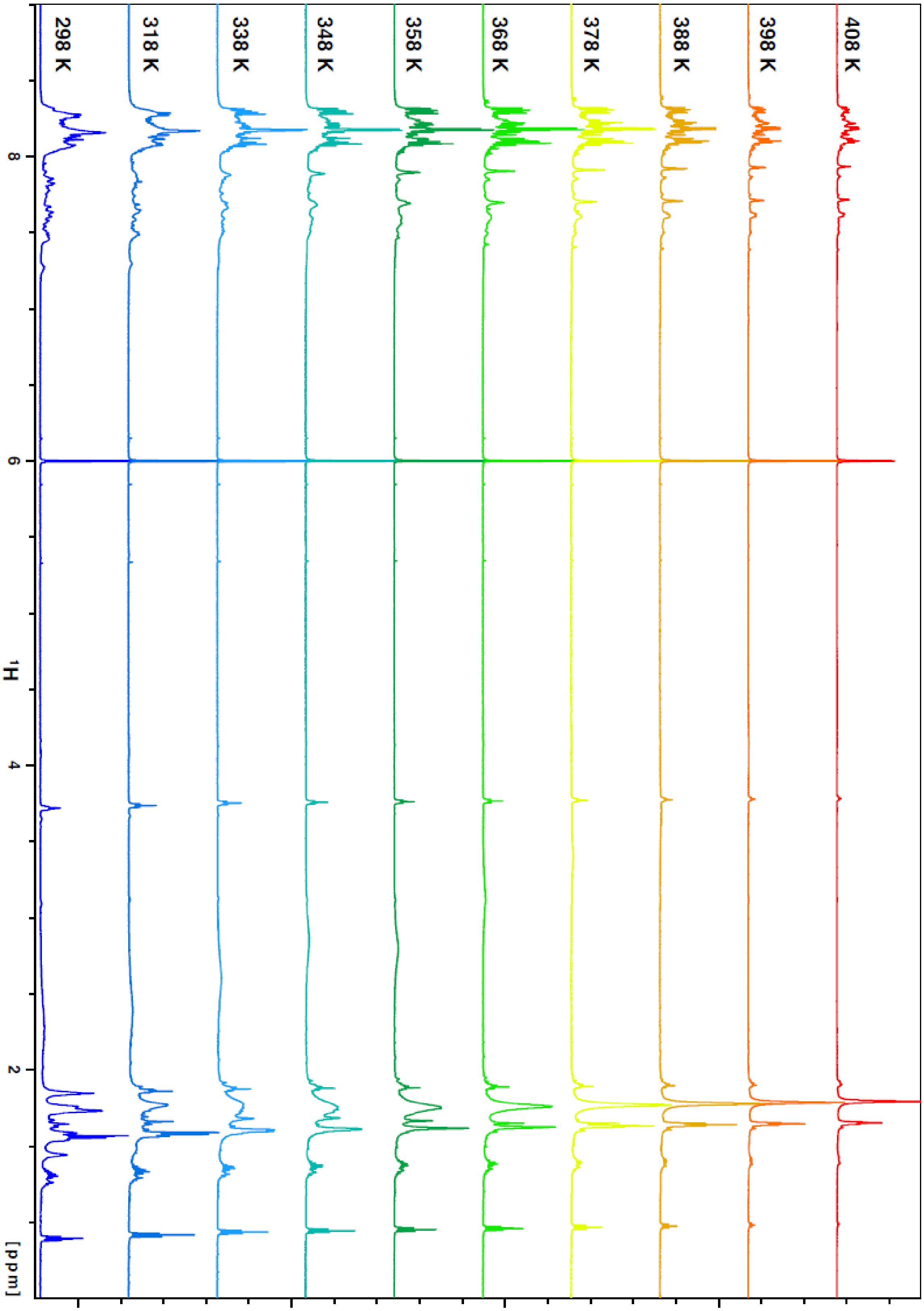

Figure S12. Variable temperature ^{1}H-NMR spectra of $[Mn]^+$ in TCE-d_2 (600 MHz). The solvent peak is seen at 6.0 ppm.

4.4 $[Fe]^{2+}$

Full NMR assignment of $[Fe]^{2+}$ was not possible due to the severe line broadening by dynamic conformational equilibria of the protons of the central benzene ring. Likewise, the low signal to noise ratios in the recorded HMBC spectra prevented unambiguous assignment of all the quaternary carbons.

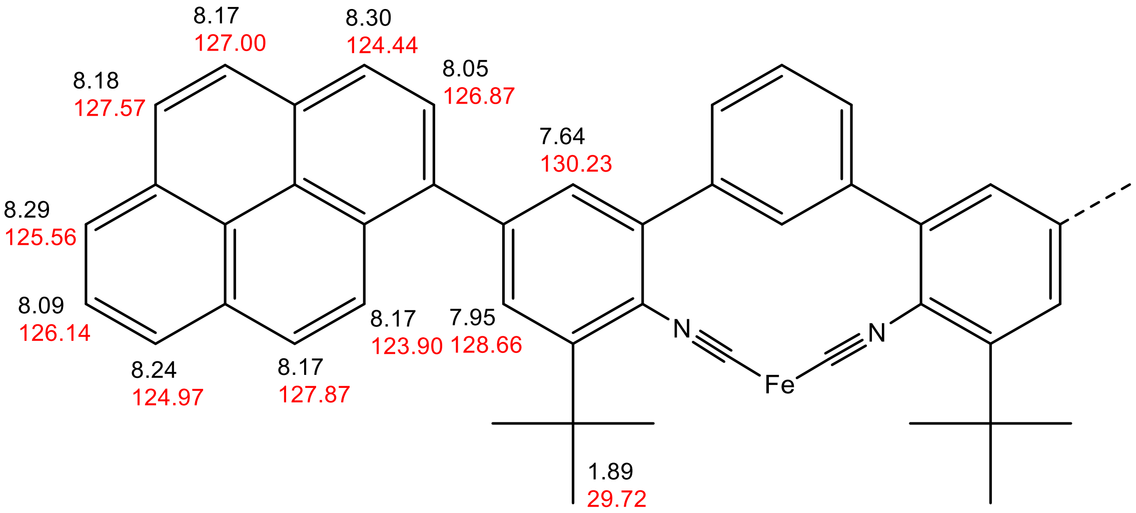

Figure S13. Assignment of the measured ^{1}H-NMR (black) and ^{13}C-NMR (red) resonances of $[Fe]^{2+}$ in TCE-d_2 at 338 K (600 MHz).

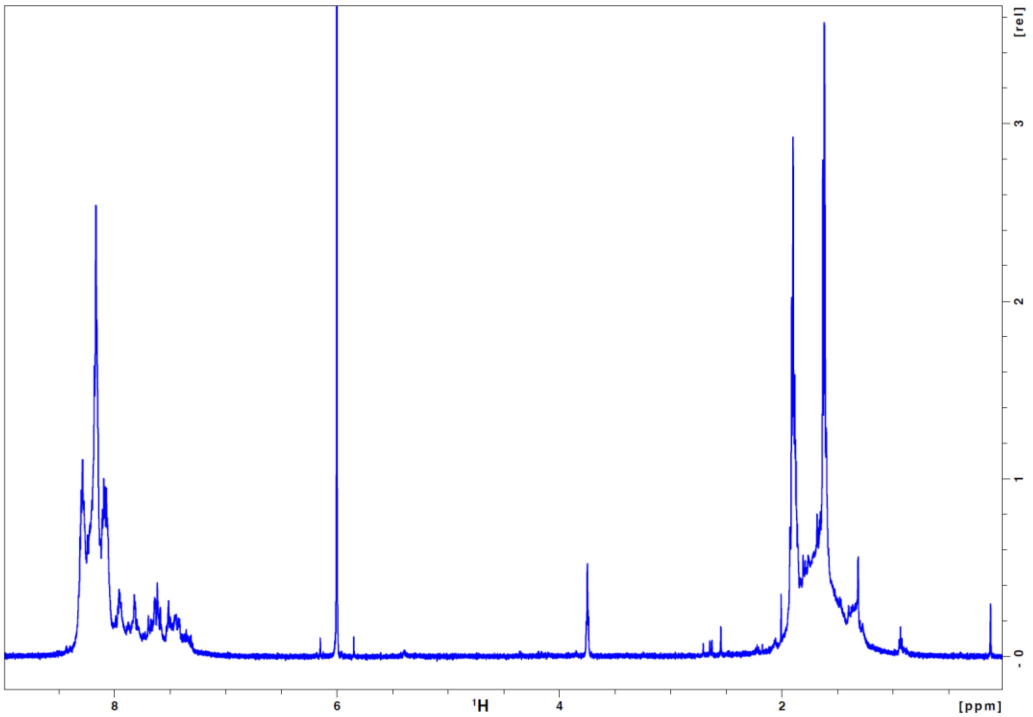

Figure S14. ^{1}H NMR spectrum of $[Fe]^{2+}$ in TCE-d_2 at 338 K (600 MHz). The solvent peak is seen at 6.0 ppm.

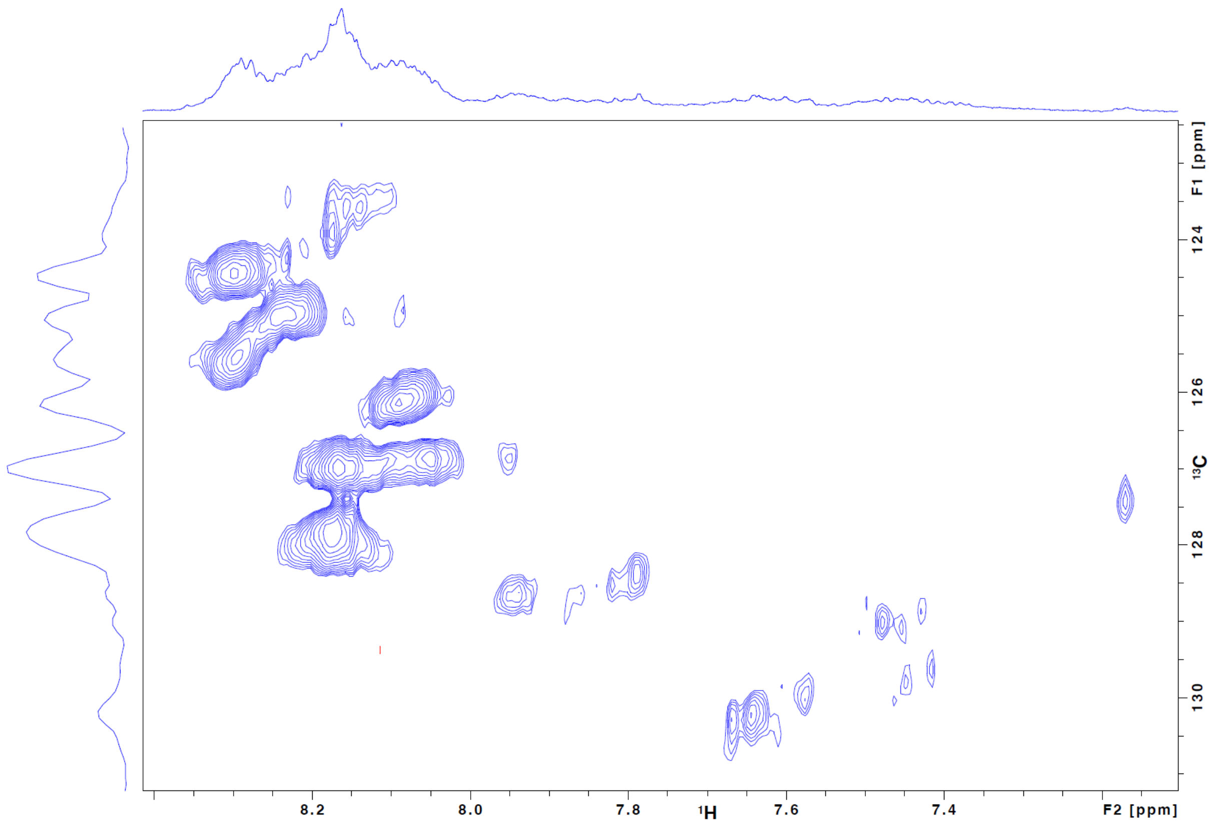

Figure S15. Aromatic region of the ^{1}H-^{13}C HSQC-NMR spectrum of $[Fe]^{2+}$ in TCE-d_2 at 338 K (600 MHz).

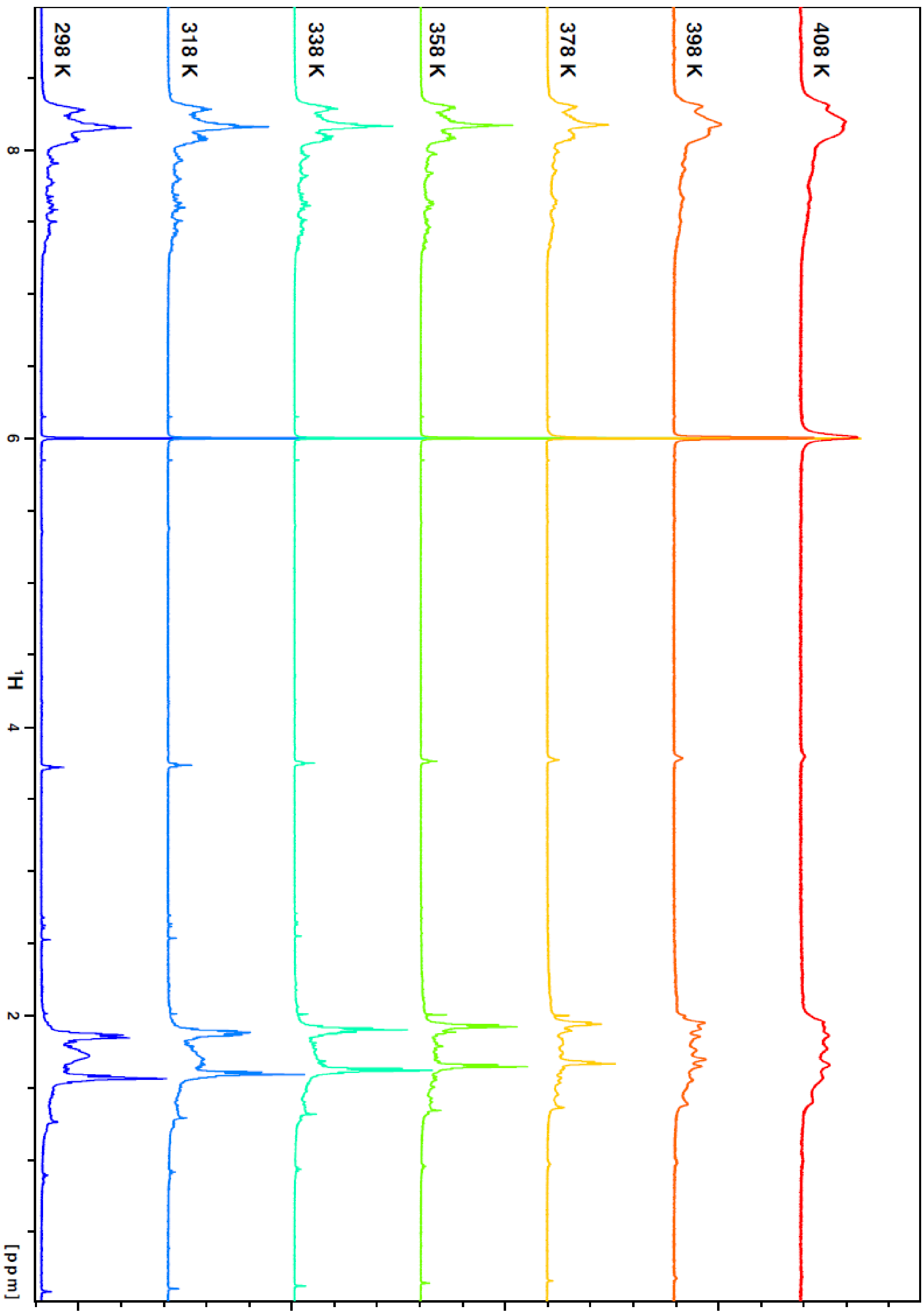

Figure S16. Variable temperature ^{1}H-NMR spectra of $[Fe]^{2+}$ in TCE-d_2 at 338 K (600 MHz). The solvent peak is seen at 6.0 ppm.

4.5 [$FeCl_2$]

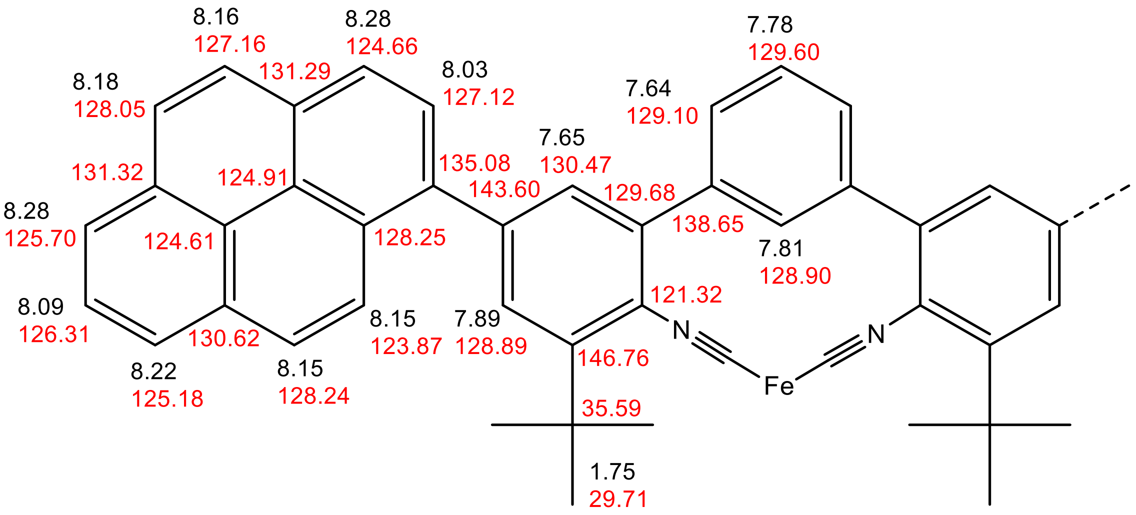

Figure S17. Assignment of the measured ^{1}H-NMR (black) and ^{13}C-NMR (red) resonances of [$FeCl_2$] in TCE-d_2 at 343 K (600 MHz).

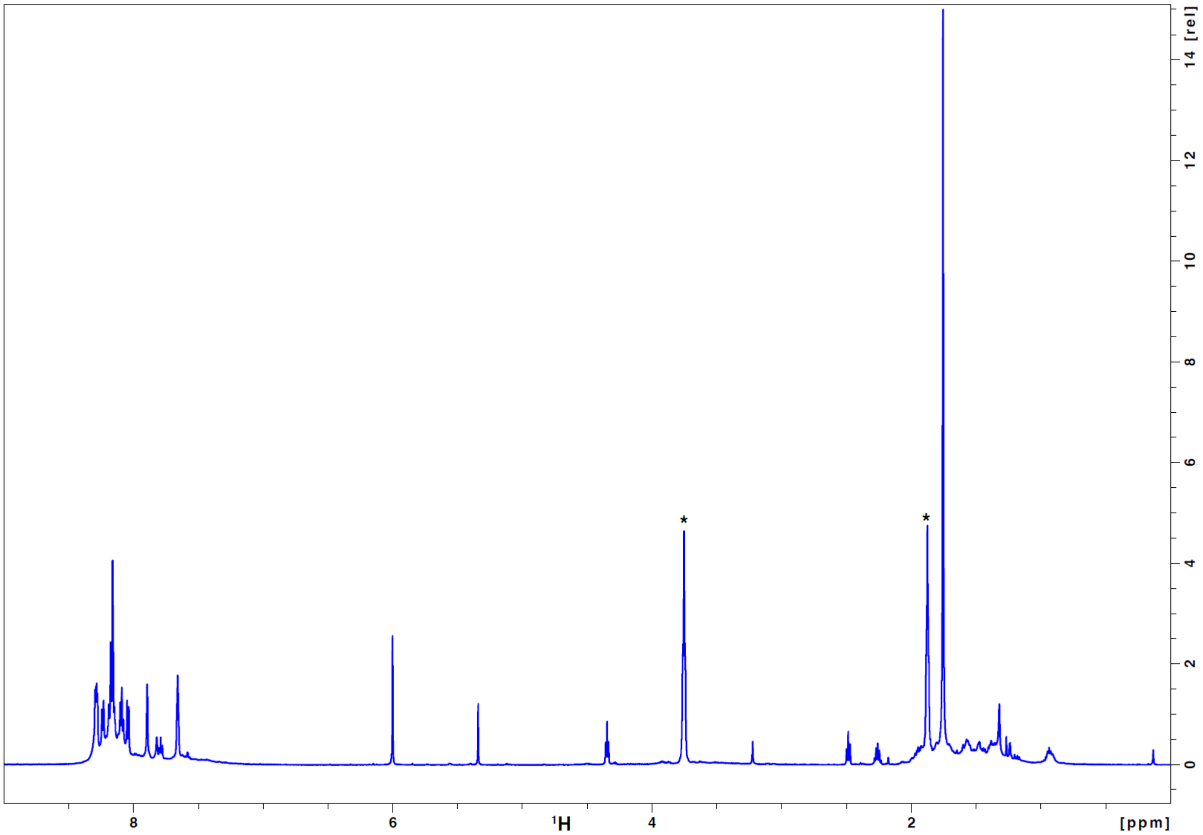

Figure S18. ^{1}H NMR spectrum of [$FeCl_2$] in TCE-d_2 at 338 K (600 MHz). Asterisks (*) denote traces of THF, and the solvent peak is seen at 6.0 ppm.

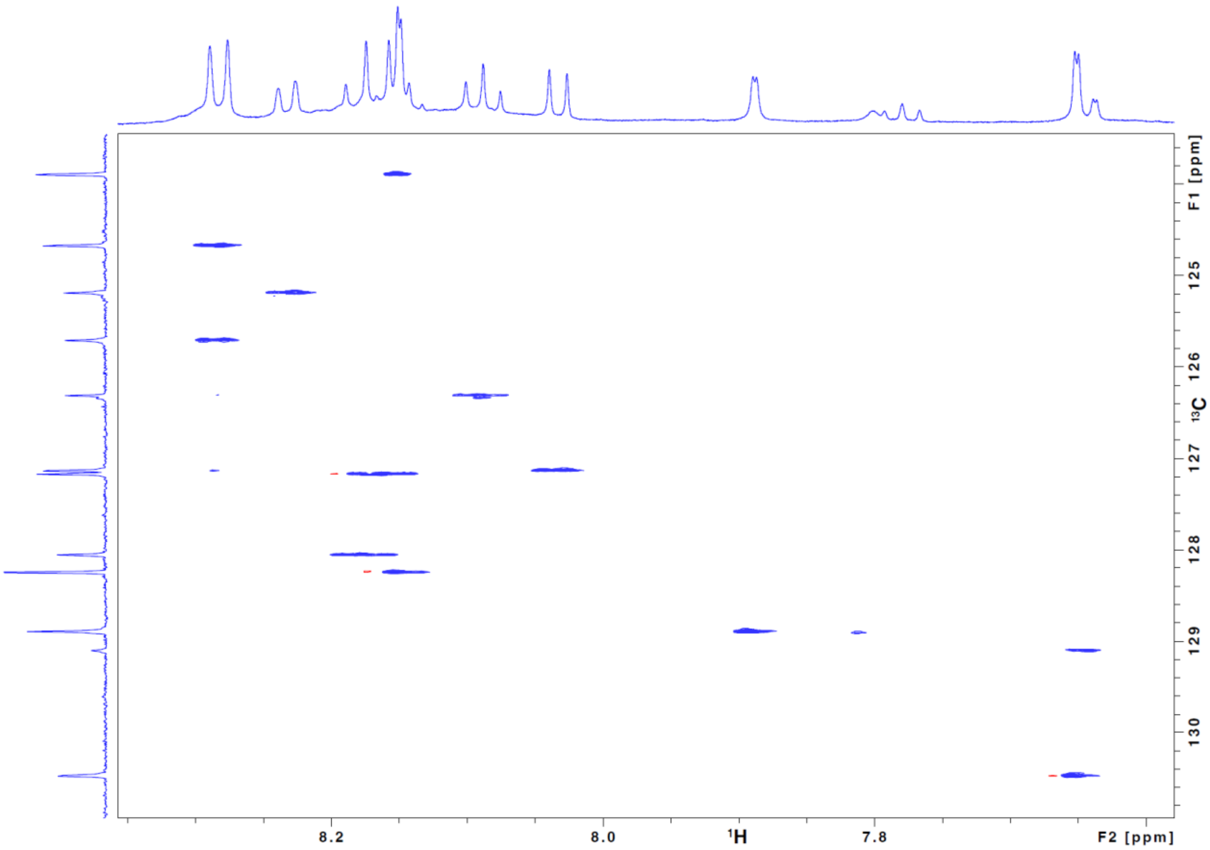

Figure S19. Aromatic region of the ^{1}H-^{13}C HSQC-NMR spectrum of [$FeCl_2$] in TCE-d_2 at 338 K (600 MHz).

Table S1. Overview of the relevant parameters of the *tert*-butyl signals for the Eyring analysis of the homoleptic $3d^6$-complexes investigated by variable temperature ^{1}H NMR spectroscopy, where $\Delta\nu$ is the maximum peak separation in the low-temperature (i.e. slow exchange) limit, T_c is the coalescence temperature and $\Delta G^{\ddagger}$ is the activation energy barrier.

**Compound**	$\Delta\nu$ [Hz]	T_c [K]	$\Delta G^{\ddagger}$ [kJ/mol]
[Cr]	114	278	55
$[Mn]^{+}$	68	338	69
$[Fe]^{2+}$	173	> 420	>83

5. Solid-State FTIR Spectra

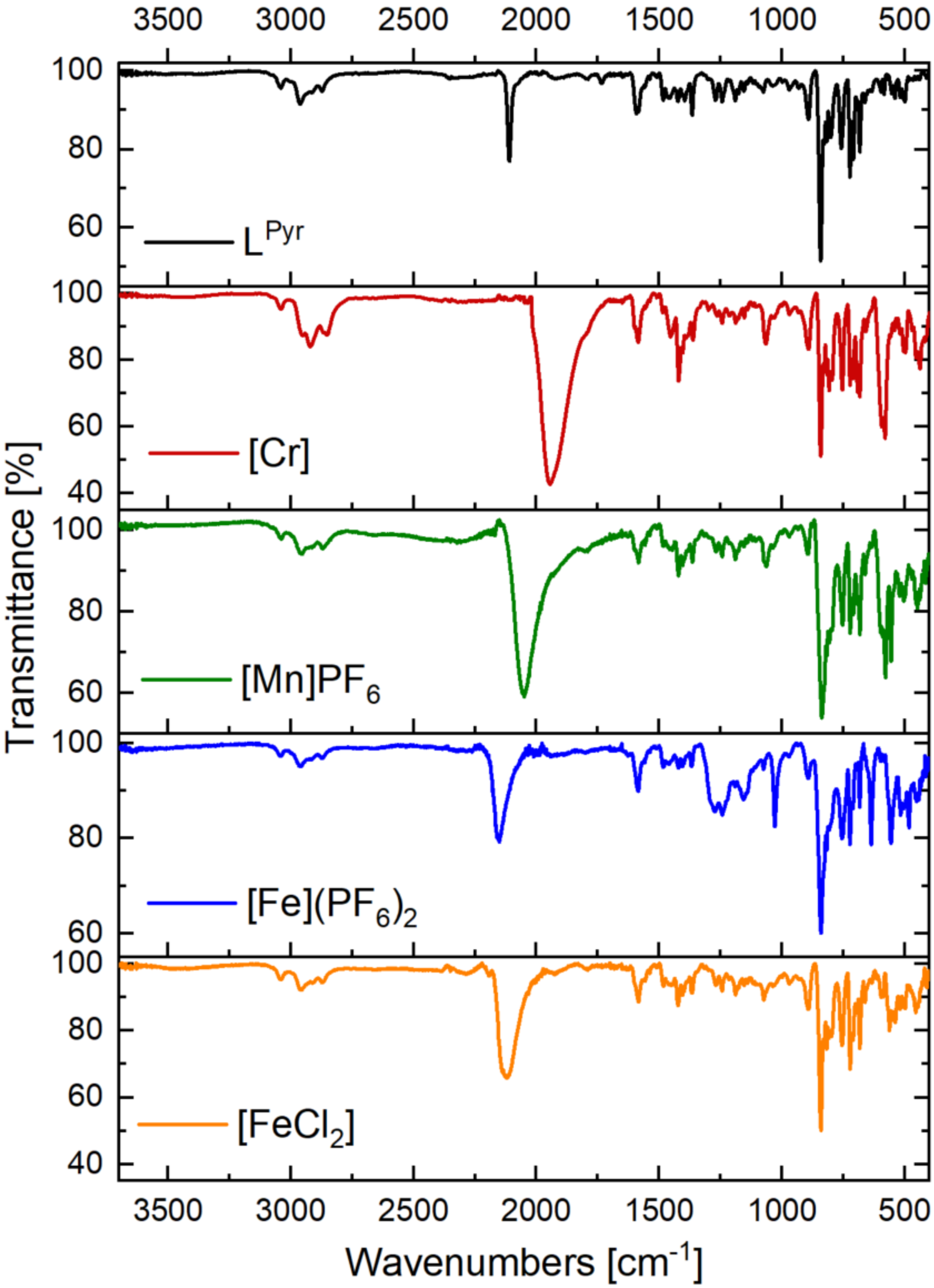

Figure S20. Solid-state IR spectra of L^{Pyr}, [Cr], [Mn]PF_6, [Fe]$(PF_6)_2$ and [$FeCl_2$].

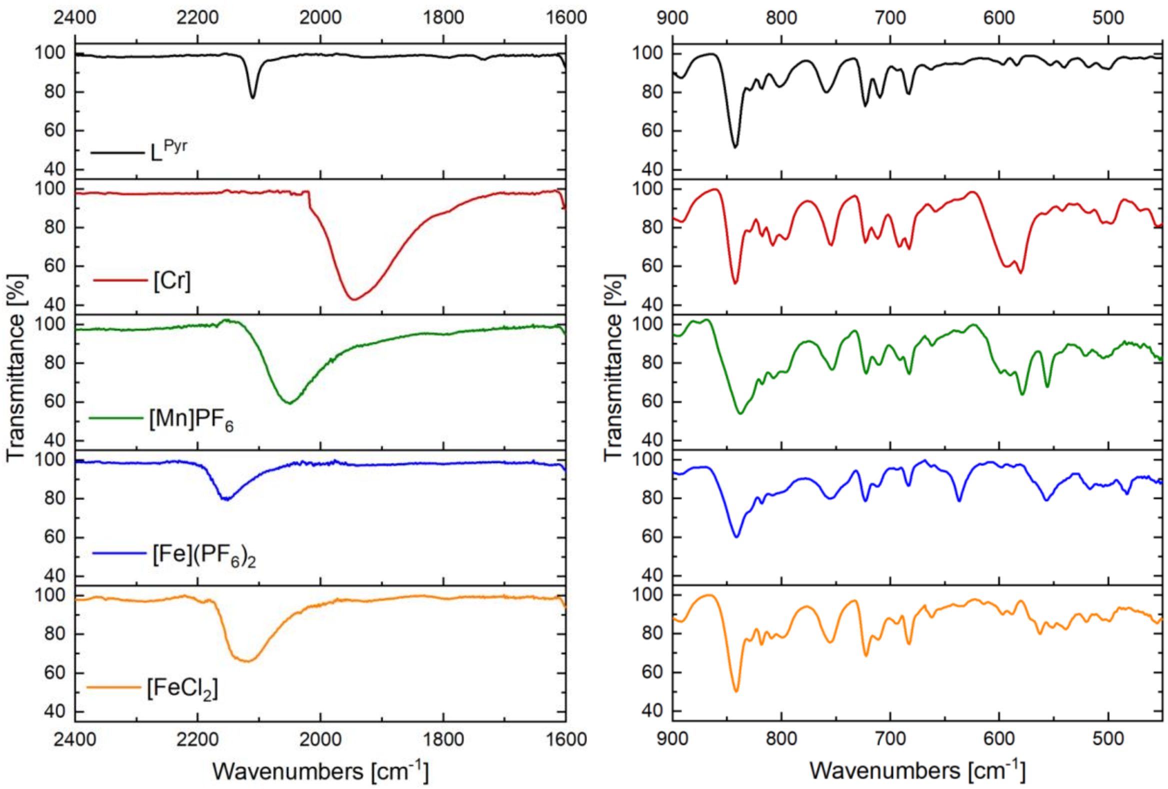

Figure S21. Solid-state IR spectra of L^{Pyr}, [Cr], [Mn]PF_6, [Fe]$(PF_6)_2$ and [$FeCl_2$]. Zoom of the C≡N stretching band region (left) and of the fingerprint region (right). The metal–C bending frequencies are found between 620 and 550 cm^{-1}.[7,8]

Table S2. Overview of C≡N stretching vibrations of L^{Pyr} and pyrene-decorated $3d^6$ complexes investigated in the present study.

**Compound**	**$\nu_{C≡N}$ [cm^{-1}]**
[Cr]	1945
[Mn]PF_6	2052
L^{pyr}	2111
[$FeCl_2$]	2125
[Fe]$(PF_6)_2$	2159

6. High-Resolution ESI Mass Spectra

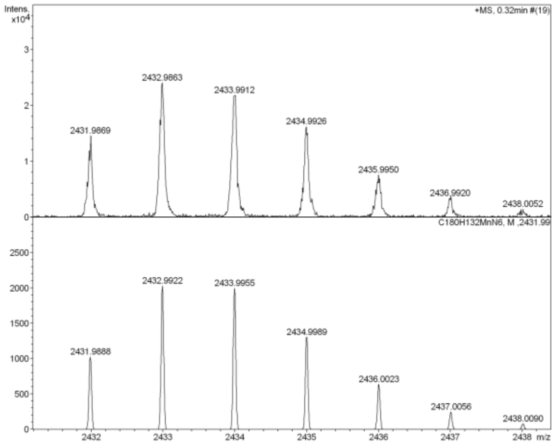

Figure S22. Top: HRMS-ESI (positive ions) mass spectrum of $[Mn]PF_6$. Bottom: Simulated mass spectrum of $[Mn]^+$.

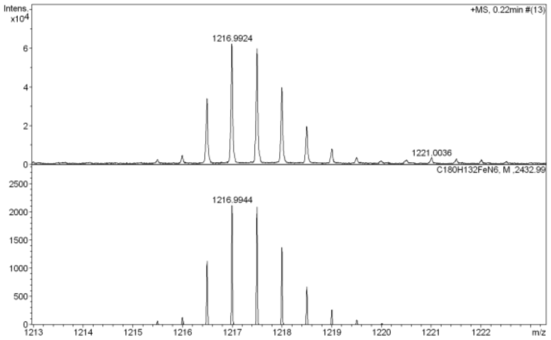

Figure S23. Top: HRMS-ESI (positive ions) mass spectrum of $[Fe](PF_6)_2$. Bottom: Simulated mass spectrum of $[Fe]^{2+}$.

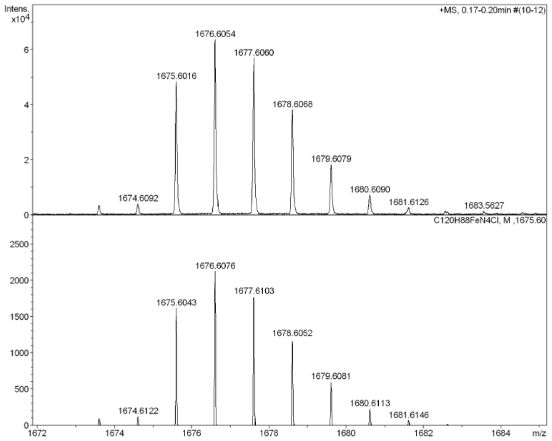

Figure S24. Top: HRMS-ESI (positive ions) mass spectrum of $[FeCl_2]$. Bottom: Simulated mass spectrum of the ion $\{[FeCl_2] – Cl\}^+$.

7. Cyclic Voltammetry

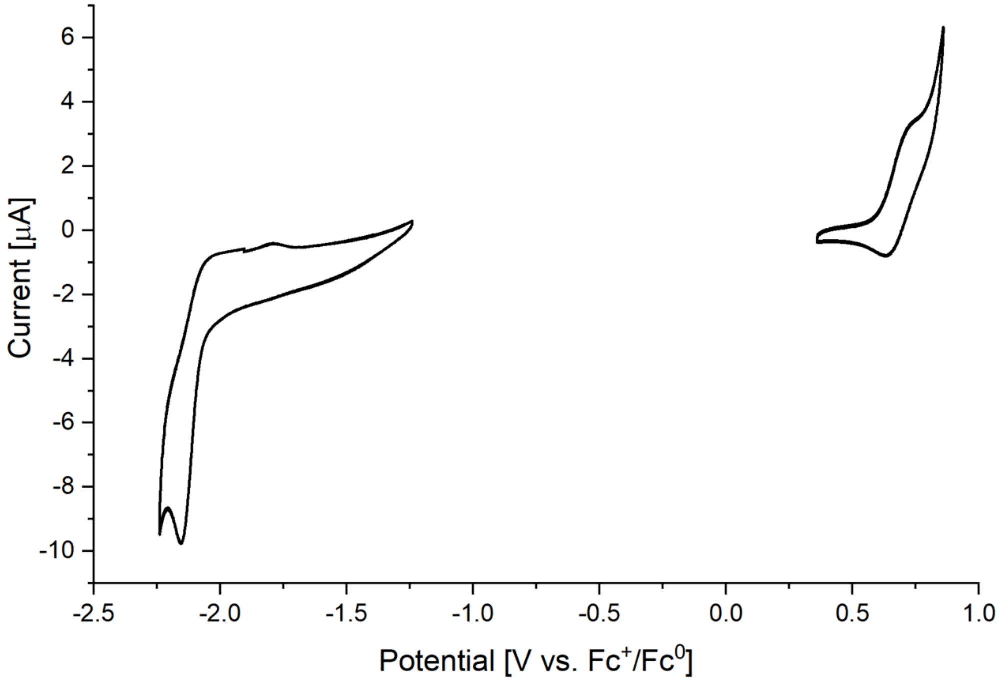

Figure S25. Cyclic voltammograms of 1 mM of $[Mn]^+$ in deaerated THF with 0.2 M of (^{n}Bu$_4$N)(PF$_6$) as supporting electrolyte. The scan rate was 100 mV/s.

L^{Pyr} in not stable under electrochemical conditions neither in THF nor in CH_2Cl_2. The Fe^{III}/Fe^{II} redox couples of $[Fe]^{2+}$ and $[FeCl_2]$ were not detectable in the electrochemical window of THF, and the iron(II) complexes were not stable at lower potentials than -1.5 V vs. Fc^+/Fc^0.

Table S3. Electrochemical parameters for [Cr], $[Mn]^+$ and pertinent manganese(I) isocyanide complexes.

**Compound**	**Solvent**	**$E_{½}$ of first metal oxidation vs. $Fc^{+/}Fc^0$ [V]**	**$E_{½}$ of ligand reduction vs. $Fc^{+/}Fc^0$ [V]**	**Reference**
**[Cr]**	THF	-0.68 (Cr^0/Cr^I)	-2.51	1
**$[Mn]^+$**	THF	0.68 (Mn^I/Mn^{II})	-2.12	This work
**$[Mn(L^{bi})_3]^+$**	CH_2Cl_2	0.67 (Mn^I/Mn^{II})	N/A	9
**$[Mn(CHdippPh^{OMe2})_6]^+$**	CH_2Cl_2	0.83 (Mn^I/Mn^{II})	N/A	7

8. Photophysical Characterization

8.1 L^{Pyr}

Emission Properties

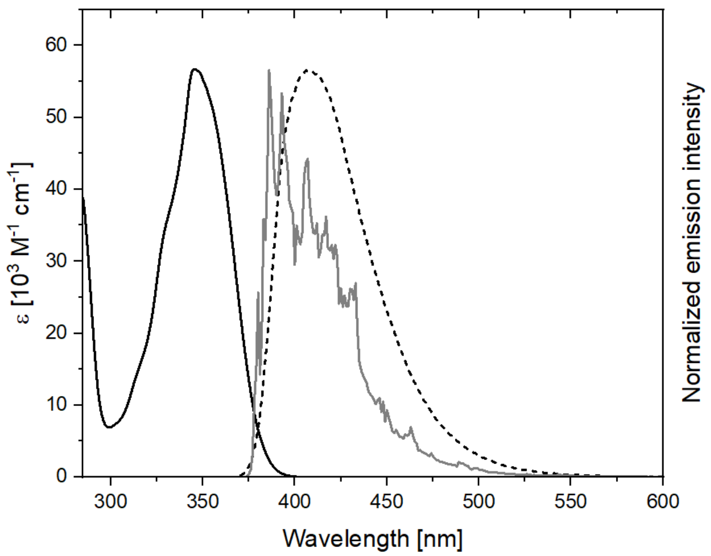

Figure S26. UV–Vis absorption (solid) and emission (dashed) spectra of L^{Pyr} in THF (black) at 20 °C and the emission spectrum of L^{Pyr} in a deaerated solution of 2-methyl-THF (gray) at 77 K.

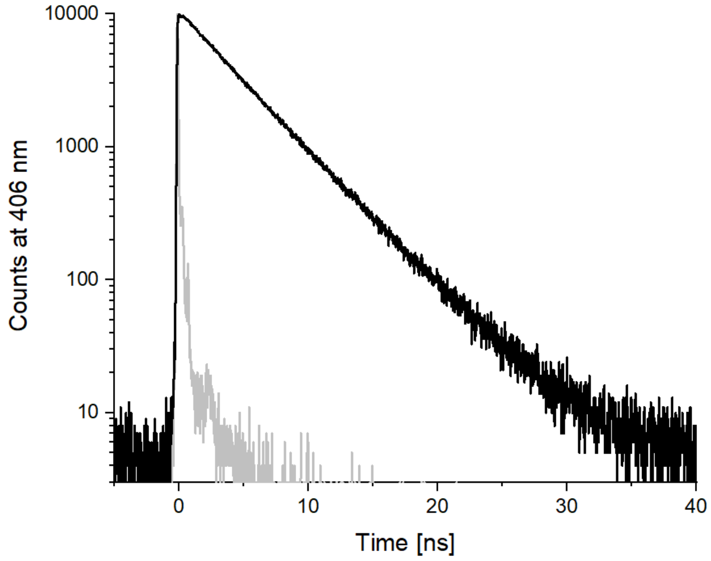

Figure S27. Fluorescence decay of L^{Pyr} in THF (black) at 20 °C as well the instrument response function (gray). Excitation occurred at 375 nm. The fluorescence lifetime of L^{Pyr} is 4.2 ns based on a single-exponential fit to the experimental data.

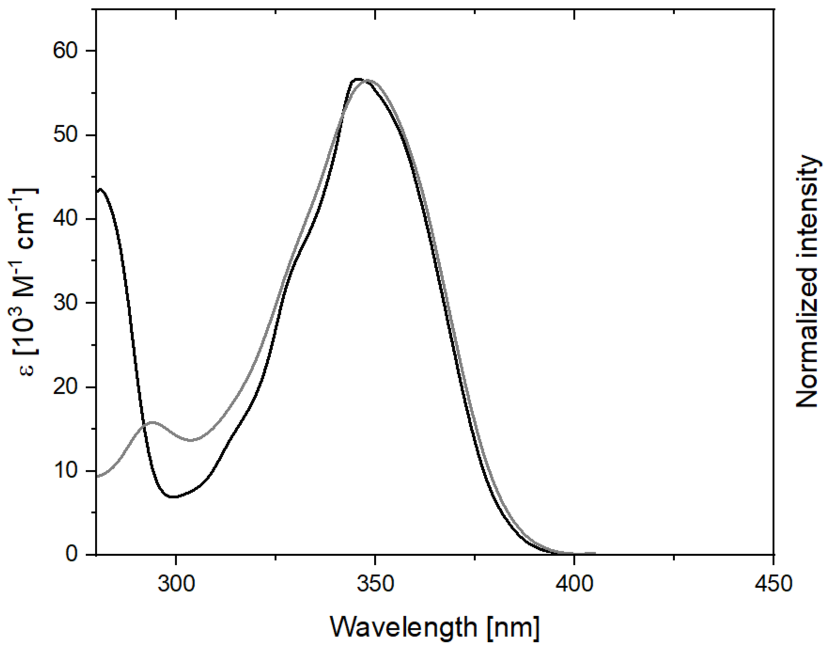

Figure S28. UV–Vis absorption (black) and excitation (gray, λ_{det} = 410 nm) spectra of L^{Pyr} in THF at 20 °C.

Table S4. Determination of the fluorescence quantum yield of L^{Pyr} in THF relative to DPA in cyclohexane at 20 °C. Excitation occurred at 350 nm.

<table>
<tr><th></th><th>L^{Pyr}</th><th>9,10-diphenylanthracene</th></tr>
<tr><td>UV-Vis absorption spectra</td><td>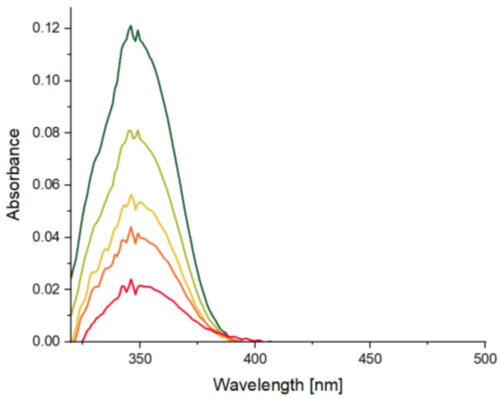
</td><td>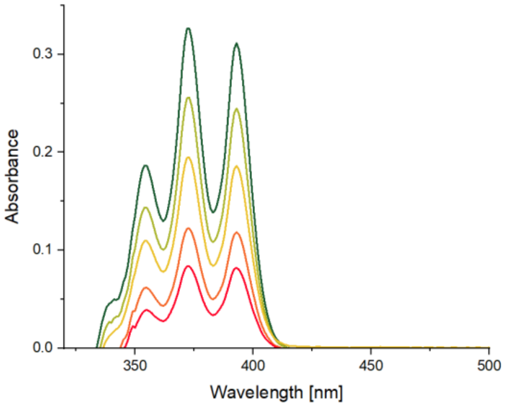
</td></tr>
<tr><td>Emission spectra</td><td>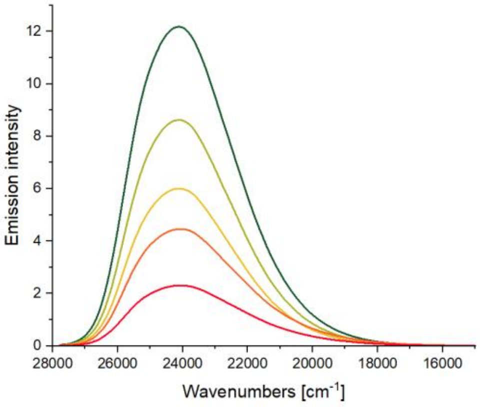
</td><td>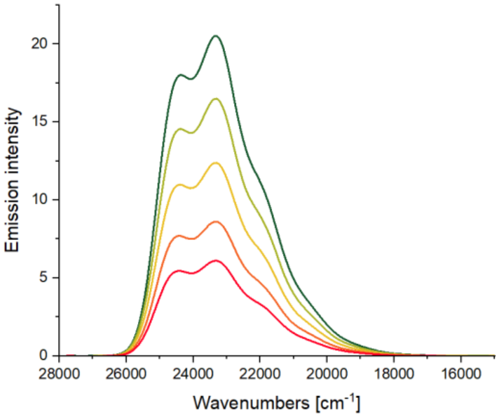
</td></tr>
<tr><td>Linear correlation</td><td>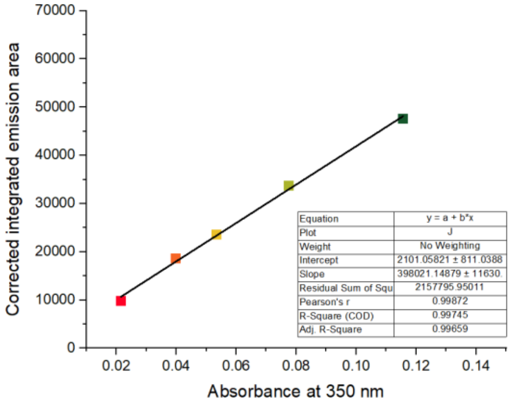
</td><td>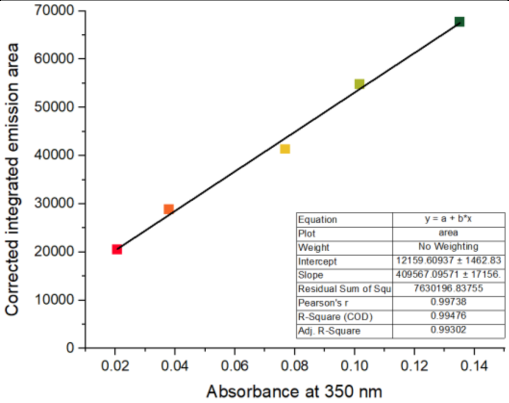
</td></tr>
</table>

Table S5. Selected photophysical properties of L^{Pyr} in THF at 20 °C.

$\lambda_{ABS,max}$ [nm]	$\lambda_{EM,max}$ [nm]	τ [ns]	φ
345	410	4.2	0.86

Excited-State Dynamic of L^{Pyr}

The excited state dynamics of L^{Pyr} in aerated THF were investigated at 350 nm and 370 nm (Figure S29) to enable direct comparison to the excited state dynamics of the complexes $[Mn]^+$ and $[Fe]^{2+}$ (See sections 8.3 and 8.4, respectively).

The following spectral features are identified for L^{Pyr}:

- Ground state bleach (GSB) at 365 nm
- Excited state absorptions (ESA) at 385, 515 and 650 nm
- Stimulated emission (SE) at 430 nm

The stimulated emission from L^{Pyr} is, as expected, slightly redshifted to the spontaneous emission (band maxima at 430 vs. 410 nm, respectively).

The ESA at 650 nm and the SE at 430 nm are observable in the accessible spectral window for both investigated excitation wavelengths. Monitoring the evolution of these two features over time (Figure S30) makes it clear that excitation of L^{Pyr} at 350 and 370 nm results in population of higher $S_n \leftarrow S_0$ transitions, thus a buildup of the S_1 population occurs before a decay of the ESA at 650 nm alongside observation of SE.

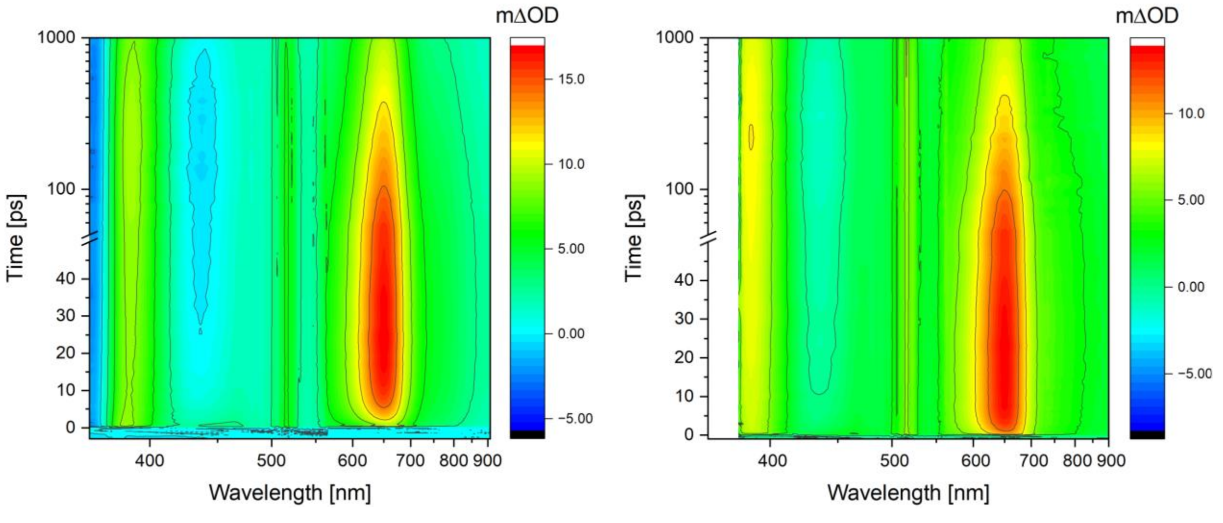

Figure S29. Excited state dynamics of L^{Pyr} in aearted THF recorded with excitation at 350 (right) and 370 nm (left).

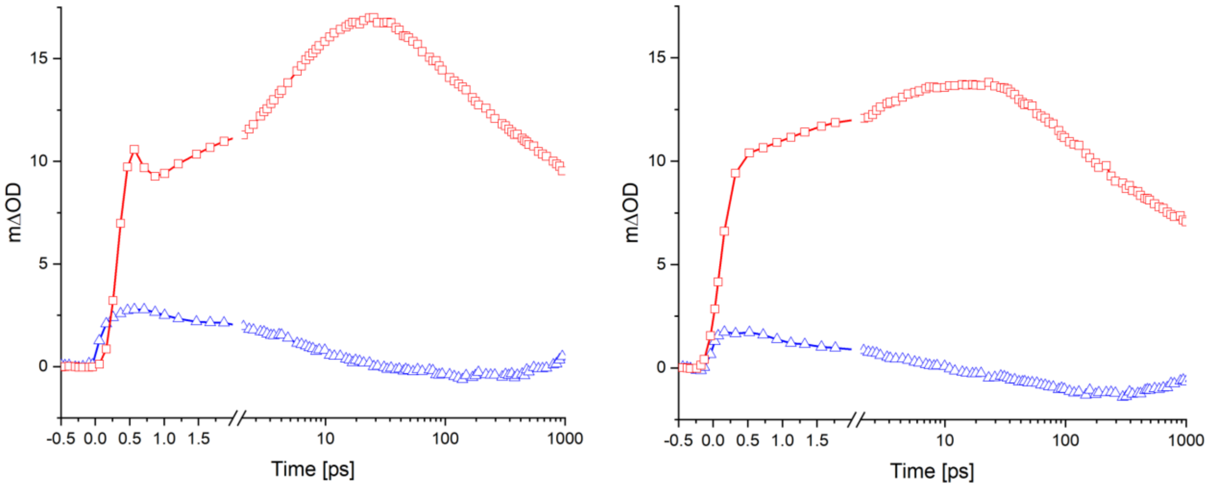

(a) (b)

Figure S30. Temporal evolution of the ESA at 650 nm (red) and SE at 430 (blue) for L^{Pyr} in aerated THF at 20 °C. Excitation occurred at 350 nm (a) or 370 nm (b).

Global Fit Analysis

The transient absorption data of L^{Pyr} (Figure S29) was modelled with a sequential deactivation pathway taking into account the buildup of the S_1 state from higher S_n states:

$$\mathbf{S_n} \xrightarrow{k_{L,1}} \textbf{“hot” } \mathbf{S_1} \xrightarrow{k_{L,2}} \mathbf{S_1} \xrightarrow{k_{L,3}} \mathbf{S_0}$$

where $k_{L,1}$ represents the rate constant of internal conversion from S_n to higher vibrational states of S_1, $k_{L,2}$ is the vibrational relaxation of the S_1 excited state to the lowest vibrational state of S_1, and $k_{L,3}$ is the rate constant of the relaxation back to the ground state S_0. Table S6 summarizes the rate constants determined for the two investigated excitation wavelengths and the corresponding lifetimes.

The lifetime of the spontaneous emission was determined to 4.2 ns in THF (Figure S27), which is in good agreement with the lifetime of 4.7 nm determined in the global fit analysis considering the limited number of data points at longer time scales.

Table S6. Overview of rate constants and the corresponding lifetimes for the relaxation process of L^{Pyr}.

Excitation wavelength	$k_{L,1}$ [ps^{-1}]	$k_{L,2}$ [ps^{-1}]	$k_{L,3}$ [ps^{-1}]	$\tau_{L,1}$ [ps]	$\tau_{L,2}$ [ps]	$\tau_{L,3}$ [ns]
350	0.1760	0.008000	0.0002097	5.68	125	4.77
370	0.7462	0.006960	0.0002113	1.34	144	4.73

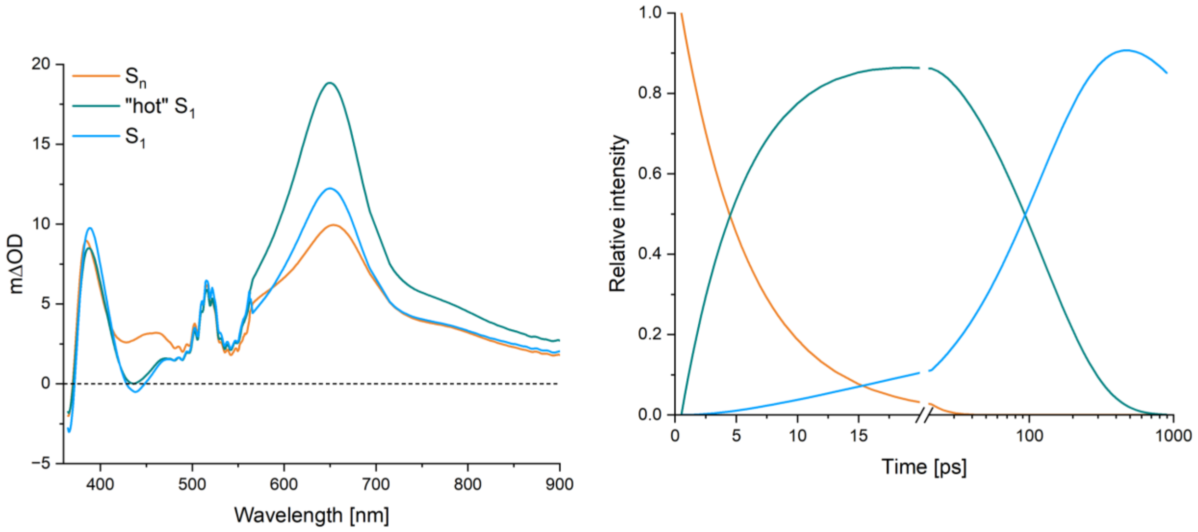

(a) (b)

Figure S31. (a) Species associated spectra and (b) the corresponding concentration profiles related to the excited state dynamics of L^{Pyr} in aerated THF at 20 °C. Excitation occurred at 350 nm.

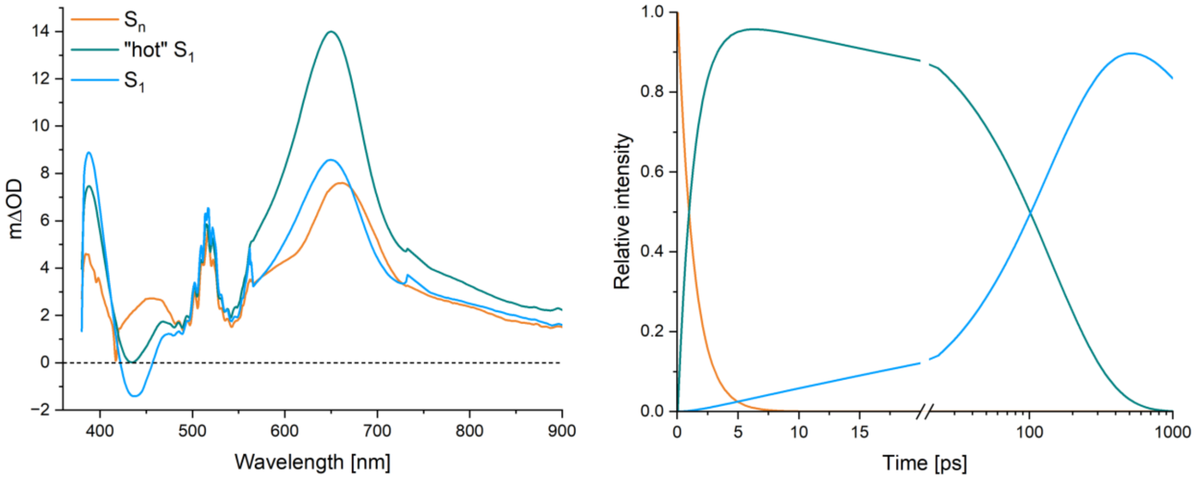

(a) (b)

Figure S32. (a) Species associated spectra and (b) the corresponding concentration profiles related to the excited state dynamics of L^{Pyr} in aerated THF at 20 °C. Excitation occurred at 370 nm.

Triplet Absorption Spectrum of L^{Pyr}

The triplet absorption spectrum of L^{Pyr} was measured after triplet-triplet energy transfer (TTET) from *fac*-[Ir(ppy)$_3$]$_3$ (ppy = 2-phenylpyridine) as the photosensitizer (Figure S33). The ^{1}MLCT band of the iridium complex was selectively excited at 450 nm. Subsequently, rapid and efficient intersystem crossing (ISC) generates the long-lived ^{3}MLCT excited state at 2.4 eV above the electronic ground state,[10] from where the lowest triplet excited state of L^{Pyr} is populated through bimolecular TTET.

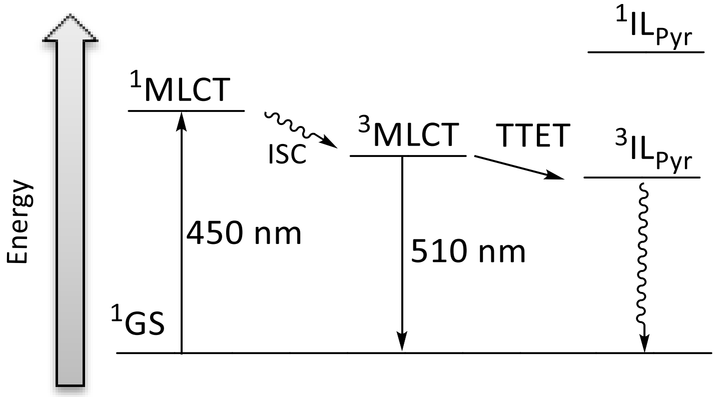

Figure S33. Jablonski diagram depicting the energy levels involved in the TTET between the photosensitizer *fac*-[Ir(ppy)$_3$] and the free ligand L^{Pyr}.

The photoluminescence of *fac*-[Ir(ppy)$_3$] monitored at 510 nm is quenched significantly upon addition of 1 mM of L^{Pyr} (Figure S34). A transient absorption spectrum was recorded after essentially complete photoluminescence decay of *fac*-[Ir(ppy)$_3$] with a delay time of 2 μs, hereby yielding the transient absorption spectrum of the lowest triplet excited state of L^{Pyr}, i.e. ^{3}IL$_{Pyr}$ (Figure S35).

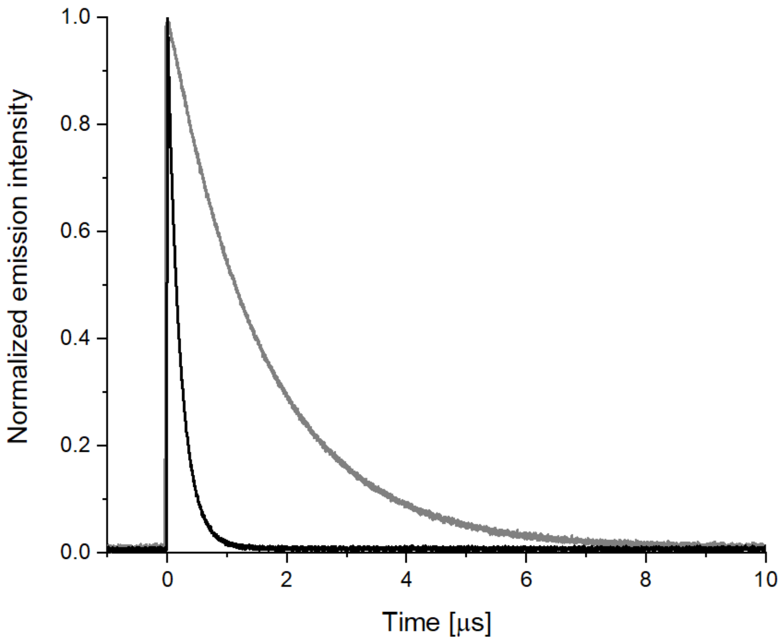

Figure S34. Photoluminescence decay at 510 nm of *fac*-[Ir(ppy)$_3$] (33 μM) in the absence (gray) and presence (black) of 1 mM L^{Pyr} in deaerated THF at 20 °C. Excitation occurred at 450 nm. The luminescence lifetimes in the absence and presence of L^{Pyr} are 1.58 μs and 217 ns, respectively.

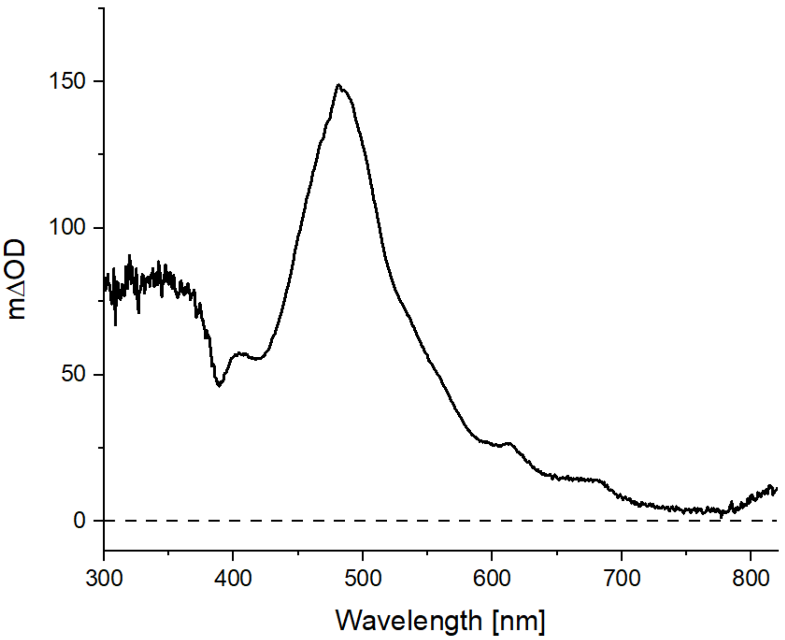

Figure S35. Triplet absorption spectrum of L^{Pyr} in deaerated THF 20 °C. The spectrum was obtained following TTET from *fac*-[Ir(ppy)$_3$] (see Figure S33). The spectrum was time-integrated for 200 ns with a delay time of 2 μs. Selective excitation of *fac*-[Ir(ppy)$_3$] occurred at 450 nm.

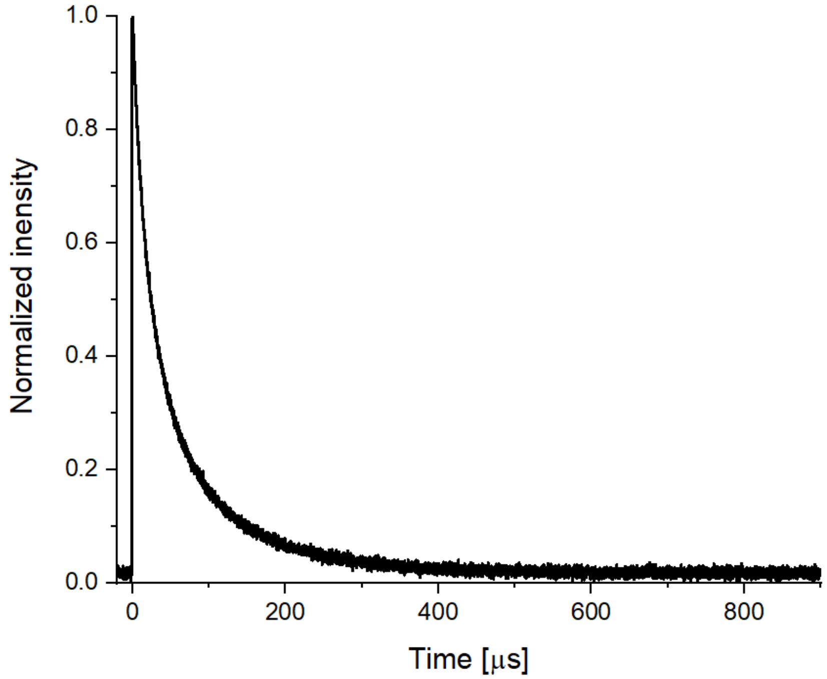

Figure S36. Absorption decay at 492 nm of the triplet state of L^{Pyr} in deaerated THF at 20 °C. The experimental decay was fitted with a biexponential function, yielding the following lifetimes and relative weighting factors: 19.4 μs (56%), 90.7 μs (44%), resulting in a weighted average lifetime of 49.5 μs for $^{3}L^{Pyr}$.

8.2 [Cr]

UV–Vis Absorption Spectroscopy

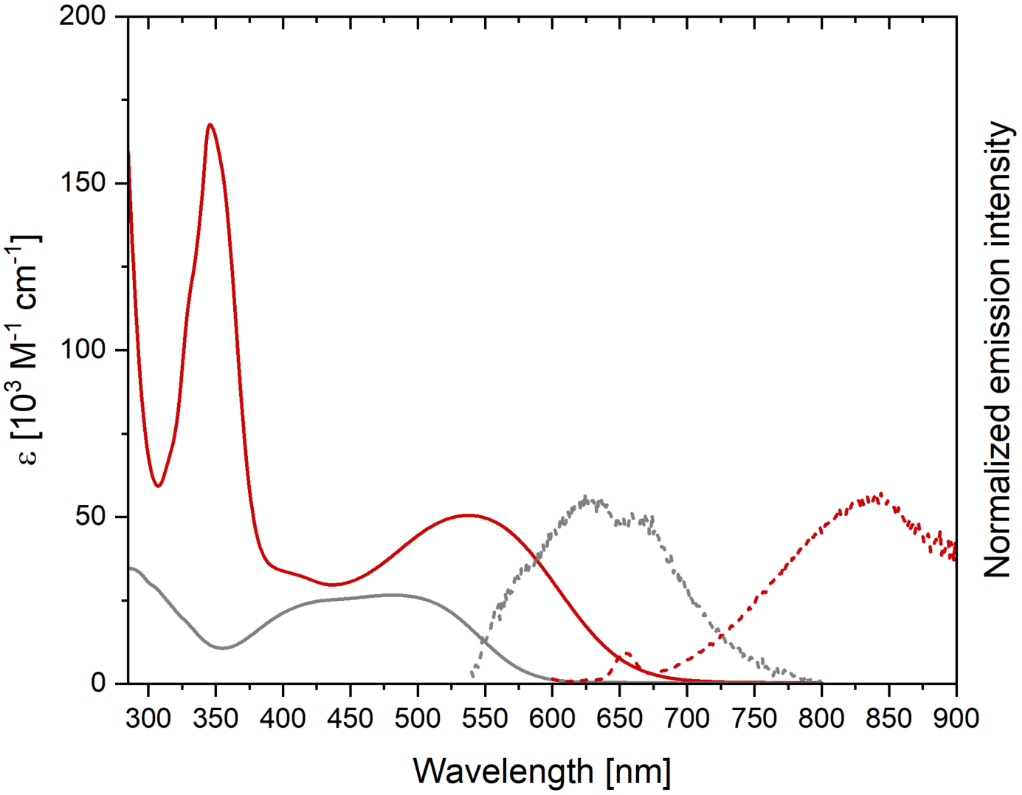

Figure S37. UV–Vis absorption (solid) and emission (dashed) spectra of [Cr] (red) and $[Cr(L^{tBu})_3]$[11] (gray) in deaerated solutions of THF at 20 °C. Excitation occurred at 550 and 473 nm, respectively. The ^{1}MLCT absorption band maximum has redshifted from 480 nm to 540 nm, which suggests that a π* stabilization of ca. 0.3 eV has occurred. The Cr^{I}/Cr^{0} redox couples for [Cr] and $[Cr(L^{tBu})_3]$ are -0.68 and -0.83 V vs Fc^{+}/Fc, respectively, hence some π* orbital stabilization indeed seems to occur upon pyrene attachment, though it seems to be closer to 0.15 eV rather than 0.3 eV.

Luminescence Quantum Yield Determination

The luminescence quantum yields of [Cr] in deaerated solutions of cyclohexane and toluene at 20 °C have previously been determined to 1.04% and 0.66%, respectively.[1] Determination of the absolute luminescence quantum yield of [Cr] in deaerated THF is unreliable, as our Fluorolog 322-instrument has a detection limit near 850 nm, thus not covering the entire emission band of [Cr] in THF, and the sensitivity of our FP-8600 spectrophotometer is insufficient. However, in a comparative study (Figure S38) the order of magnitude of the luminescence quantum yield of [Cr] in deaerated THF at 20 °C was estimated to 10^{-4}, using $[Os(bpy)_3](PF_6)_2$ in deaerated acetonitrile as a reference (φ = 0.46%).[12]

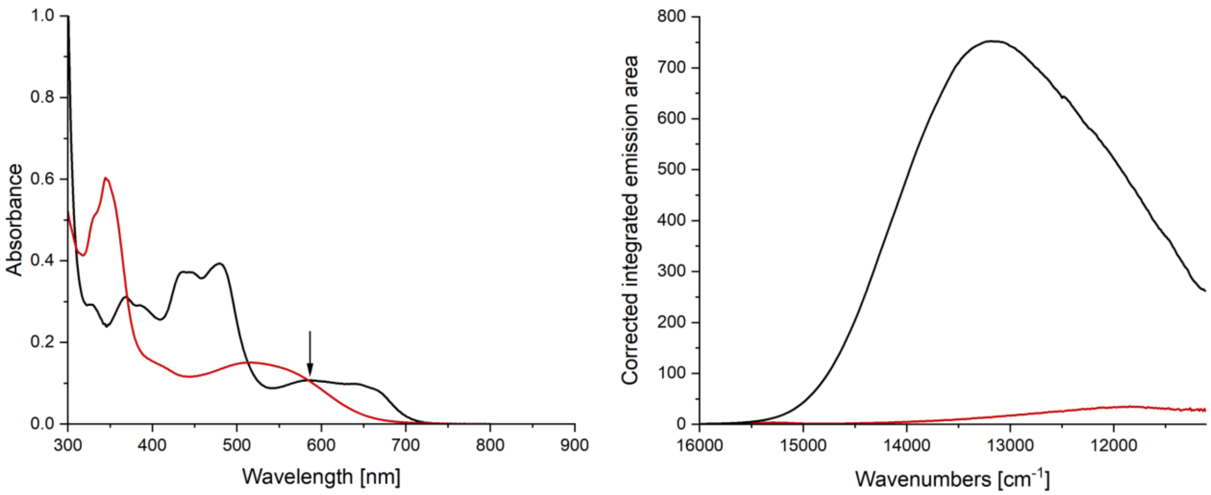

(a) (b)

Figure S38. Comparative luminescence measurements for estimation of the luminescence quantum yield of [Cr] (red) in deaerated THF at 20 °C using $[Os(bpy)_3]^{2+}$ (black) in deaerated acetonitrile as a reference. UV–Vis absorption (a) and emission (b) spectra of [Cr] and $[Os(bpy)_3]^{2+}$, adjusted to an identical optical density at the excitation wavelength of 580 nm (arrow in (a)) to ensure the absorption of an equal amount of photons by both samples. The emission spectra were recorded under strictly identical conditions, to allow for direct comparison of the integrated emission intensities, which were determined with Gaussian fitting. The y-axis of the emission spectra was corrected according to eq. S2 prior to the integration.

Table S7. Selected photophysical parameters of the ^{3}MLCT excited state in [Cr]

Solvent	τ [ns]	φ [%]	k_r [s^{-1}]	k_{nr} [s^{-1}]
Cyclohexane	47	1.04	2×10^5	2×10^7
Toluene	24	0.66	3×10^5	4×10^7
THF	1.1	0.01	0.9×10^5	90×10^7

Excited State Dynamics

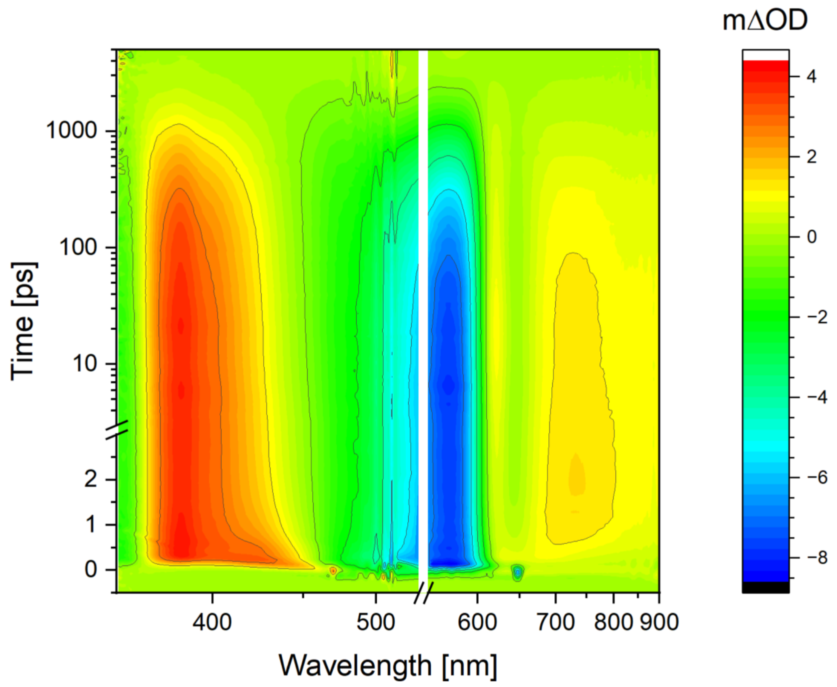

Figure S39. 2D contour plot showing the transient absorption spectra of [Cr] in deaerated THF at 20 °C as a function of delay time between the pump and probe. Excitation occurred at 550 nm.

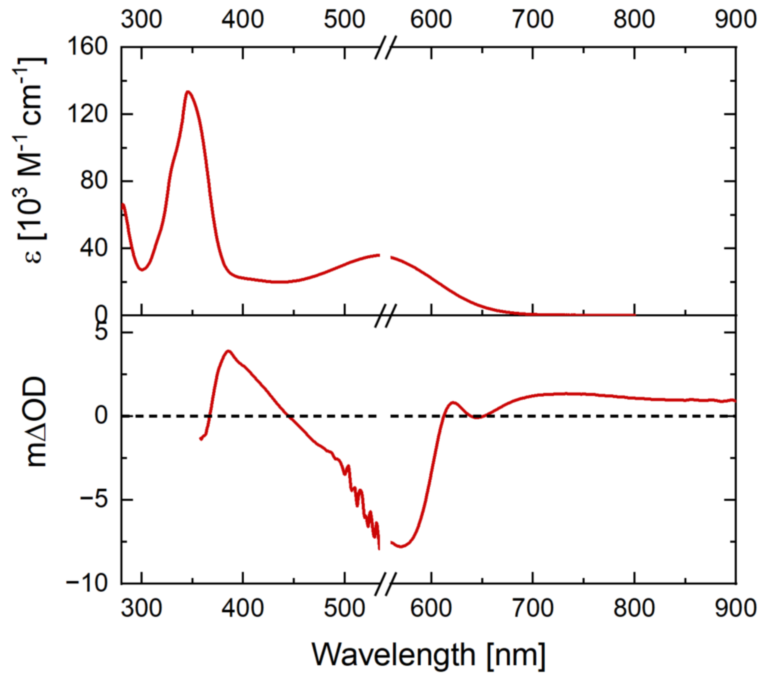

Figure S40. UV–Vis absorption (top) and transient absorption (bottom) spectra of [Cr] in deaerated THF at 20 °C. Excitation occurred at 550 nm, and the transient absorption signal was obtained 5 ps after excitation.

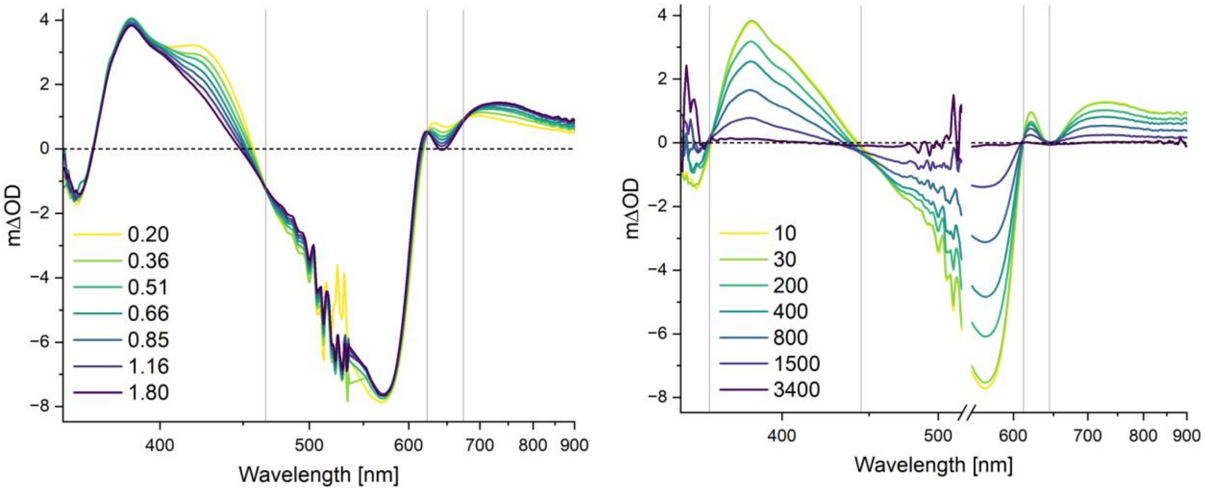

(a) (b)

Figure S41. Transient absorption spectra of [Cr] in deaerated THF on (a) short and (b) long time scales. Delay times are in picoseconds and their corresponding color codings are shown in the inserts. Data points recorded between 0 and 200 fs after the laser pulse were omitted due to coherent artifacts (from interactions with solvent and the 1 mm cuvette) at these short time scales. Isosbestic points (gray vertical lines) are observed in both time regimes and indicate state-to-state transitions. The data presented in Figure S41 is the same as that in Figure 6 of the main paper.

Global Fit Analysis

The transient absorption data of [Cr] (Figure S39) was modelled with a sequential deactivation pathway:

$$\textbf{“hot”}\ {}^{3}\textbf{MLCT} \xrightarrow{k_{Cr,1}} {}^{3}\textbf{MLCT} \xrightarrow{k_{Cr,2}} {}^{1}\textbf{GS}$$

where $k_{Cr,1}$ represents the rate constant of vibrational relaxation to the lowest vibrational level of the ^{3}MLCT state and $k_{Cr,2}$ is the rate constant describing the relaxation of the ^{3}MLCT state back to the ground state, ^{1}GS. The lifetime of intersystem crossing from the ^{1}MLCT excited state to the “hot” ^{3}MLCT excited state for related d^6 metal complexes has previously been determined to take place faster than 200 fs,[13–17] which is faster than the time resolution of our experiment.

The rate constants for the deactivation of the individual excited states of [Cr] in deaerated THF were determined to $k_{Cr,1}$ = 1.10 ps^{-1} and $k_{Cr,2}$ = 9.95 × 10^{-4} ps^{-1}, translating to lifetimes of 0.9 ps and 1.0 ns, respectively (Figure S42).

The luminescence lifetime of [Cr] in deaerated THF has previously been determined to 1.1 ns,[1] which is in good agreement with the longer lifetime of 1.0 ns obtained from the global fit analysis presented here.

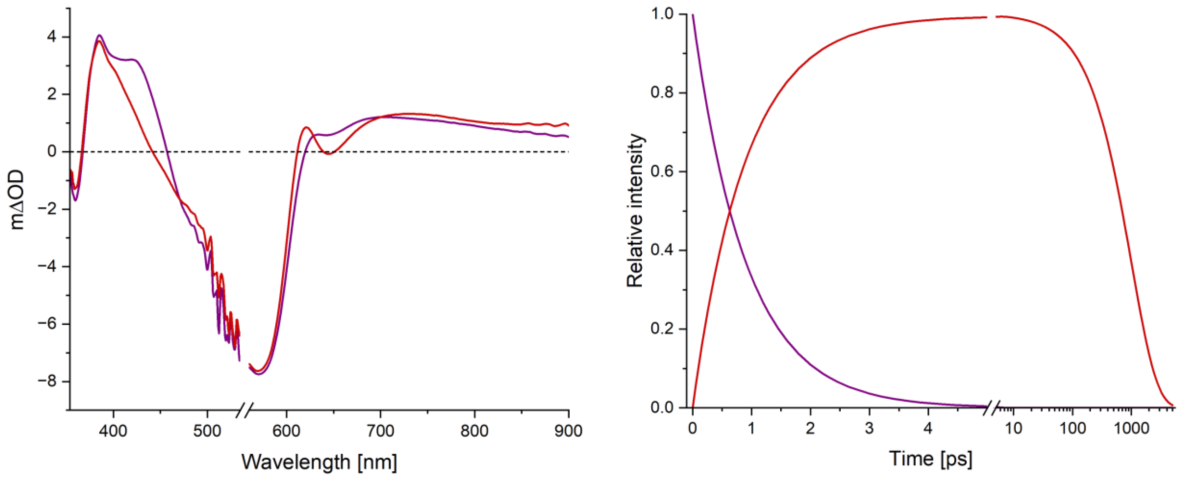

(a) (b)

Figure S42. (a) Species associated spectra of "hot ^{3}MLCT" (purple) and vibrationally cooled ^{3}MLCT (red) states and (b) the corresponding concentration profiles related to the excited state dynamics of [Cr] in deaerated THF at 20 °C.

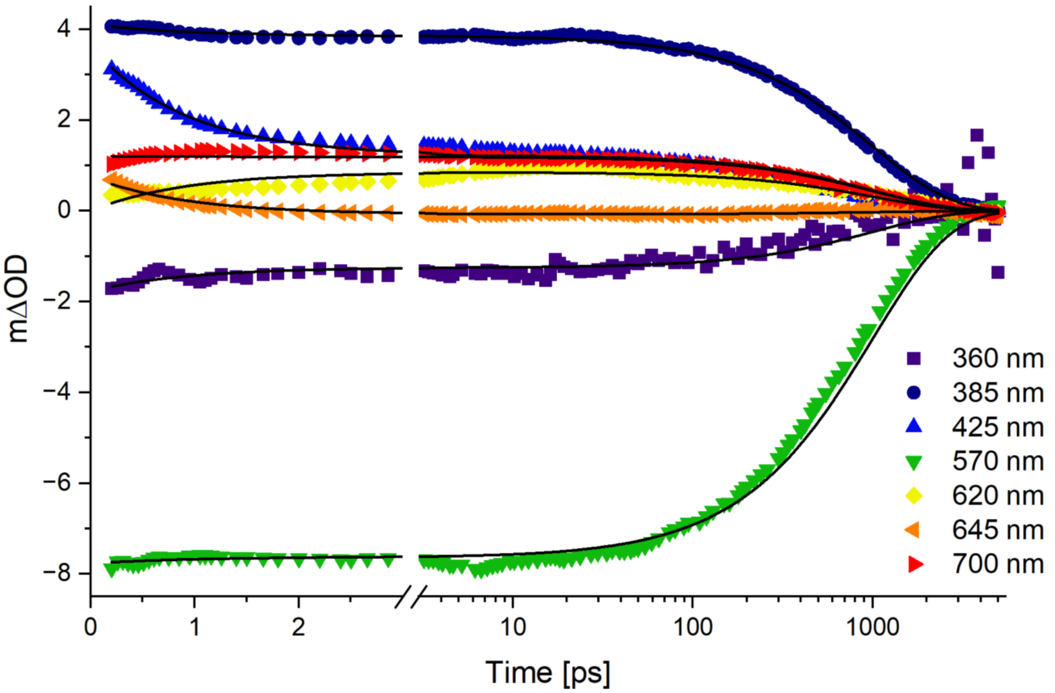

Figure S43. Measured kinetics (symbols) and results of the global fit analysis (solid lines) at selected wavelengths. Data points recorded between 0 and 200 fs after the laser pulse were omitted in the global fit analysis due to coherent artifacts at these short time scales.

8.3 [Mn]$^+$

UV–Vis Absorption Spectroscopy

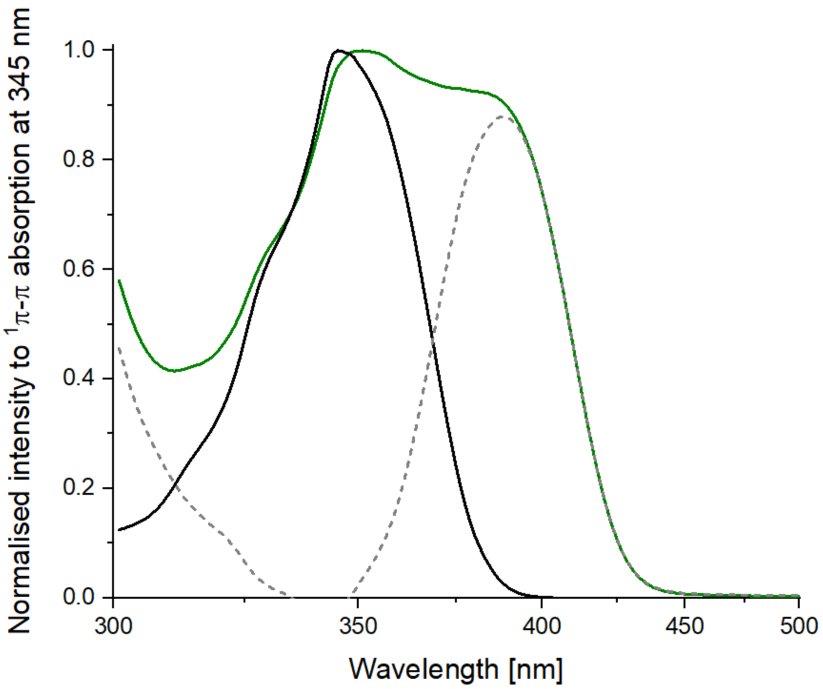

Figure S44. Normalized UV–Vis absorption spectra of L^{Pyr} (black) and [Mn]$^+$ (green) in THF, as well as the difference between these two spectra (dashed gray). The lower-energy band in the difference spectrum is attributed to the contribution of a mixed IL/MLCT excited state dominating the overall extinction in the respective spectral region.

Emission Properties

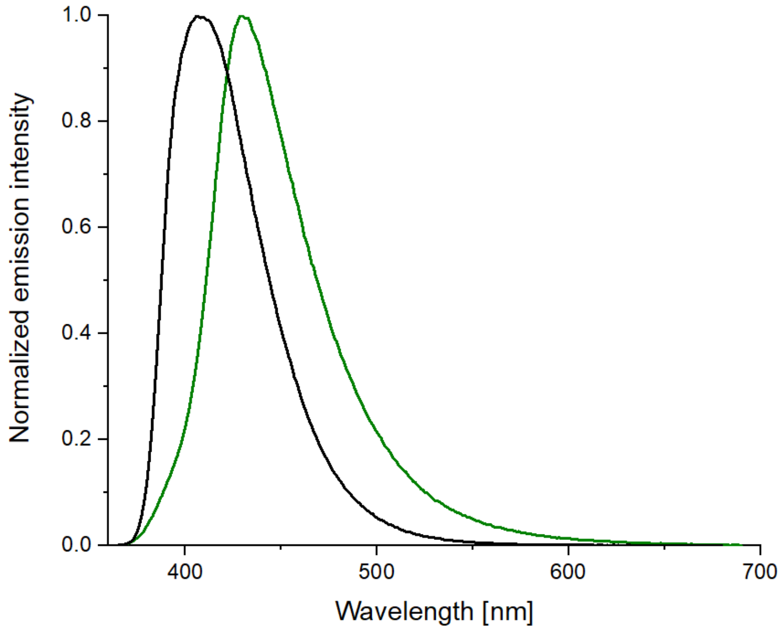

Figure S45. Emission spectra of L^{Pyr} (black) and $[Mn]^+$ (green) in aerated THF at 20 °C. Excitation occurred at 350 nm in both cases. The emission band maximum of $[Mn]^+$ is redshifted by 1300 cm^{-1} relative to that of L^{Pyr}.

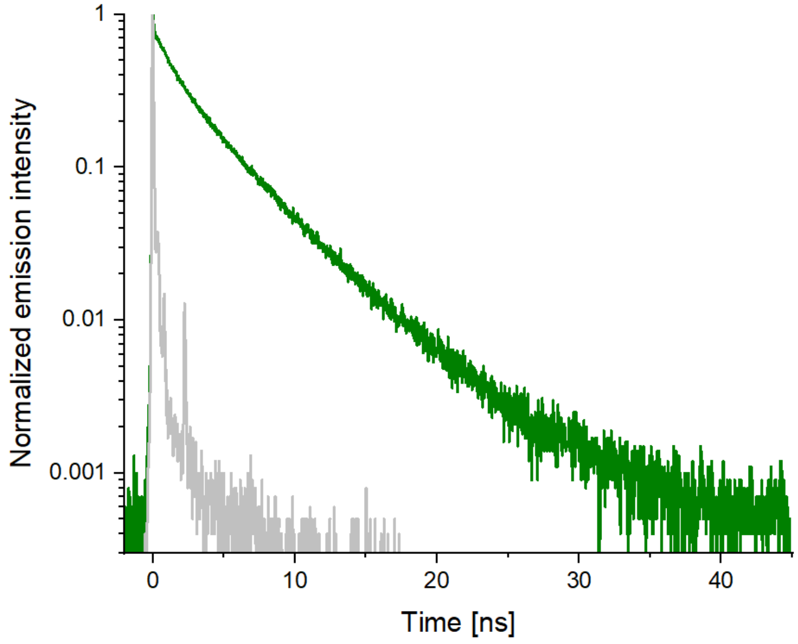

Figure S46. Luminescence decay of $[Mn]^+$ in THF (green) at 415 nm and the instrument response function (IRF, gray). Excitation occurred at 375 nm. τ_1 = 1.28 ns (41%) and τ_1 = 4.42 ns (59%), resulting in a weighted average lifetime of 3.13 ns.

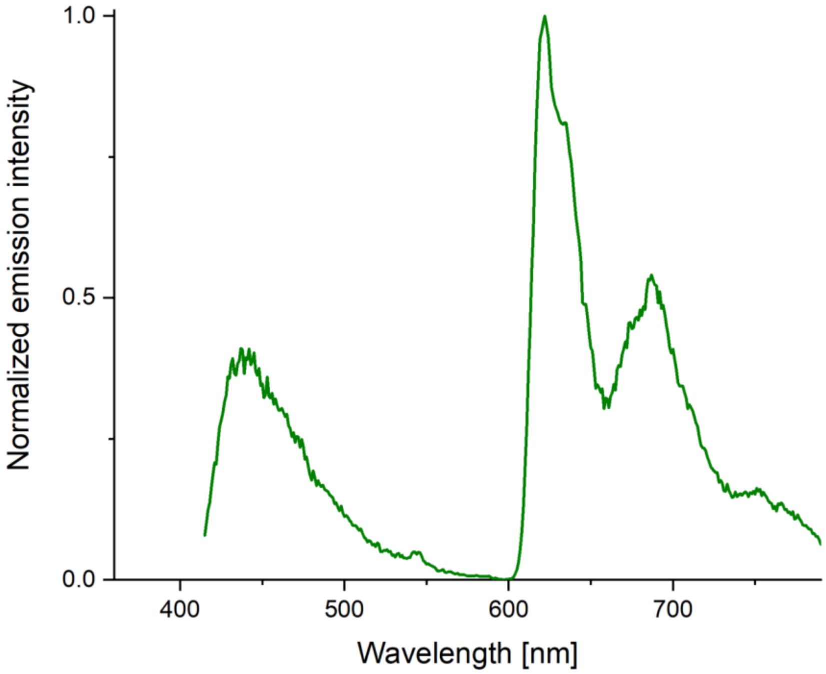

Figure S47. Emission spectrum of $[Mn]^+$ in 2-MeTHF at 77 K after excitation at 400 nm.

Table S8. Determination of the (ligand-based, i.e. $^1IL_{Pyr}$-like) fluorescence quantum yield of $[Mn]^+$ in THF relative to DPA in aerated cyclohexane at 20 °C. Excitation occurred at 350 nm.

<table>
<tr><th></th><th>$[Mn]^+$</th><th>9,10-diphenylanthracene</th></tr>
<tr><td>UV-Vis absorption spectra</td><td>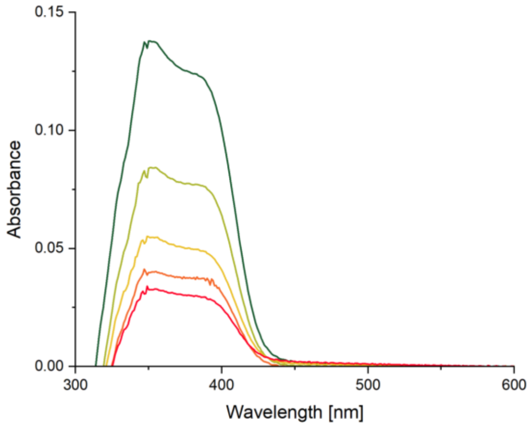
</td><td>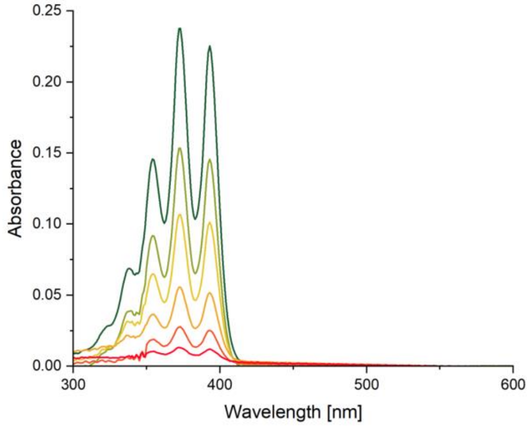
</td></tr>
<tr><td>Emission spectra</td><td>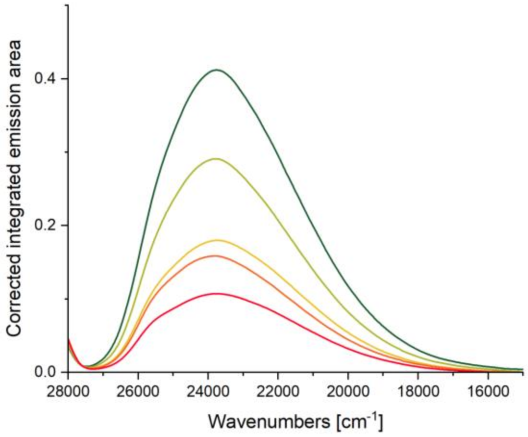
</td><td>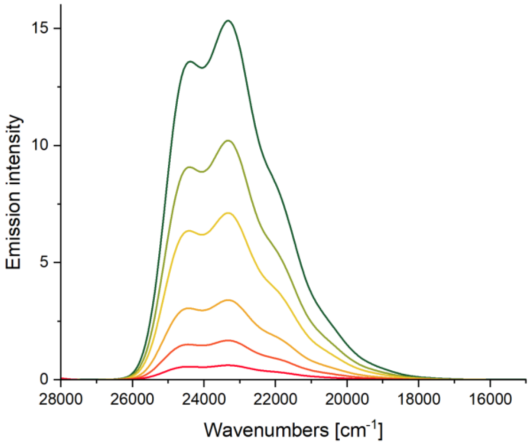
</td></tr>
<tr><td>Linear correlation</td><td>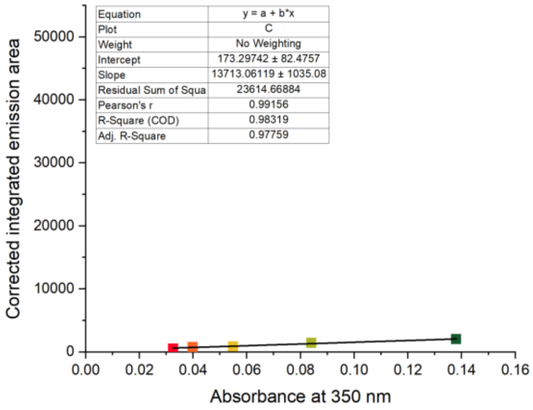
</td><td>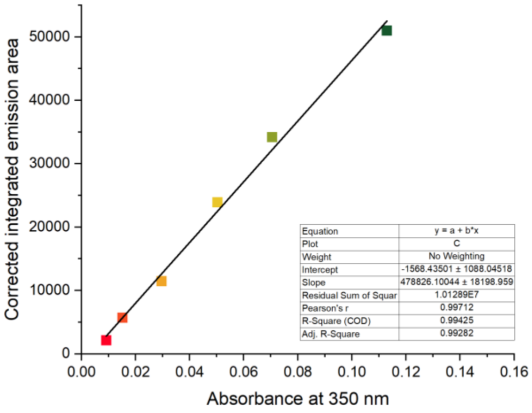
</td></tr>
</table>

Table S9. Fluorescence properties of $[Mn]^+$ in THF at 20 °C. Excitation occurred at 350 or 375 nm.

$\lambda_{abs,max}$ [nm]	$\lambda_{EM,max}$ [nm]	τ_{em} [ns]	φ
350	430	3.1	0.024

Excitation spectroscopy

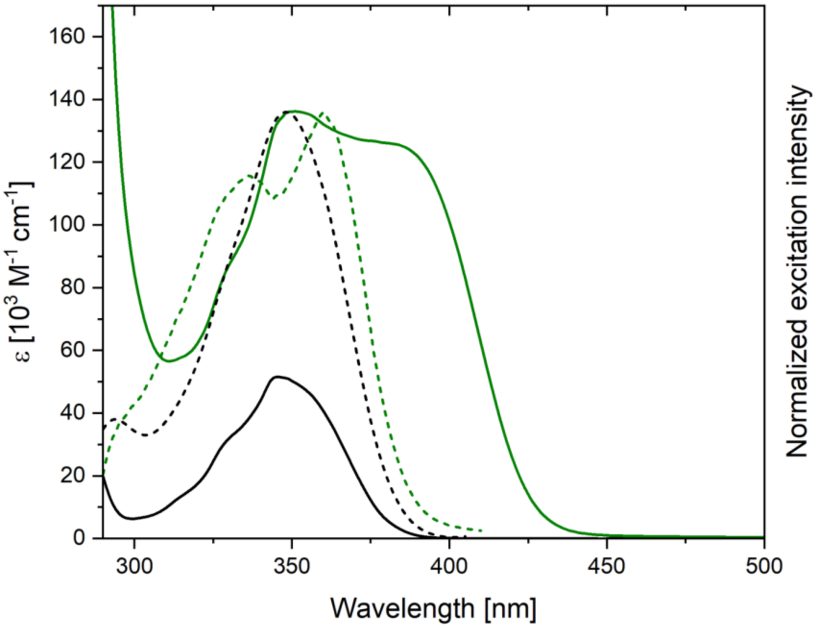

Figure S48. UV–Vis absorption (solid) and the excitation (dashed) spectra of $[Mn]^+$ (green, λ_{det} = 415 nm) and L^{Pyr} (black, λ_{det} = 411 nm) in THF at 20 °C.

Excited state dynamics of $[Mn]^+$ on longer time scales (> 10 ns)

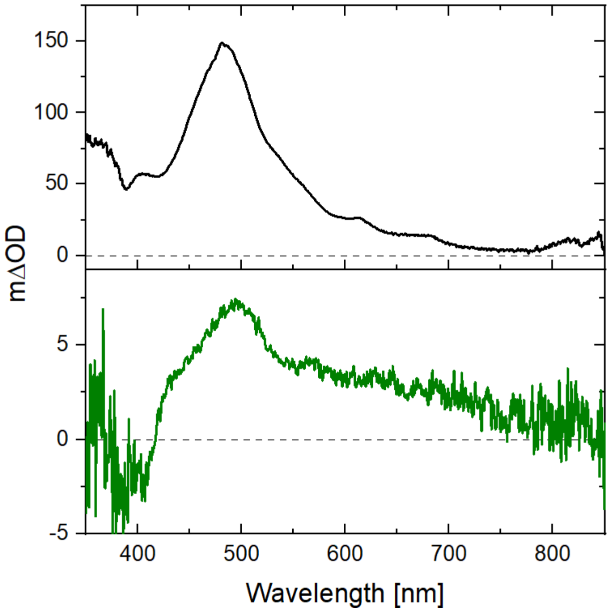

Figure S49. Transient absorption spectrum of $[Mn]^+$ in deaerated THF (green) at 20 °C. Excitation occurred at 430 nm. The signal was time-integrated for 200 ns immediately after the laser pulse. Sensitized triplet absorption spectrum of L^{Pyr} in deaerated THF (black) recorded after selective excitation of an iridium(III) sensitizer at 450 nm and subsequent triplet-triplet energy transfer to L^{Pyr}. See section 8.1 for more information.

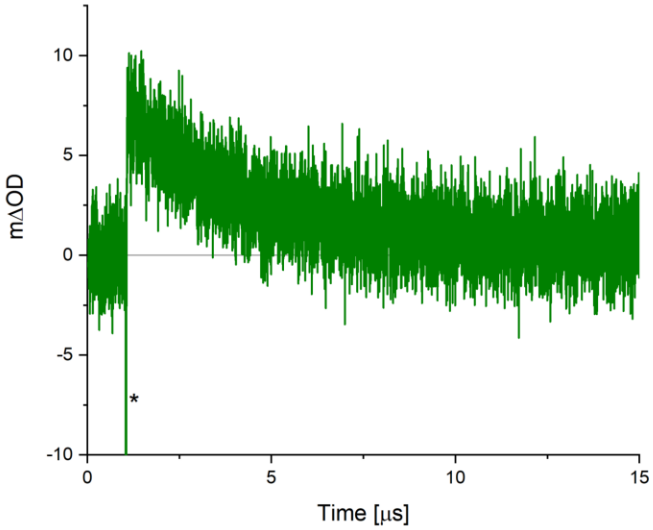

Figure S50. Decay of the transient absorption feature at 500 nm of $[Mn]^+$ in deaerated THF at 20 °C. Excitation occurred at 430 nm. The long-lived decay component has a lifetime of 2.9 μs. * = The gain of the instrument settings was maximized in this experiment due to the low intensity of the transient absorption signal, hence the negative signal (not time-resolved) likely originates from ($^1IL_{Pyr}$) fluorescence emitted by $[Mn]^+$ (see Figure S45).

Excited state dynamics on early time scales (< 6 ns)

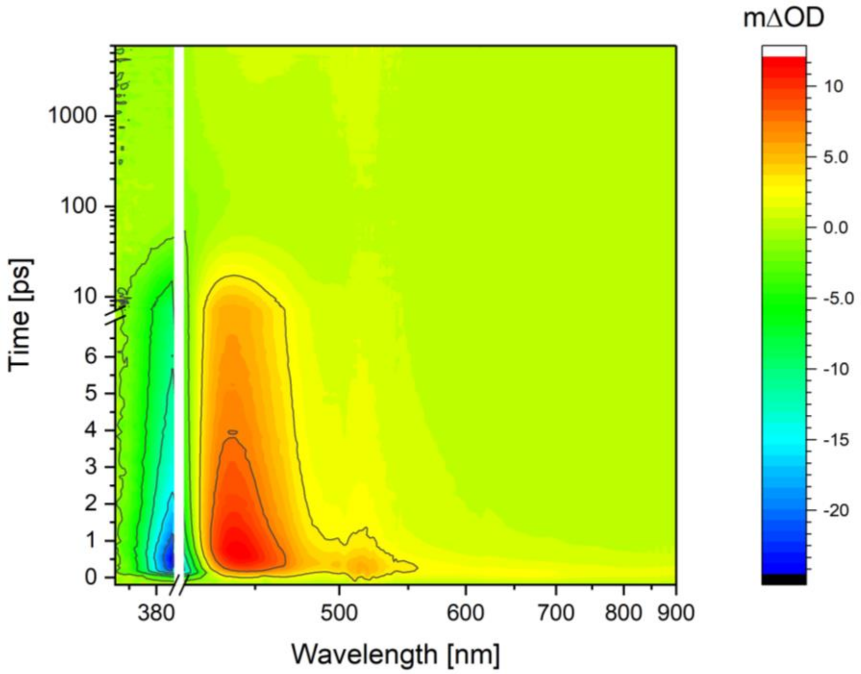

Figure S51. 2D contour plot showing the transient absorption spectra of $[Mn]^+$ in deaerated THF at 20 °C as a function of delay time between the pump and probe. Excitation occurred at 400 nm.

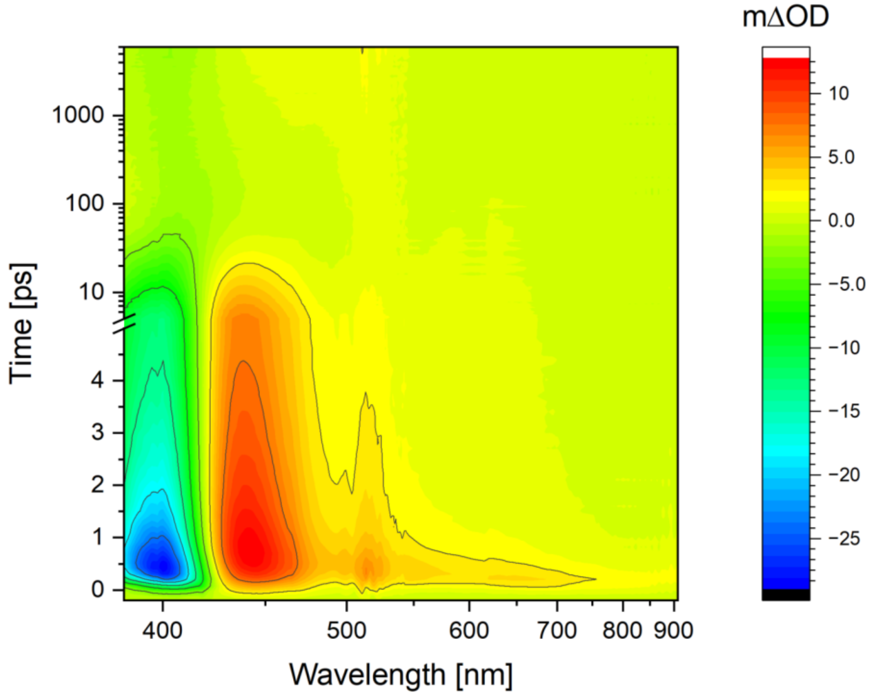

Figure S52. 2D contour plot showing the transient absorption spectra of $[Mn]^+$ in deaerated THF at 20 °C as a function of delay time between the pump and probe. Excitation occurred at 370 nm.

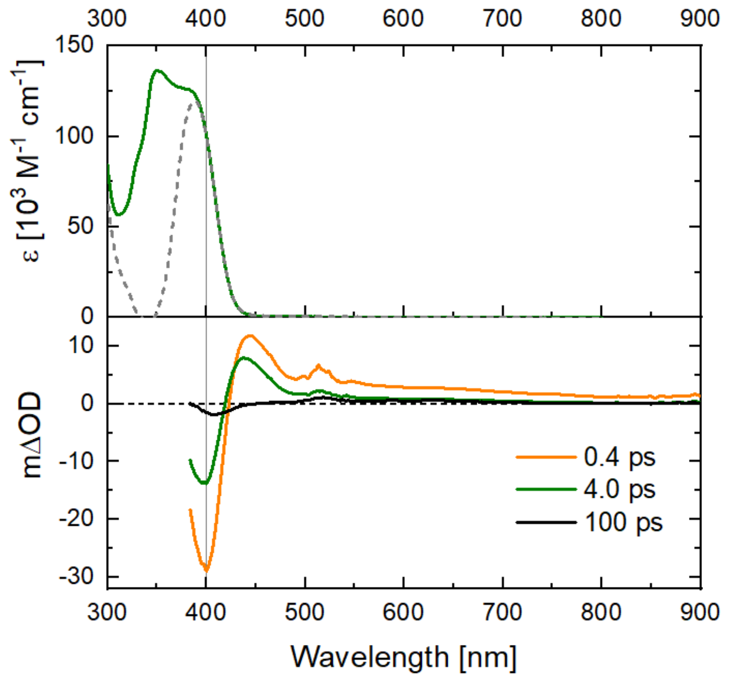

Figure S53. UV–Vis absorption (top, green) and transient absorption (bottom) spectra of $[Mn]^+$ in deaerated THF at 20 °C. Delay times after the laser pulse are noted in the insert. Excitation occurred at 370 nm. The difference between the UV–Vis ground absorption spectra of $[Mn]^+$ and L^{Pyr} is shown in dashed gray (top).

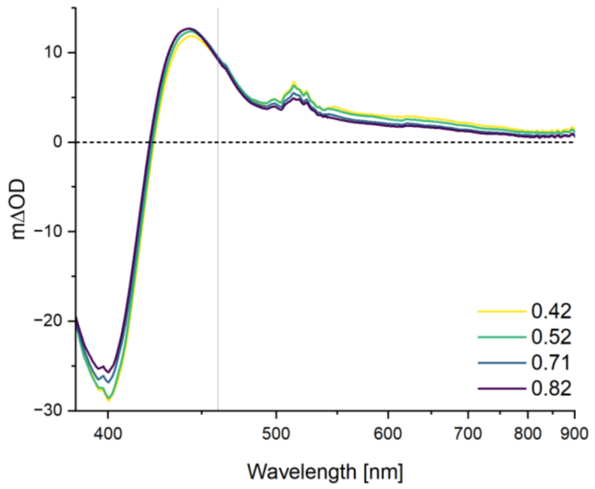

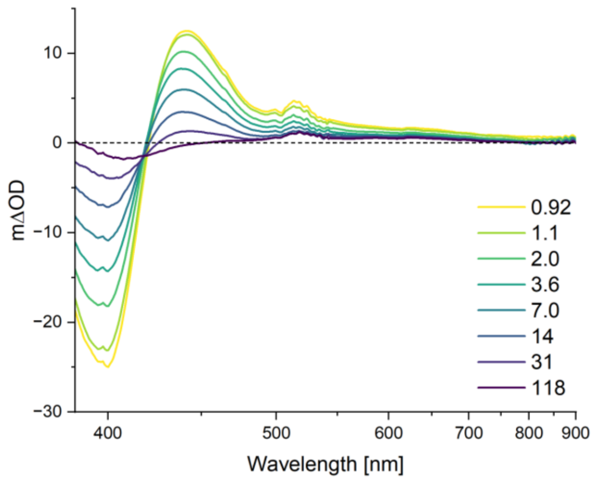

(a) (b)

Figure S54. Transient absorption spectra of $[Mn]^+$ in THF on (a) short and (b) long time scales. The transient absorption signal has not fully evolved till 0.42 ps after the laser pulse, hence the first spectrum shown is at this delay time. Delay times are in ps and their corresponding color codings are shown in the inserts. Isosbestic points (gray vertical lines) are in (a), indicating state-to-state transitions.

Global Fit Analysis

The amplitude of the GSB signal is reduced significantly within the first 100 ps, alongside the formation of the spectral feature (Figure S54b) related to the microsecond component identified as a $^3IL_{Pyr}$ state. The $^3IL_{Pyr}$ spectrum does not have intense absorption around 400 nm (Figure S49), suggesting that the decay of the GSB cannot be explained by growing absorption in the same spectral range. This behavior indicates that a significant portion of the excited state population returns to the ground state within the first 100 ps, whereas only a minor fraction populates the $^3IL_{Pyr}$ state. Based on these experimental observations, the transient absorption data of $[Mn]^+$ (Figure S52) was modelled with a branching deactivation pathway:

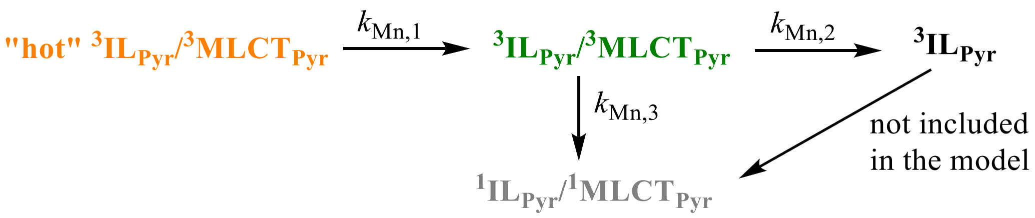

where $k_{Mn,1}$ represents the rate constant of vibrational cooling to the lowest vibrational level of the $^3IL_{\mathbf{Pyr}}/^3MLCT_{\mathbf{Pyr}}$ excited state, $k_{Mn,2}$ is the rate constant describing the deactivation of the $^3IL_{\mathbf{Pyr}}/^3MLCT_{\mathbf{Pyr}}$ state, which progresses into a long-lived $^3IL_{Pyr}$ state (τ = 2.9 µs), as characterized with our ns transient absorption spectroscopy setup (Figure S49) and $k_{Mn,3}$ is the rate constant of direct deactivation of the $^3IL_{\mathbf{Pyr}}/^3MLCT_{\mathbf{Pyr}}$ excited state to the ground state. The decay of $^3IL_{Pyr}$ back to the ground state was not included in the model as the lifetime of 2.9 µs is considered infinitely long in the time window (6 ns) of this ultrafast transient absorption spectroscopy measurement, hence there are not sufficient data points to model the decay of $^3IL_{Pyr}$.

The global fit analysis resulted in the rate constants $k_{Mn,1}$ = 0.80 ps^{-1}, $k_{Mn,2}$ = 0.011 ps^{-1} and $k_{Mn,3}$ = 0.061 i.e. lifetimes of 1.2 ps, 90 ps and 16 ps, respectively (Figure S55). For comparison, the lifetime of the vibrational cooling process of [Cr] in THF was determined to 0.9 ps (Figure S42). The splitting ratio between $k_{Mn,2}$ and $k_{Mn,3}$ is estimated to 15:85 (Figure S55b), meaning that population of $^3IL_{yr}$ is a minor pathway. The total rate constant of decay of the $^3IL/^3MLCT$ state is the sum of the two decay components i.e. 0.072 ps^{-1}, equivalent to an overall lifetime of 14 ps.

Reassuring, the species associated spectrum of the slowest decay component has a great resemblance to the transient absorption spectrum of $[Mn]^+$ recorded with an integration time of 200 ns after excitation (Figure S56).

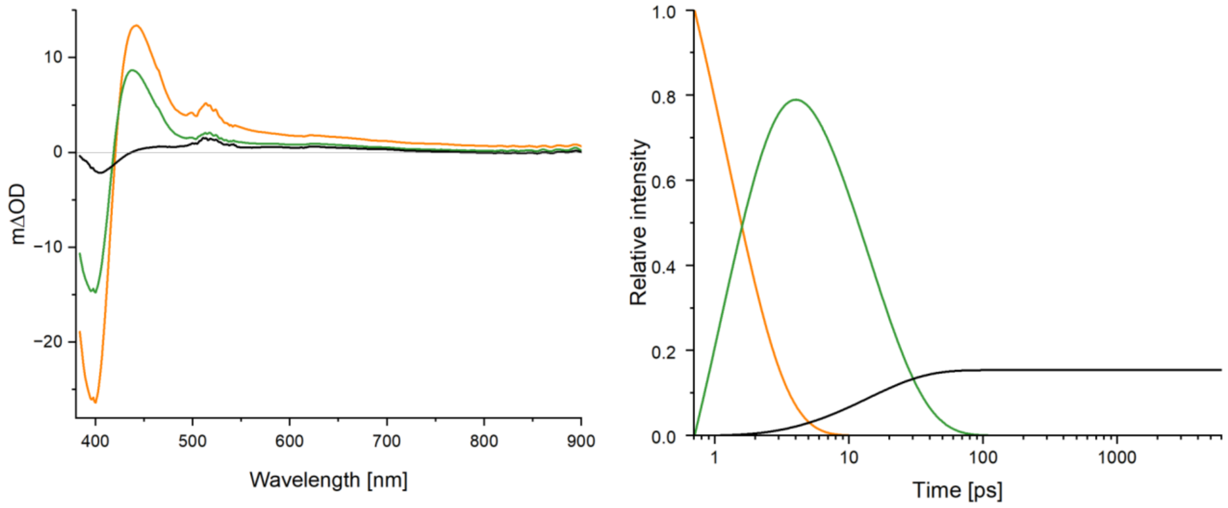

(a) (b)

Figure S55. (a) Species associated spectra of "hot" $^{3}IL_{\mathbf{Pyr}}$/$^{3}MLCT_{\mathbf{Pyr}}$ (orange), vibrationally cooled $^{3}IL_{\mathbf{Pyr}}$/$^{3}MLCT_{\mathbf{Pyr}}$ (green) and $^{3}IL_{Pyr}$ (black), along with (b) the corresponding calculated concentration profiles related to the excited state dynamics of $[Mn]^{+}$ in deaerated THF.

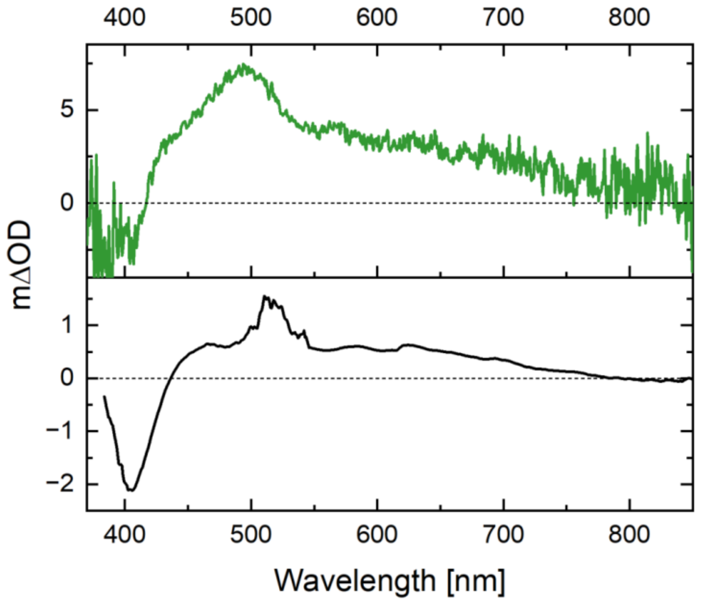

Figure S56. Species associated spectra of the $^{3}IL_{Pyr}$ state (black) and transient absorption spectra of $[Mn]^{+}$ in deaerated THF (green) at 20 °C. Excitation occurred at 430 nm. The transient absorption signal was time-integrated for 200 ns immediately after the laser pulse.

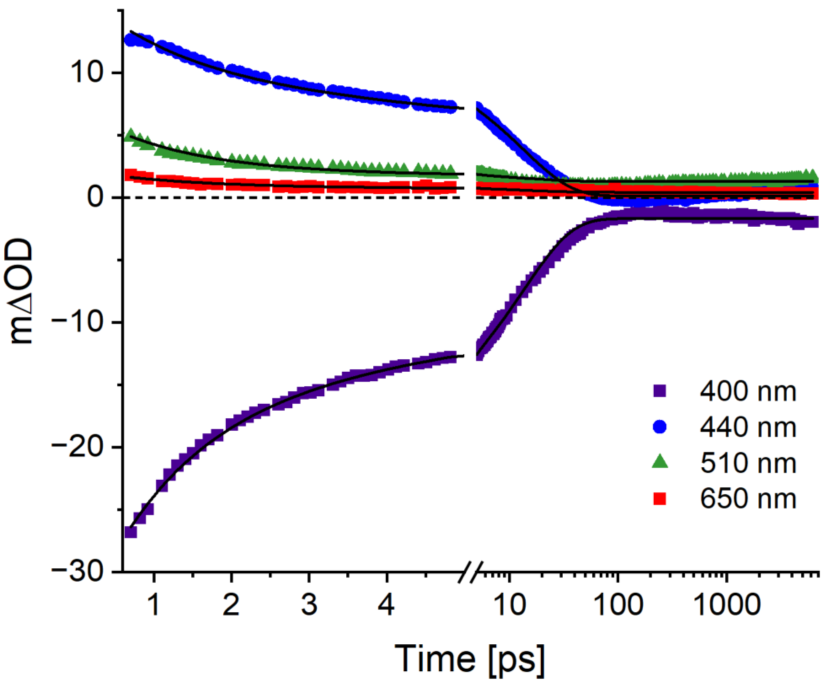

Figure S57. Measured kinetics (symbols) and the result of the global fit analysis (solid lines) at selected wavelengths for $[Mn]^+$ in deaerated THF. Excitation occurred at 370 nm.

Upon excitation of $[Mn]^+$, only 2% of the absorbed photons end up in the radiative relaxation pathway resulting in $^1IL_{Pyr}$-like emission (according to the fluorescence quantum yield), and the remaining 98% of absorbed photons must consequently end up in non-radiative relaxation pathways from $^3IL_{Pyr}$/$^3MLCT_{Pyr}$. In other words, this means that the excited state dynamics monitored by transient absorption spectroscopy are dominated by the contribution from the non-radiative deactivation pathway. Whereas it would be reassuring to identify the decay of $^1IL_{Pyr}$ in transient absorption data of $[Mn]^+$ and include it in the global fit analysis, the minor contribution of this pathway to the overall excited-state deactivation of $[Mn]^+$ makes this infeasible in practice. Corroborating, the global fit analysis is in good agreement with the data (Figure S58).

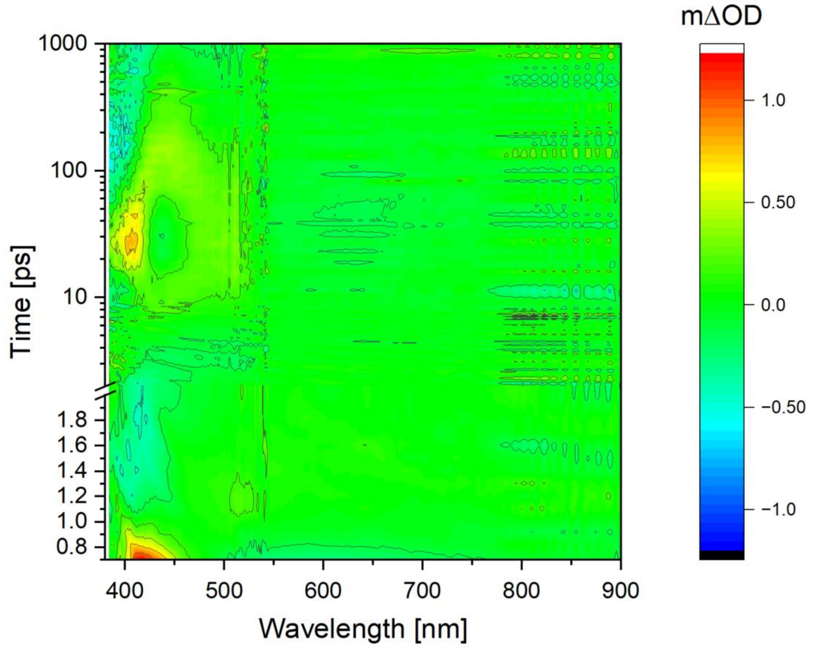

Figure S58. 2D contour plot showing the residual mΔOD between the measured transient absorption spectra of $[Mn]^{+}$ in deaerated THF at 20 °C as a function of delay time between the pump and probe and those modelled in the global fit analysis.

8.4 $[Fe]^{2+}$

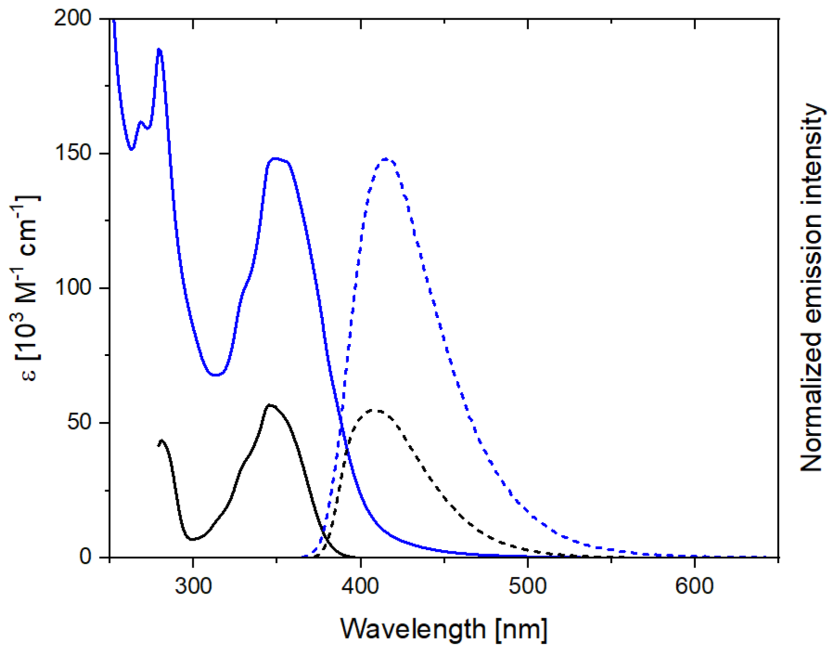

Figure S59. UV–Vis absorption (solid) and emission (dashed) spectra of L^{Pyr} (black) and $[Fe]^{2+}$ (blue) aerated in THF at 20 °C. Excitation occurred at 350 nm.

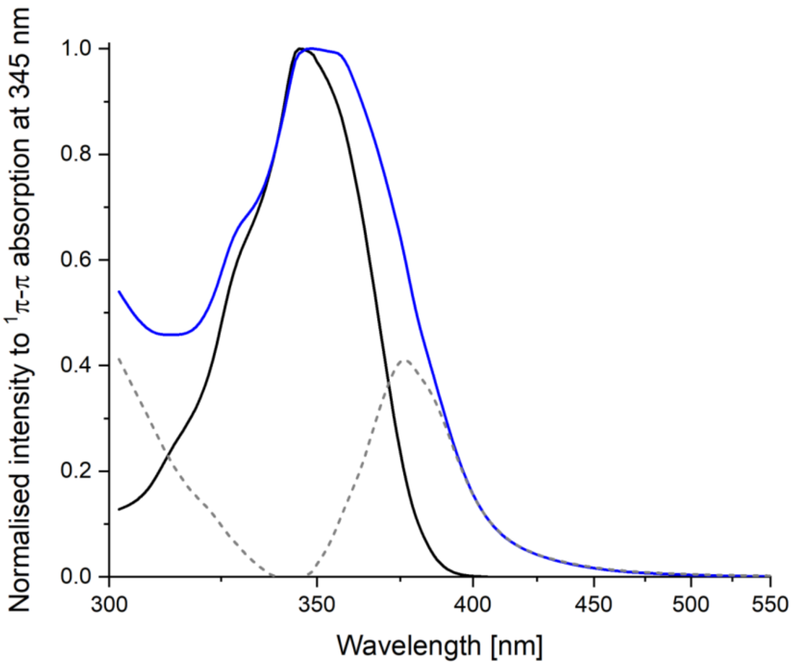

Figure S60. Normalized UV–Vis absorption spectra of L^{Pyr} (black) and $[Fe]^{2+}$ (blue) in THF as well as the difference between these two spectra (dashed gray). The longer-wavelength part of the difference spectrum seems largely attributable to the spectral contribution stemming from ILCT with minor admixed metal character.

Ideally, we would have included the isostructual zinc(II) complex, $[Zn(L^{Pyr})_3]^{2+}$, in the study for comparison of optical properties with $[Mn]^+$ and $[Fe]^{2+}$ to more clearly identify ligand-centered contribution to the individual absorption spectra. Our synthetic efforts towards obtaining $[Zn(L^{Pyr})_3](PF_6)_2$ were, however, unsuccessful due to decomposition of L^{Pyr} during the complexation with Zn^{2+}. Ligand decomposition has never been observed in the syntheses of [Cr], $[Mn]PF_6$, $[Fe](PF_6)_2$ and $[FeCl_2]$, and recovery of excess/unreacted L^{Pyr} from reaction mixtures of these complexes was always feasible. Isocyanide groups are known to be sensitive to acidic conditions,[18] hence intrinsically high Lewis acidity of the metal ion could potentially cause undesirable ligand decomposition. Moreover, the lack of ligand field stabilization in the $3d^{10}$ electron configuration makes zinc(II) complexes inherently less stable than the low spin $3d^6$ complexes. To the best of our knowledge, no zinc(II) hexakis(isocyanide) complexes are known.[19]

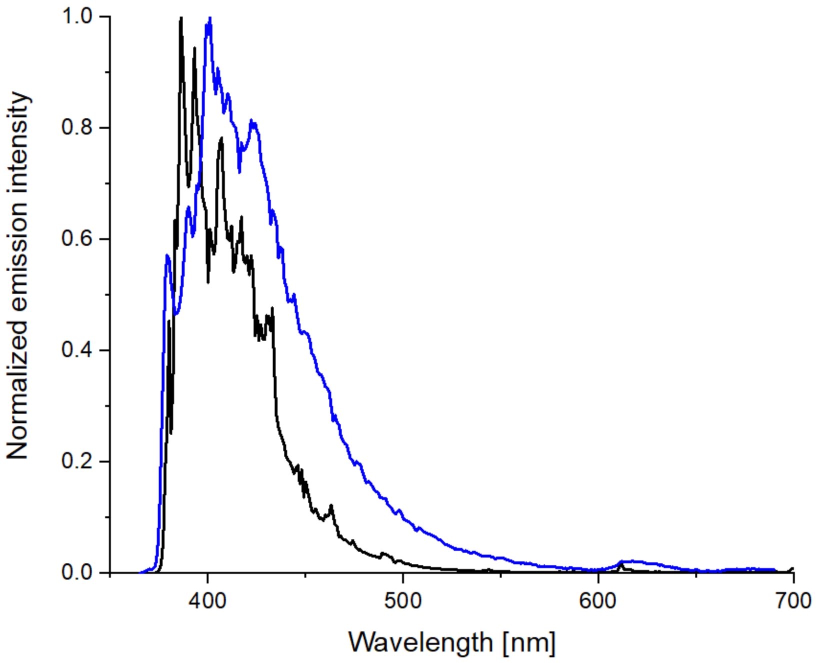

Figure S61. Emission spectra of L^{Pyr} (black) and $[Fe]^{2+}$ (blue) in 2-Me-THF at 77 K. Excitation occurred at 350 nm in both cases.

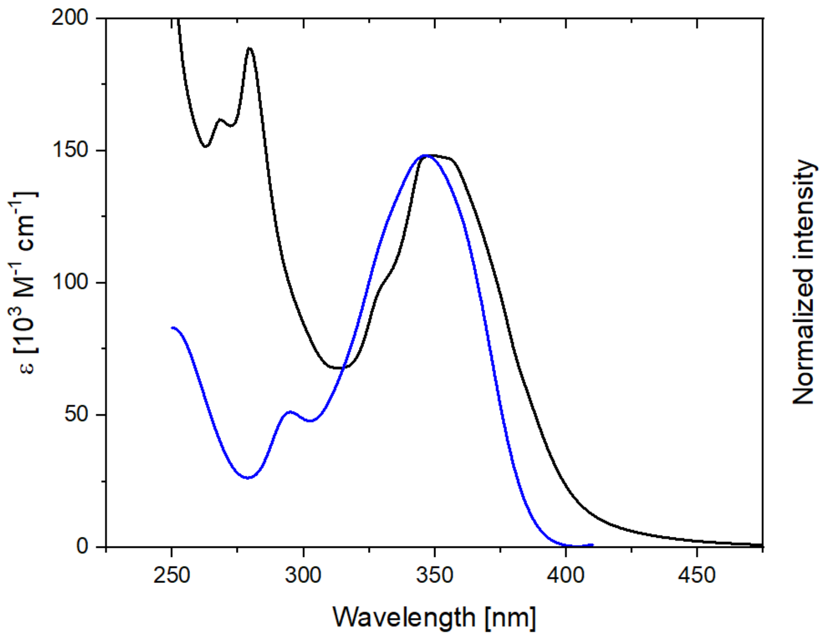

Figure S62. UV–Vis absorption (black) and excitation (blue, λ_{det} = 415 nm) spectra of $[Fe]^{2+}$ in THF at 20 °C.

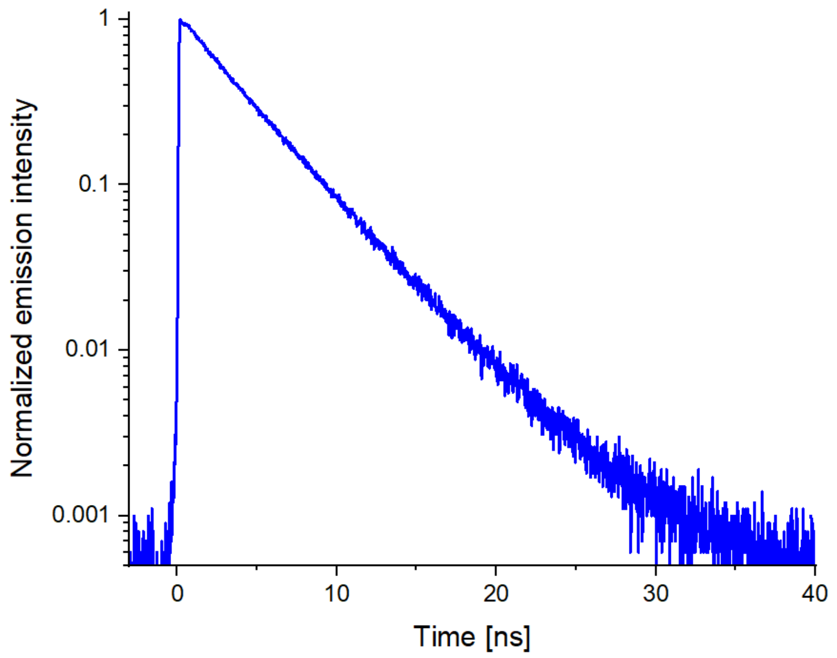

Figure S63. Luminescence decay at 411 nm of $[Fe]^{2+}$ in THF. Excitation occurred at 375 nm. The luminescence decay was fitted monoexponentially to result in a luminescence lifetime of 3.8 ns.

Table S10. Determination of the (ligand-based, i.e. $^1IL_{Pyr}$-like) fluorescence quantum yield of $[Fe]^{2+}$ in THF relative to DPA in cyclohexane at 20 °C. Excitation occurred at 350 nm.

<table>
<tr><th></th><th>$[Fe]^{2+}$</th><th>9,10-diphenylanthracene</th></tr>
<tr><td>UV-Vis absorption spectra</td><td>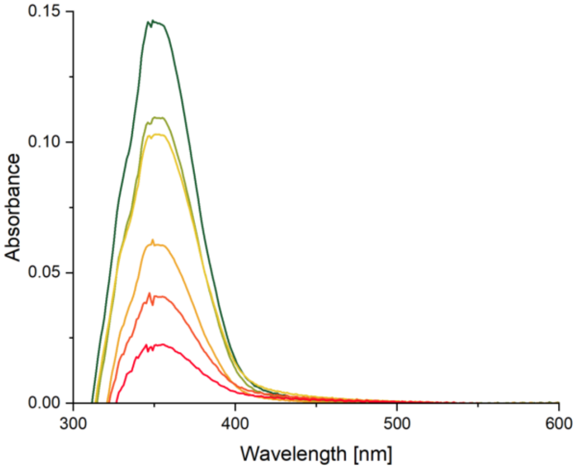
</td><td>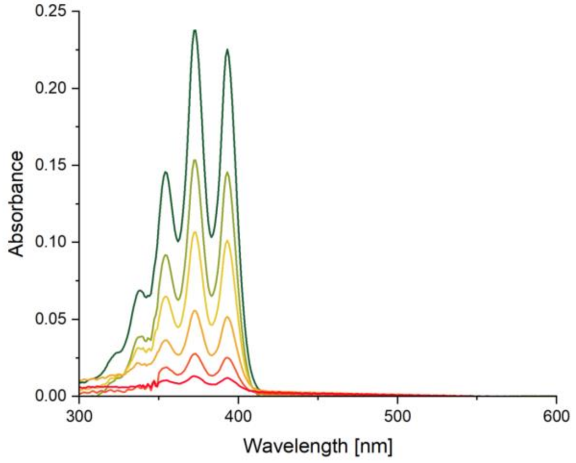
</td></tr>
<tr><td>Emission spectra</td><td>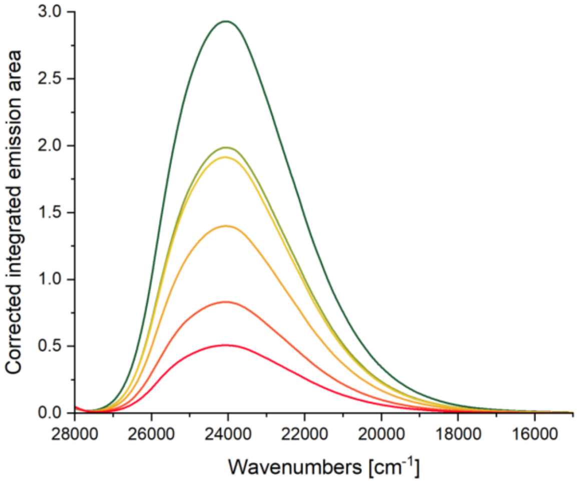
</td><td>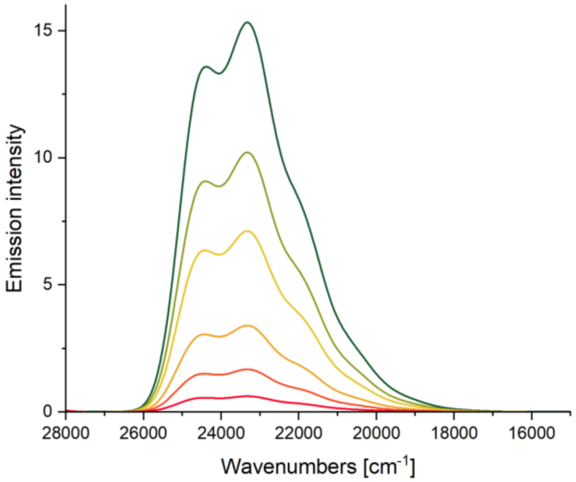
</td></tr>
<tr><td>Linear correlation</td><td>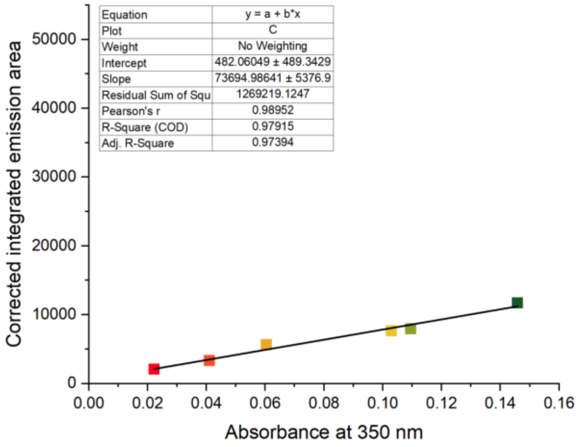
</td><td>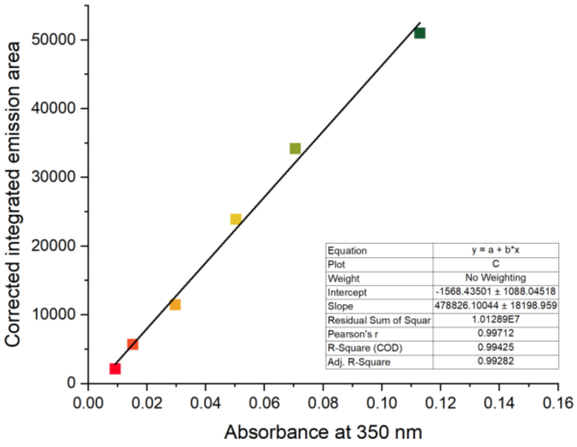
</td></tr>
</table>

Table S11. Fluorescence properties of $[Fe]^{2+}$ in THF at 20 °C. Excitation occurred at 350 or 375 nm.

$\lambda_{abs,max}$ [nm]	$\lambda_{EM,max}$ [nm]	τ_{em} [ns]	φ
350	415	3.8	0.14

Excited state dynamics

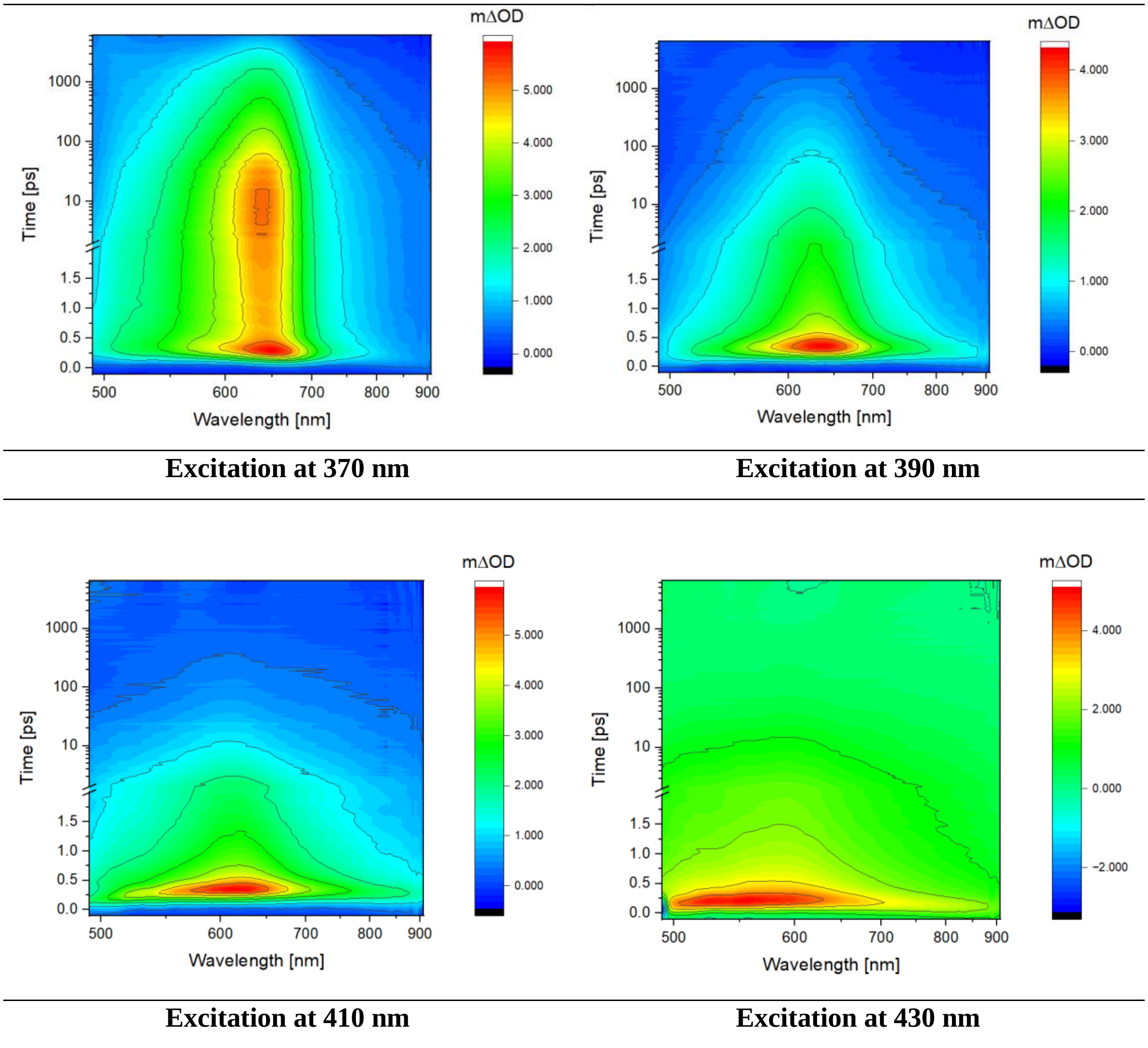

Figure S64. 2D contour plot showing the transient absorption spectra of $[Fe]^{2+}$ in aerated THF at 20 °C as a function of delay time between pump and probe.

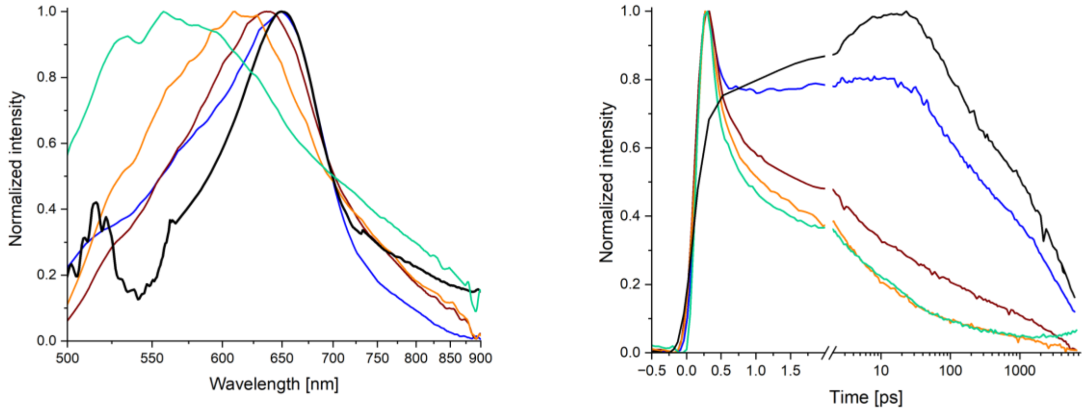

(a) (b)

Figure S65. (a) Normalized initial spectrum of $[Fe]^{2+}$ and of L^{Pyr} in THF immediately after cross phase modulations and (b) the corresponding kinetic trace monitored at the maximum value in (a). L^{Pyr} was excited at 370 nm (black) and $[Fe]^{2+}$ at 370 (blue), 390 (brown), 410 (yellow) or 430 nm (green).

By fixing the luminescence and transient absorption decays of $[Fe]^{2+}$ to 1 at 1 ns and to 0 at 6.2 ns (Figure S66), it is seen that the long-lived time component observed in the transient absorption data coincides with the luminescence decay (τ = 3.8 ns). This observation supports that the ns time component observed in the transient absorption spectra upon excitation at 370 nm is related to the depopulation of the fluorescent $^1IL_{Pyr}$-like state. Upon redshift of the excitation to 390, 410 and 430 nm, the contribution from the $^1IL_{Pyr}$ state in $[Fe]^{2+}$ gradually becomes smaller (Figure S65), and the excited state dynamics stepwise get more dominated by the non-radiative deactivation channel originating from transitions of ILCT character.

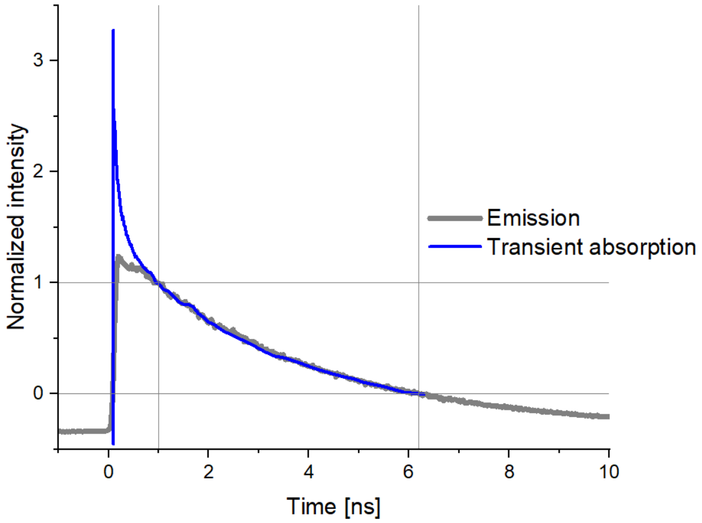

Figure S66. Normalized luminescence (λ_{exc} at 375 nm) and transient absorption (λ_{exc} at 370 nm) decay profiles of $[Fe]^{2+}$ in THF.

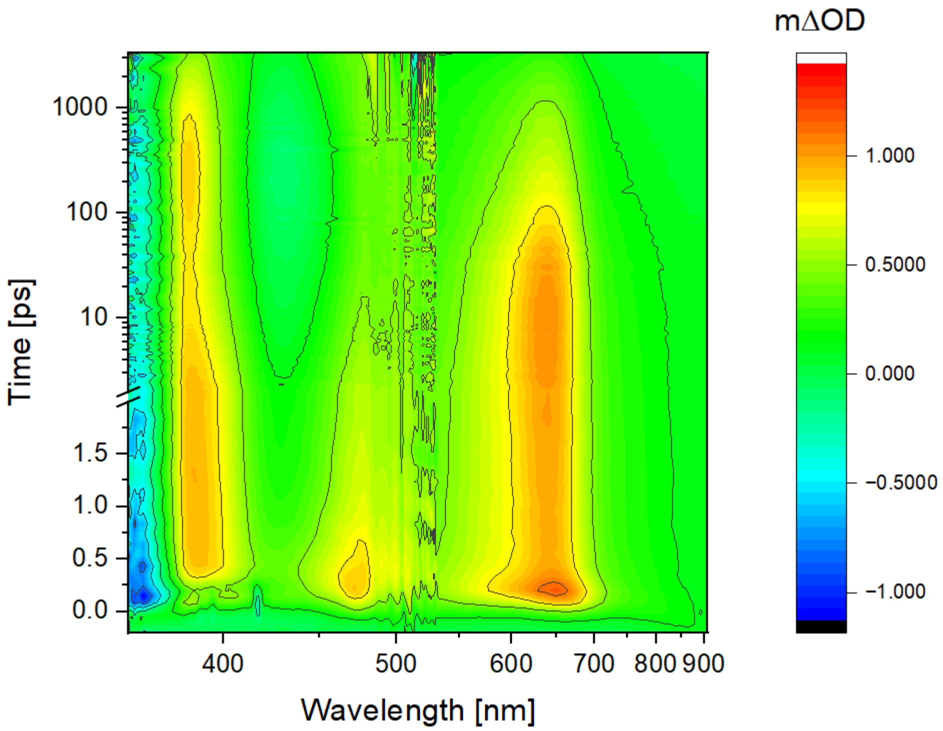

Figure S67. 2D contour plot showing the transient absorption spectra of $[Fe]^{2+}$ in aerated THF at 20 °C as a function of delay time between pump and probe. Excitation at 370 nm.

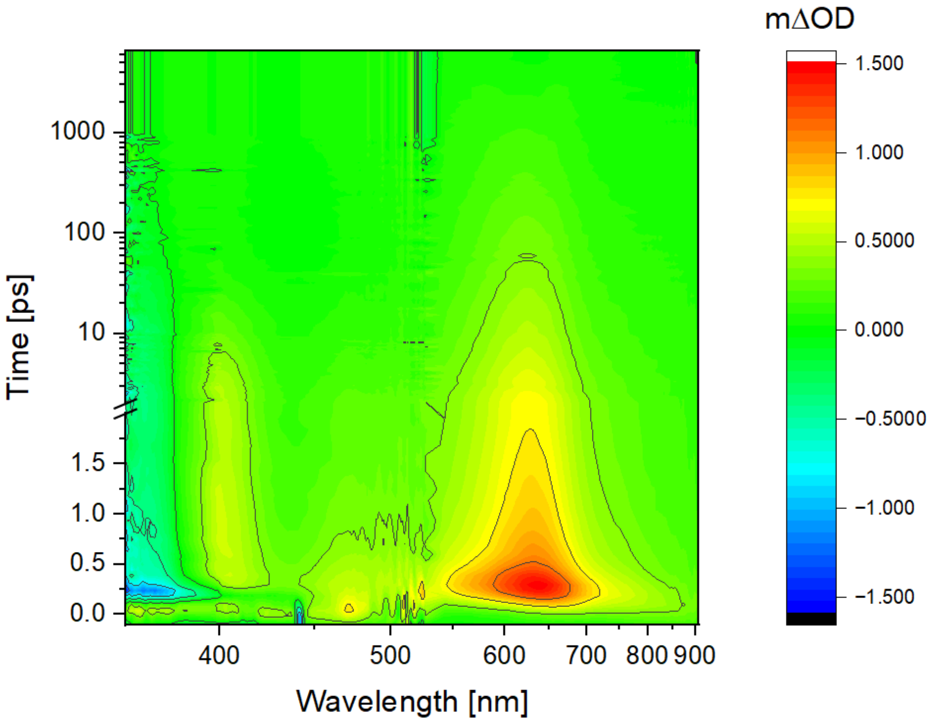

Figure S68. 2D contour plot showing the transient absorption spectra of $[Fe]^{2+}$ in aerated THF at 20 °C as a function of delay time between pump and probe. Excitation at 390 nm.

8.5 [$FeCl_2$]

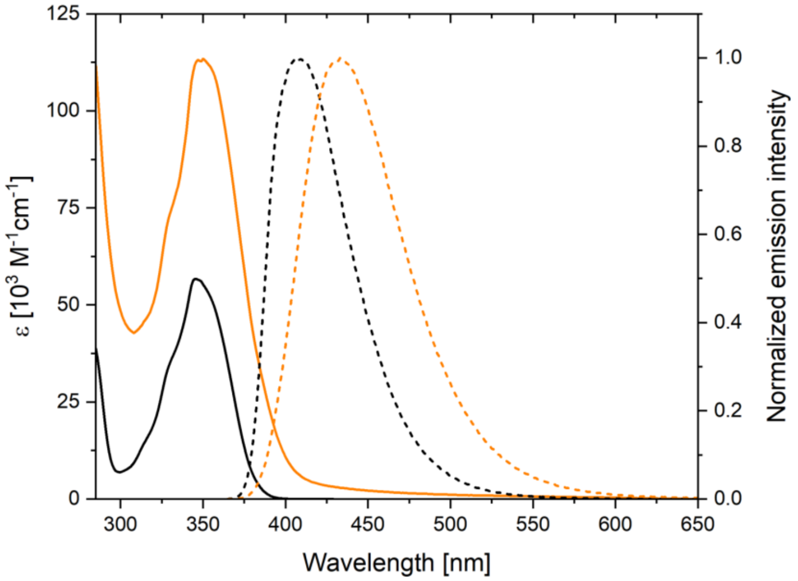

Figure S69. UV–Vis absorption (solid) and emission (dashed) spectra of L^{Pyr} (black) and [$FeCl_2$] (orange) in deaerated THF at 20 °C. Excitation occurred at 350 nm.

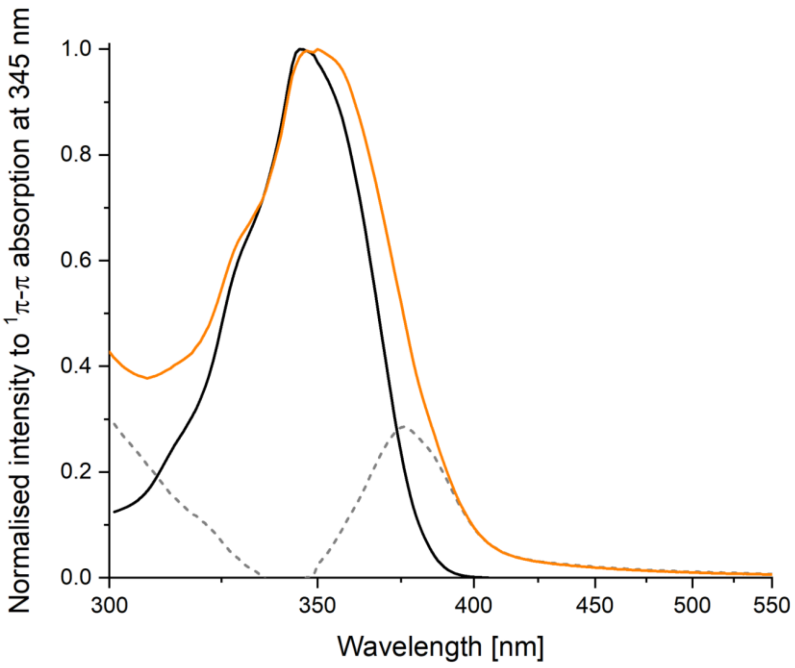

Figure S70. Normalized UV–Vis absorption spectra of L^{Pyr} (black) and [$FeCl_2$] (orange) in THF at 20 °C as well as the difference between these two spectra (dashed gray).

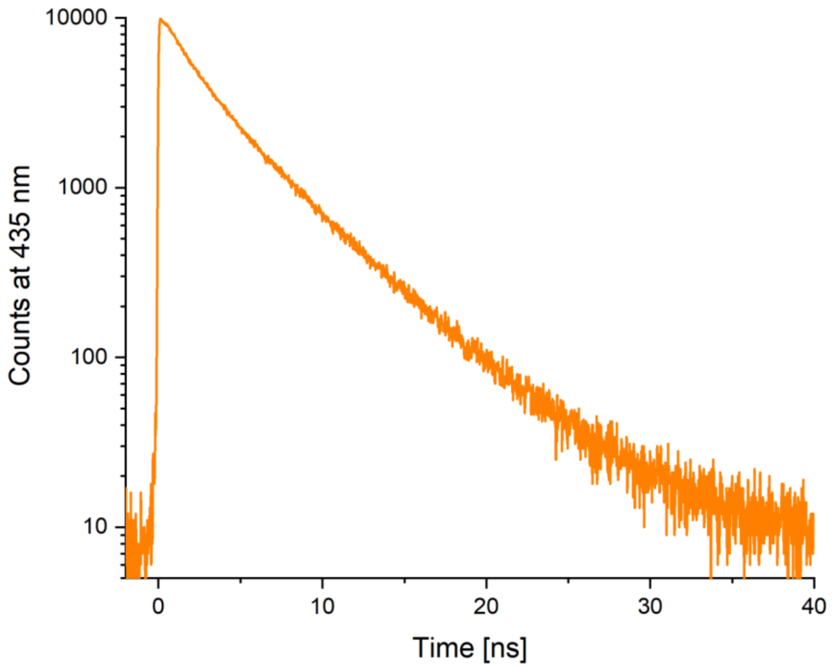

Figure S71. Emission decay of [$FeCl_2$] in deaerated THF (red) at 20 °C. Excitation occurred at 375 nm. The luminescence decay of [$FeCl_2$] was fitted with a biexponential function to obtain the lifetimes 2.24 (65%) and 5.47 (35%), resulting in an averaged lifetime of 3.4 ns.

Table S12. Fluorescence quantum yield determination of [$FeCl_2$] in THF relative to DPA in cyclohexane at 20 °C. Excitation occurred at 350 nm.

Columns: [$FeCl_2$] | 9,10-diphenylanthracene

UV-Vis absorption spectra

[$FeCl_2$]:

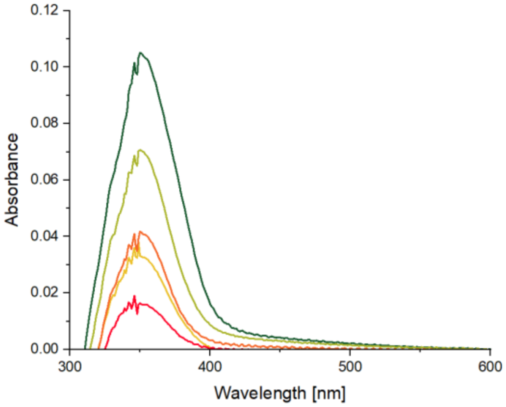

9,10-diphenylanthracene:

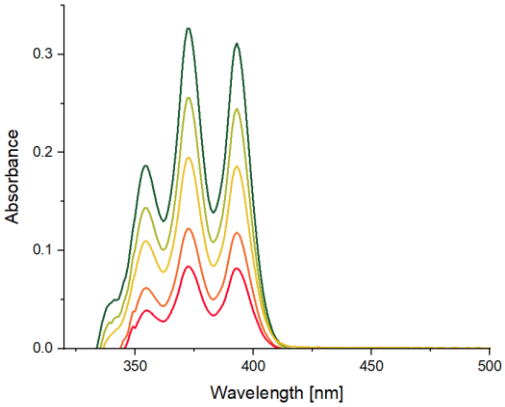

Emission spectra

[$FeCl_2$]:

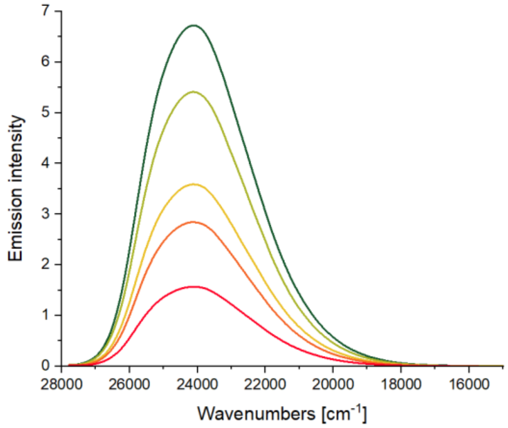

9,10-diphenylanthracene:

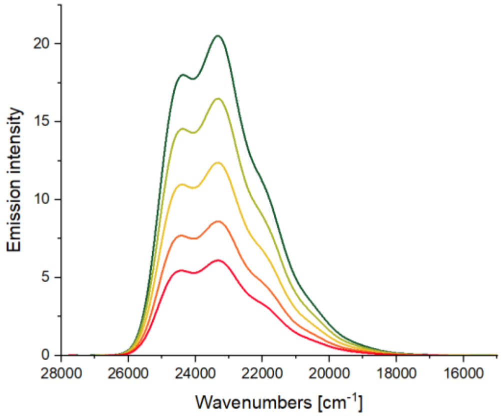

Linear correlation

[$FeCl_2$]:

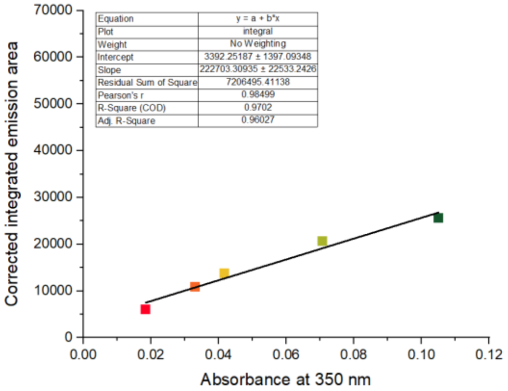

9,10-diphenylanthracene:

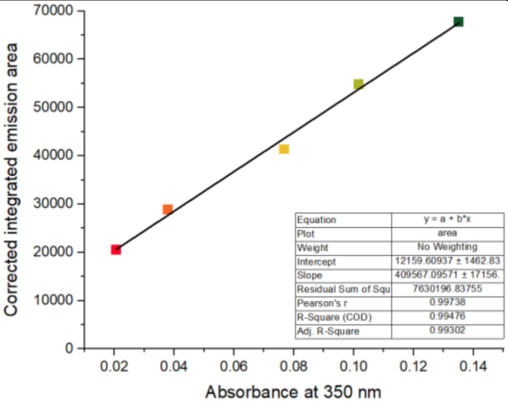

Table S13. Emission properties of [$FeCl_2$] in THF at 20 °C. Excitation occurred at 350 nm or 375 nm.

$\lambda_{abs,max}$ [nm]	$\lambda_{EM,max}$ [nm]	τ_1, τ_2 [ns]	$\tau_{average}$ [ns]	φ
350	433	2.24 (65%), 5.47 (35%)	3.4	0.48

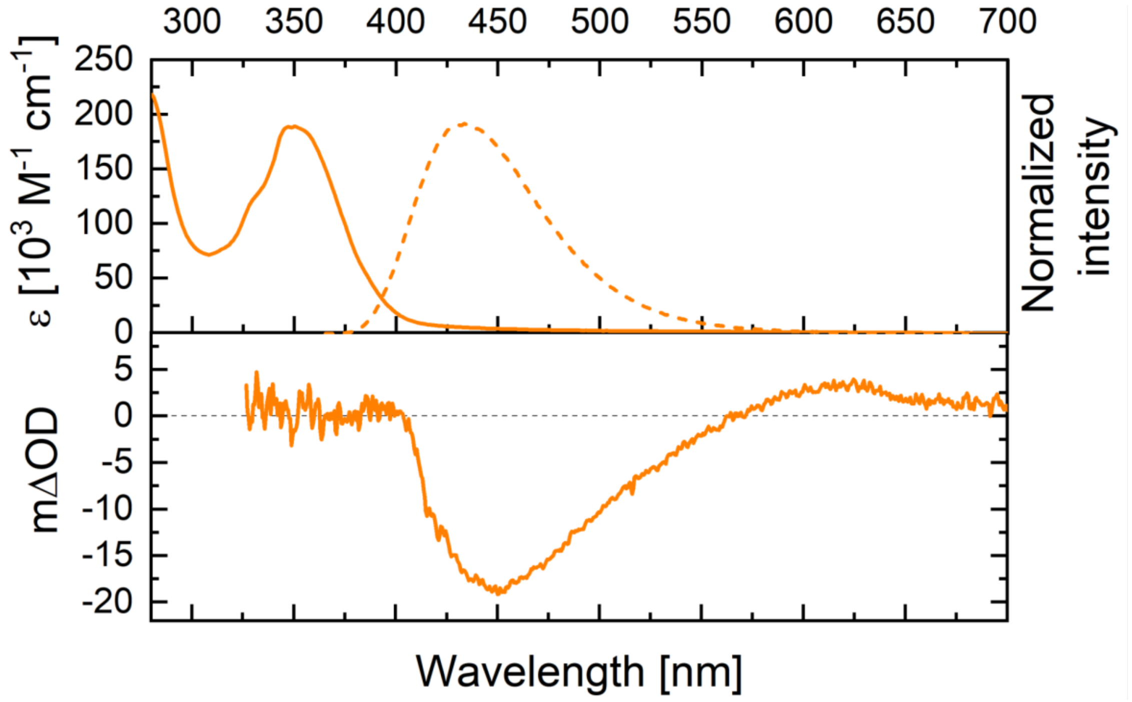

Figure S72. UV–Vis absorption (top, solid), emission (top, dashed) and transient absorption spectra (bottom) of [$FeCl_2$] in deaerated solutions of THF at 20 °C. The transient absorption signal was time-integrated for 2 ns immediately after the laser pulse. Excitation occurred at 410 nm. A bleach at 450 nm originating from stimulated emission (SE) and an excited state absorption (ESA) at 610 nm are observed.

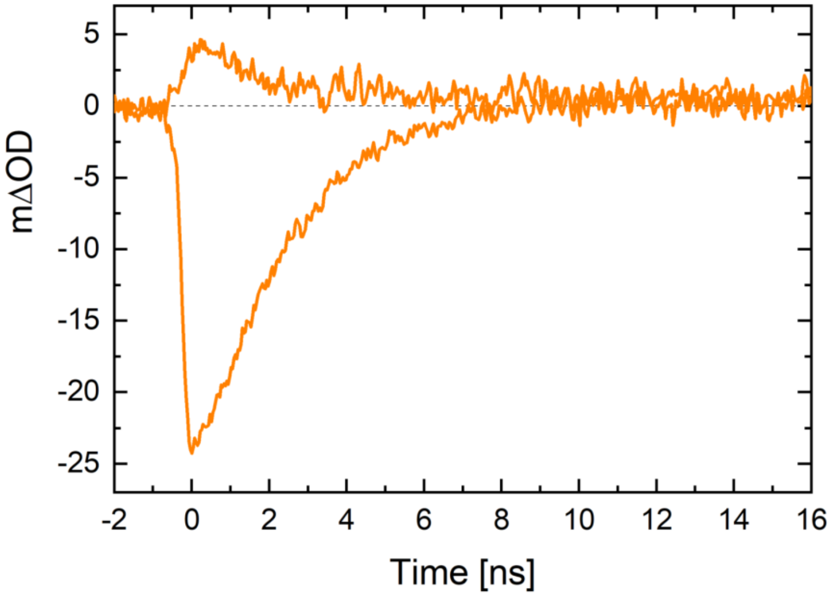

Figure S73. Ground state recoveries at 450 nm (SE) and 610 nm (ESA) of [$FeCl_2$] in deaerated THF at 20 °C. Excitation occurred at 410 nm.

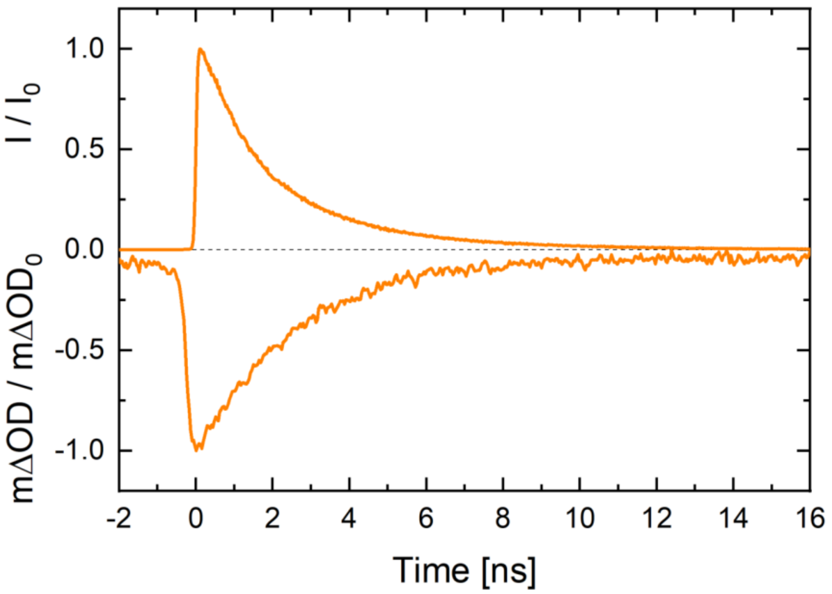

Figure S74. Ground state recovery at 450 nm and emission decay at 433 nm of [$FeCl_2$] in deaerated THF. Excitation occurred at 410 and 375 nm, respectively.

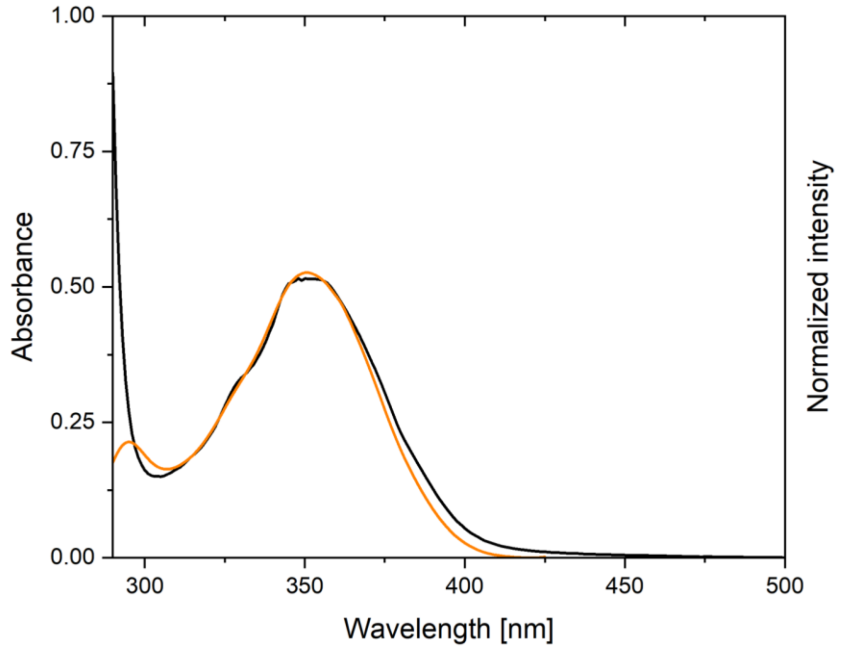

Figure S75. UV–Vis absorption (black) and excitation (orange) spectra of [$FeCl_2$] (λ_{det} = 435 nm) in THF at 20 °C.

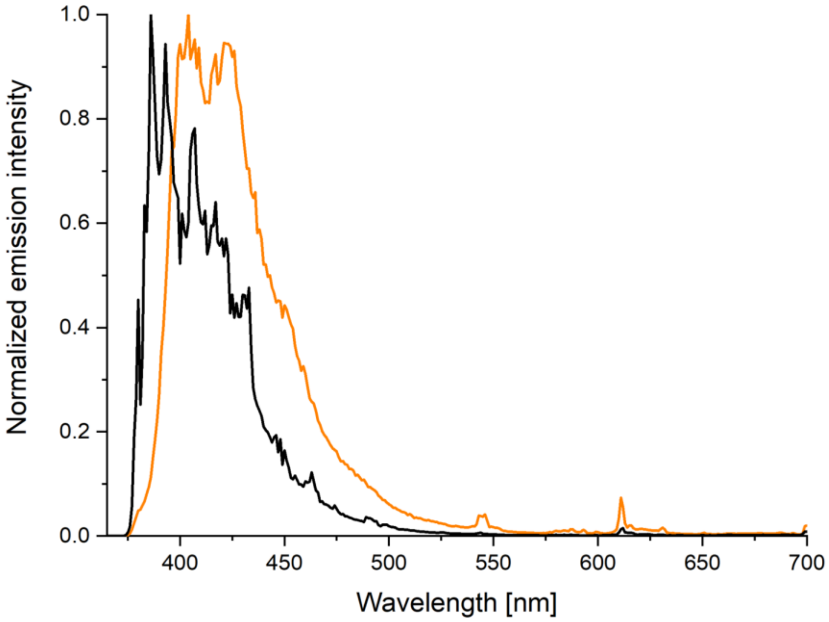

Figure S76. Emission spectra of L^{Pyr} (black) and [$FeCl_2$] (orange) in 2-Me-THF at 77 K. Excitation occurred at 350 nm.

9. Quantum chemistry

9.1 Computational Details

All quantum chemical calculations to assess structural and electronic properties of the three complexes [Cr], $[Mn]^+$ and $[Fe]^{2+}$ were carried out using the Gaussian 16 program.[20] In the calculations, the *tert*-butyl groups positioned in the α-position of the coordinating isocyanides were simplified to methyl groups. Such an overall structural simplification of the transition metal complexes was done to decrease the computational demand of the already sizable systems (around 320 atoms) without affecting the investigated spectroscopic and excited state properties. Noteworthy, such simplification would have a pronounced effect on the fully relaxed ^{3}MC equilibrium structures as shown lately by the consortium.[21] Such electronic states were, however, not considered for optimization due to the size of the investigated complexes in the present study. Density functional theory (DFT) was employed to obtain fully relaxed ground state geometries of all three complexes within the singlet ground state (S_0) as well as within the lowest triplet state (T_1). To this aim, the B3LYP XC functional[22–24] was applied in combination with the all-electron def2-SVP basis set[25,26]. Additionally, dispersion correction was included using the GD3 model with Becke-Johnson damping.[27] Implicit solvent effects (tetrahydrofuran, $\varepsilon = 7.4257$) were taken into account by the polarizable continuum model (PCM) using equilibrium procedure of the SMD solvation model.[28,29] The vibrational frequency calculations showed that all obtained geometries are minima of the potential energy surface. All optimized structures are available via the free online repository Zenodo.[30]

Subsequently, time-dependent DFT (TDDFT) was applied to assess the singlet and triplet excited states properties (i.e. electronic characters, energies and oscillator strengths). Therefore, the same computation setup was employed as in the initial ground state calculations. The excited state properties within the Franck-Condon point (S_0 equilibrium structure) were evaluated by means of the non-equilibrium procedure of solvation to estimate the initial vertical absorption energies. The 300 lowest energy singlet-singlet excitations were obtained to model the electronic absorption spectra of [Cr], $[Mn]^+$ and $[Fe]^{2+}$. In addition, the 300 lowest energy (dipole-forbidden) singlet-triplet transitions were evaluated in order to address the availability of prominent triplet states involved in the subsequent intersystem crossing (ISC) and excited state relaxation pathways. Unfortunately, the singlet-triplet population transfer could not be investigated by mean of scalar-relativistic TDDFT (including spin-orbit couplings) due to the size of the transition metal complexes and the large number of singlet and triplet states underlying the experimental excitation wavelengths of 550 nm and, in particular, at 370 nm excitation. Furthermore, the Franck-Condon photophysics of [Cr], $[Mn]^+$ and $[Fe]^{2+}$ were assessed by means of the MN15 XC functional[31] and the range-separated CAM-B3LYP functional[32]. However, the B3LYP functional provides overall a better description

of the dipole-allowed transitions as governed by the ^{1}MLCT, ^{1}LMCT, ^{1}ILCT and ^{1}IL states of interest. Therefore, we will focus in the further data analysis and discussion on the B3LYP results.

Furthermore, the transition absorption (TA) spectra of the three complexes were simulated, where the excited-state absorption (ESA) was modelled by means of the lowest 500 spin and dipole-allowed triplet-triplet transitions as obtained within the previously optimized T_1 equilibria. Assuming a 1:1 population of S_0 and T_1, the ESAs are given by the triplet-triplet excitation and the ground state bleach (GSB) by the singlet-singlet excitations within the singlet ground state equilibrium for [Cr], $[Mn]^+$ and $[Fe]^{2+}$, respectively. This approach allows to reliably model the TA signal upon excitation of the longest wavelength absorption band at long delay times.[21,33,34]

In addition, emission – or more precisely – phosphorescence energies were calculated based on the ΔSCF approach.[35] Thereby, the emission energy from the thermally equilibrated lowest triplet state is modeled as the energy gap between the fully relaxed triplet ground state (T_1) and the singlet ground state within the same structure. Thus, the ΔSCF approach relies exclusively on (open-shell and closed-shell) DFT simulations, while the equilibrium model of solvation was applied for T_1.

All optimized structures (S_0 and T_1) as obtained at the B3LYP level of theory as well as high-resolution images of charge density differences are available from the online repository Zenodo via Ref. 30 (https://zenodo.org/records/8246834). In addition, key molecular orbitals (high-resolution images and cube files) are provided exemplarily for [Cr] within its fully equilibrated singlet ground state structure.

The following nomenclature is used for the assignment of excited states;

$^{1/3}$MLCT$_L$: Singlet/triplet MLCT transitions solely including the *m*-terphenyl part of the ligand framework.

$^{1/3}$MLCT$_{Pyr}$: Singlet/triplet MLCT transitions where pyrene moiety(s) contribute.

$^{1/3}$IL$_{Pyr}$: Singlet/triplet intra-ligand transitions to the pyrene moiety(s)

$^{1/3}$ILCT: Intra-ligand charge transfer transition

$^{1/3}$LMCT: Singlet/triplet transitions to the pyrene moiety(s) (No subscript is used as the electron position is often more important than the hole).

$^{1/3}$LLCT$_{Pyr}$: Singlet/triplet inter-ligand or ligand-to-ligand charge transfer transitions to the pyrene moiety(s) (only appears in the SI)

9.2 Franck-Condon Photophysics

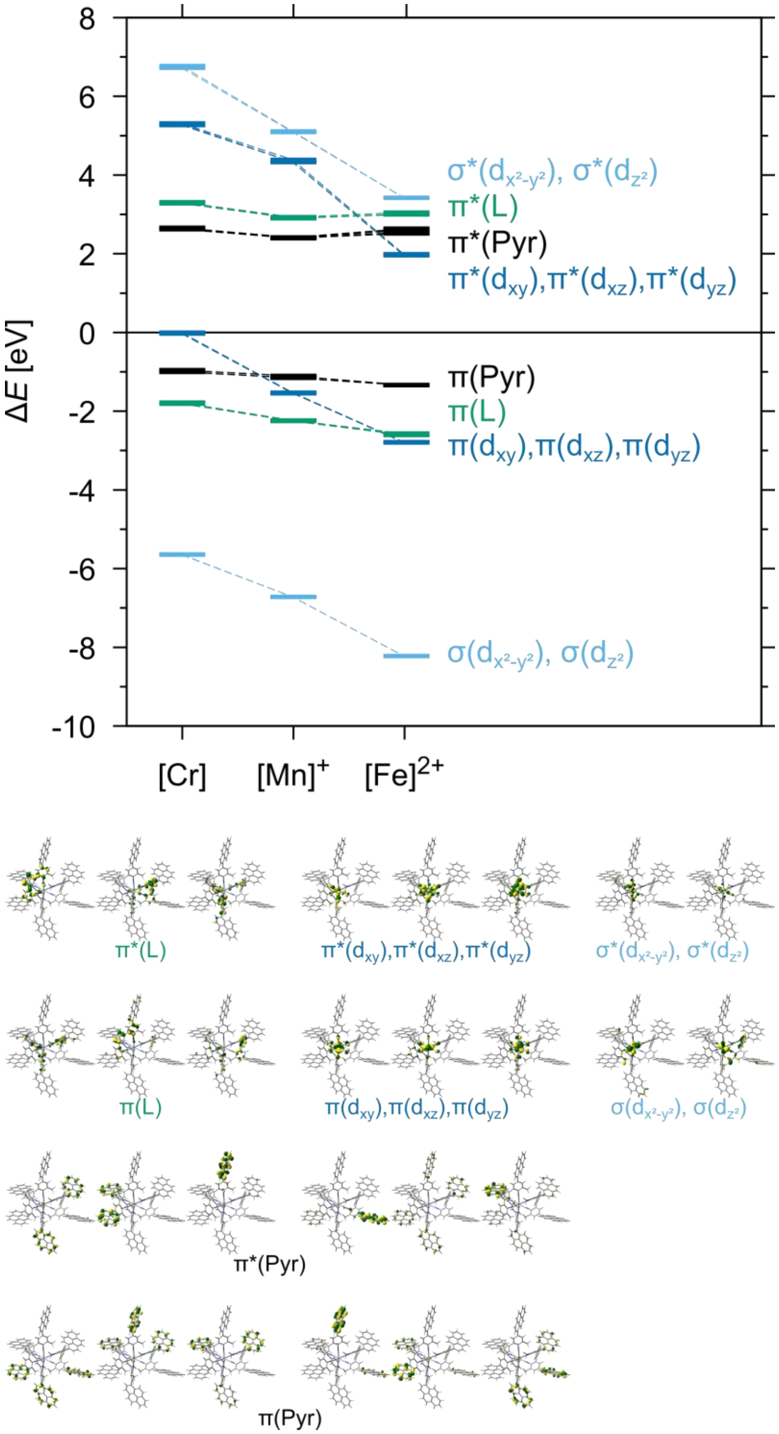

Figure S77. Frontier orbitals energy diagram for [Cr], $[Mn]^{+}$ and $[Fe]^{2+}$ obtained at the B3LYP/def2-SVP level of theory (in THF). Orbitals are shown exemplarily for [Cr]; rendered orbitals are collected in cube-format under Ref. 30 (https://zenodo.org/records/8246834) The t_{2g} orbitals ($\pi(d_{xy})$, $\pi(d_{xz})$, $\pi(d_{yz})$) are stabilized by 1.5 and 2.8 eV for $[Mn]^{+}$ and $[Fe]^{2+}$ relative to [Cr], respectively.

Table S14. Relative molecular orbital energies for [Cr], [Mn] and $[Fe]^{2+}$ in eV as obtained within the fully equilibrated singlet ground state. The color coding matches that of Figure S77. High-resolution images are provided exemplarily for [Cr] by means of the online repository Zenodo via Ref. 30 (https://zenodo.org/records/8246834). Indicated MO numbers refer to the respective molecular orbitals of the [Cr] reference system.

MO	[Cr]	$[Mn]^{+}$	$[Fe]^{2+}$
π(d_{ij}), 565-567	0.0000	-1.5181	-2.7674
	0.0030	-1.5151	-2.7581
	0.0278	-1.4977	-2.7524
π*(d_{ij}), 610-612	5.2959	4.3541	1.9878
	5.3209	4.3579	1.9951
	5.3432	4.4164	2.0210
σ(d_{ij}), 458, 459	-5.6186	-6.6935	-8.1916
	-5.6104	-6.6907	-8.1903
σ*(d_{ij}), 637, 638	6.7547	5.1215	3.4480
	6.8001	5.1391	3.4526
π(Pyr), 559-564	-0.9799	-1.1274	-1.3151
	-0.9772	-1.1255	-1.3140
	-0.9679	-1.1219	-1.3121
	-0.9328	-1.0781	-1.3102
	-0.9279	-1.0762	-1.3102
	-0.9255	-1.0732	-1.3026
π*(Pyr), 568-573	2.6520	2.4213	2.5418
	2.6591	2.4341	2.5519
	2.6615	2.4349	2.5731
	2.6860	2.4395	2.6207
	2.6877	2.4417	2.6428
	2.6890	2.4428	2.6670
π(L), 556-558	-1.7864	-2.2248	-2.5788
	-1.7530	-2.2101	-2.5660
	-1.7508	-2.2017	-2.5331
π*(L), 574-576	3.3067	2.9263	3.0247
	3.3171	2.9435	3.0359
	3.3429	2.9603	3.0773

Table S15. Simulated IR-active CN vibrational normal modes for [Cr], $[Mn]^+$ and $[Fe]^{2+}$ within the fully equilibrated S_0 and T_1 structures. All frequencies were scaled by a factor of 0.95 to account for the approximate treatment of electron correlation and the applied harmonic approximation.[36,37]

**Singlet ground state (S_0) equilibrium structure**						
	**[Cr]**		**$[Mn]^+$**		**$[Fe]^{2+}$**	
Mode	Wavenumber [cm^{-1}]	IR intensity [km/mol]	Wavenumber [cm^{-1}]	IR intensity [km/mol]	Wavenumber [cm^{-1}]	IR intensity [km/mol]
ν(CN1, 688)	1942	17376	2050	6959	2147	1414
ν(CN2, 689)	1942	16741	2051	7027	2147	1485
ν(CN3, 690)	1943	12733	2052	5145	2148	1032
ν(CN4, 691)	1970	254	2064	174	2151	61
ν(CN5, 692)	1971	244	2064	245	2152	86
ν(CN6, 693)	2092	11	2148	3	2190	1
**Lowest triplet state (T_1) equilibrium structure**						
	**[Cr] ($^3MLCT_{Pyr}$)**		**$[Mn]^+$ ($^3IL_{Pyr}$)**		**$[Fe]^{2+}$ ($^3IL_{Pyr}$)**	
Mode	Wavenumber [cm^{-1}]	IR intensity [km/mol]	Wavenumber [cm^{-1}]	IR intensity [km/mol]	Wavenumber [cm^{-1}]	IR intensity [km/mol]
ν(CN1, 688)	1962	308	2044	7019	2130	102
ν(CN2, 689)	2008	4097	2050	6972	2146	1399
ν(CN3, 690)	2016	7127	2051	5620	2146	1177
ν(CN4, 691)	2024	6330	2062	699	2148	662
ν(CN5, 692)	2035	278	2064	218	2151	139
ν(CN6, 693)	2100	1853	2145	92	2185	287

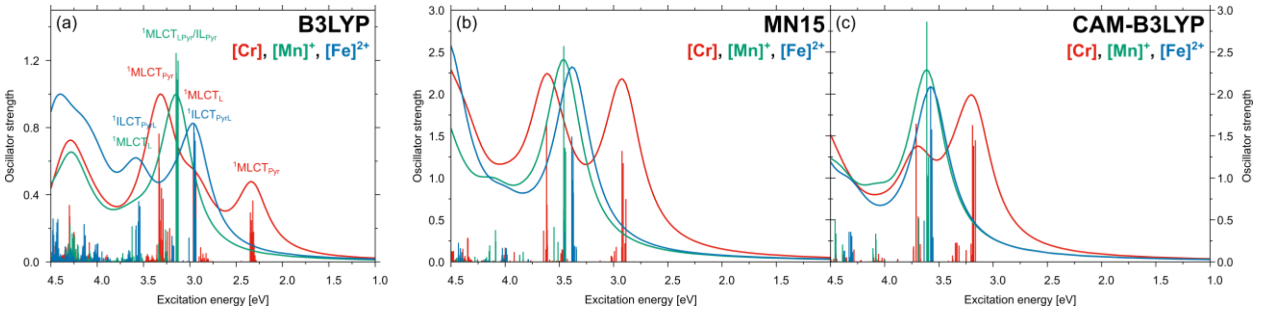

Figure S78. Simulated electronic absorption spectra of [Cr], $[Mn]^+$ and $[Fe]^{2+}$ obtained at the (a) B3LYP, (b) MN15 and (c) CAM-B3LYP level of theory in conjunction with the def2-SVP basis set (in THF). The spectra are visualized in an energy unit (eV) to allow an improved comparability.

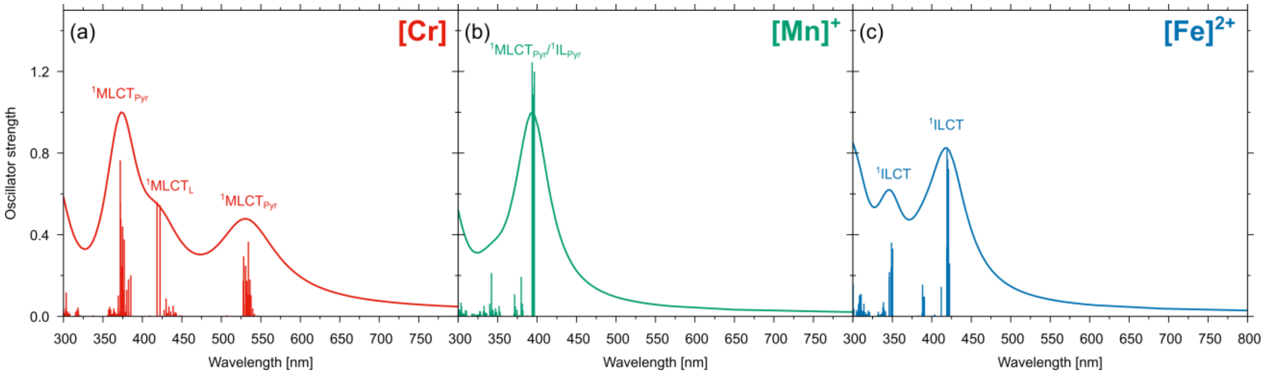

Figure S79. Simulated electronic absorption spectra of (a) [Cr], (b) $[Mn]^+$ and (b) $[Fe]^{2+}$ obtained at the B3LYP/def2-SVP level of theory (in THF). Characters of key transitions are indicated.

Table S16. Simulated Franck-Condon physics of [Cr] as obtained by the B3LYP functional. Prominent dipole-allowed singlet-singlet transitions contributing the UV-Vis absorption (left) and spin-forbidden singlet-triplet transitions (right), *i.e.*, low-lying metal-to-ligand charge transfer states ($^3MLCT_{Pyr}$) involving the LUMOs of the pyrene moieties, low-lying pyrene-based intraligand states ($^3IL_{Pyr}$) and low-lying metal-centered states (^{3}MC; $\pi\left(d_{t_{2g}}\right)/\pi^*\left(d_{t_{2g}}\right)$) are summarized.

Excitation $S_0 \rightarrow S_i$	Character	ΔE [eV]	λ [nm]	f	Excitation $S_0 \rightarrow T_i$	Character	ΔE [eV]	λ [nm]
S_6	$MLCT_{Pyr}$	2.31	538	0.1034	T_1	$MLCT_{Pyr}$	1.85	669
S_7	$MLCT_{Pyr}$	2.31	536	0.1807	T_2	IL_{Pyr}	1.99	624
S_9	$MLCT_{Pyr}$	2.32	535	0.1079	T_3	IL_{Pyr}	1.99	623
S_{10}	$MLCT_{Pyr}$	2.32	534	0.3659	T_4	IL_{Pyr}	1.99	622
S_{11}	$MLCT_{Pyr}$	2.33	533	0.1069	T_5	IL_{Pyr}	2.00	621
S_{12}	$MLCT_{Pyr}$	2.33	532	0.1739	T_6	IL_{Pyr}	2.00	620
S_{13}	$MLCT_{Pyr}$	2.33	531	0.1594	T_7	IL_{Pyr}	2.06	601
S_{14}	$MLCT_{Pyr}$	2.34	531	0.2483	T_8	$MLCT_{Pyr}$	2.18	570
S_{16}	$MLCT_{Pyr}$	2.35	528	0.2946	T_9	$MLCT_{Pyr}$	2.18	569
S_{17}	$MLCT_{Pyr}$	2.35	528	0.1726	T_{10}	$MLCT_{Pyr}$	2.19	567
S_{34}	$MLCT_L$	2.94	422	0.5446	T_{11}	$MLCT_{Pyr}$	2.20	564
S_{35}	$MLCT_L$	2.96	419	0.5601	T_{12}	$MLCT_{Pyr}$	2.20	563
S_{37}	$MLCT_L$	3.22	385	0.2014	T_{13}	$MLCT_{Pyr}$	2.26	549
S_{39}	$MLCT_L$	3.24	382	0.1814	T_{14}	$MLCT_{Pyr}$	2.27	547
S_{40}	$MLCT_L$	3.26	380	0.1297	T_{15}	$MLCT_{Pyr}$	2.28	544
S_{43}	$MLCT_{Pyr}$	3.29	377	0.3766	T_{16}	$MLCT_{Pyr}$	2.29	542
S_{44}	$MLCT_{Pyr}$	3.29	376	0.1296	T_{17}	$MLCT_{Pyr}$	2.29	541
S_{46}	$MLCT_{Pyr}$	3.31	375	0.4406	T_{18}	$MLCT_{Pyr}$	2.29	540
S_{48}	$MLCT_{Pyr}$	3.32	373	0.1364	T_{19}	$MLCT_{Pyr}$	2.30	539
S_{49}	$MLCT_{Pyr}$	3.32	373	0.2470	T_{20}	$MLCT_{Pyr}$	2.30	538
S_{50}	$MLCT_{Pyr}$	3.32	373	0.1281	T_{21}	$MLCT_{Pyr}$	2.31	536
S_{51}	$MLCT_{Pyr}$	3.33	373	0.4761	T_{22}	$MLCT_{Pyr}$	2.33	533
S_{52}	$MLCT_{Pyr}$	3.33	372	0.5613	T_{23}	$MLCT_{Pyr}$	2.33	533
S_{53}	$MLCT_{Pyr}$	3.33	372	0.7645	T_{24}	$MLCT_{Pyr}$	2.33	532
S_{58}	$MLCT_{Pyr}$	3.36	370	0.1022	T_{137}	MC	3.69	336
					T_{138}	MC	3.69	336
					T_{139}	MC	3.76	330
					T_{140}	MC	3.76	329
					T_{141}	MC	3.77	329

Table S17. Simulated Franck-Condon physics of $[Mn]^+$ as obtained by the B3LYP functional. Prominent dipole-allowed singlet-singlet transitions contributing the UV-Vis absorption (left) and spin-forbidden singlet-triplet transitions (right), *i.e.*, low-lying metal-to-ligand charge transfer states ($^3MLCT_{Pyr}$) involving the LUMOs of the pyrene moieties, low-lying pyrene-based intraligand states ($^3IL_{Pyr}$) and low-lying metal-centered states (^{3}MC; $\pi\left(d_{t_{2g}}\right)/\pi^*\left(d_{t_{2g}}\right)$ and $\left(d_{t_{2g}}\right)/\sigma^*\left(d_{e_g}\right)$) are summarized.

Excitation $S_0 \rightarrow S_i$	Character	ΔE [eV]	λ [nm]	f	Excitation $S_0 \rightarrow T_i$	Character	ΔE [eV]	λ [nm]
S_1	$IL_{Pyr}/MLCT_{Pyr}$	3.13	397	1.1996	T_1	$IL_{Pyr}/MLCT_{Pyr}$	2.03	611
S_2	$IL_{Pyr}/MLCT_{Pyr}$	3.13	396	1.0863	T_2	$IL_{Pyr}/MLCT_{Pyr}$	2.03	611
S_3	$IL_{Pyr}/MLCT_{Pyr}$	3.14	395	0.7697	T_3	$IL_{Pyr}/MLCT_{Pyr}$	2.03	611
S_4	$IL_{Pyr}/MLCT_{Pyr}$	3.15	394	1.1262	T_4	$IL_{Pyr}/MLCT_{Pyr}$	2.03	610
S_5	$IL_{Pyr}/MLCT_{Pyr}$	3.15	394	1.2448	T_5	$IL_{Pyr}/MLCT_{Pyr}$	2.03	610
S_{13}	$IL_{Pyr}/MLCT_{Pyr}$	3.26	380	0.1932	T_6	$IL_{Pyr}/MLCT_{Pyr}$	2.03	610
S_{57}	$MLCT_L$	3.62	342	0.2126	T_7	$MLCT_L$/MC	2.55	486
					T_8	$MLCT_L$	2.85	435
					T_9	$MLCT_L$	2.85	435
					T_{10}	$MLCT_L$	2.89	429
					T_{11}	$MLCT_L$	2.90	428
					T_{12}	$MLCT_L$	2.90	428
					T_{13}	$MLCT_{Pyr}$	3.10	399
					T_{14}	$MLCT_{Pyr}$	3.11	399
					T_{15}	Mixed	3.13	396
					T_{16}	$MLCT_{Pyr}$/MC	3.17	391
					T_{17}	$MLCT_{Pyr}/LLCT_{Pyr}$	3.19	389
					T_{18}	$MLCT_{Pyr}/LLCT_{Pyr}$	3.19	388
					T_{19}	$MLCT_{Pyr}/LLCT_{Pyr}$	3.20	387
					T_{20}	$MLCT_{Pyr}/LLCT_{Pyr}$	3.21	386
					T_{21}	$MLCT_{Pyr}/LLCT_{Pyr}$	3.21	386
					T_{22}	$MLCT_{Pyr}/LLCT_{Pyr}$	3.27	379
					T_{23}	$MLCT_{Pyr}/LLCT_{Pyr}$	3.27	379
					T_{24}	$MLCT_{Pyr}/LLCT_{Pyr}$	3.28	378
					T_{124}	MC	3.87	321
					T_{125}	MC	3.87	320
					T_{129}	$MC/LLCT_{Pyr}$	3.90	318
					T_{135}	$MC/LLCT_{Pyr}$	3.94	314

Table S18. Simulated Franck-Condon physics of $[Fe]^{2+}$ as obtained by the B3LYP functional. Prominent dipole-allowed singlet-singlet transitions contributing the UV-Vis absorption (left) and spin-forbidden singlet-triplet transitions (right), *i.e.*, low-lying metal-to-ligand charge transfer states ($^3MLCT_{Pyr}$) involving the LUMOs of the pyrene moieties, low-lying pyrene-based intraligand states ($^3IL_{Pyr}$) and low-lying metal-centered states (^3MC; $\pi\left(d_{t_{2g}}\right)/\pi^*\left(d_{t_{2g}}\right)$ and $\left(d_{t_{2g}}\right)/\sigma^*\left(d_{e_g}\right)$) are summarized.

Excitation $S_0 \rightarrow S_i$	Character	ΔE [eV]	λ [nm]	f	Excitation $S_0 \rightarrow T_i$	Character	ΔE [eV]	λ [nm]
S_1	ILCT/LMCT	2.93	422	0.2592	T_1	IL_{Pyr}	2.01	616
S_2	ILCT/LMCT	2.94	421	0.7223	T_2	IL_{Pyr}	2.02	615
S_3	ILCT/LMCT	2.95	420	0.7701	T_3	IL_{Pyr}	2.02	615
S_4	ILCT/LMCT	2.96	419	0.8159	T_4	IL_{Pyr}	2.02	615
S_5	ILCT/LMCT	2.96	419	0.3411	T_5	IL_{Pyr}	2.02	614
S_6	ILCT/LMCT	3.01	412	0.1446	T_6	IL_{Pyr}	2.02	613
S_{19}	LLCT	3.17	391	0.0951	T_7	ILCT/LMCT	2.86	434
S_{20}	ILCT	3.19	389	0.0992	T_8	ILCT/LMCT	2.87	432
S_{22}	ILCT	3.19	388	0.1251	T_9	ILCT/LMCT	2.88	430
S_{23}	ILCT	3.19	388	0.1550	T_{10}	ILCT/LMCT	2.89	429
S_{37}	ILCT	3.54	350	0.3316	T_{11}	ILCT/LMCT	2.89	428
S_{38}	ILCT/LMCT	3.55	349	0.3616	T_{12}	ILCT/LMCT	2.90	428
S_{39}	ILCT	3.55	349	0.0938	T_{13}	LLCT/LMCT	3.07	404
S_{40}	ILCT	3.56	349	0.2400	T_{14}	LLCT/LMCT	3.07	404
S_{41}	ILCT/LMCT	3.58	346	0.2169	T_{15}	LLCT/LMCT	3.07	404
S_{42}	ILCT/LMCT	3.58	346	0.1918	T_{16}	LLCT/LMCT	3.07	403
					T_{17}	LLCT/LMCT	3.07	403
					T_{18}	LLCT/LMCT	3.08	403
					T_{19}	LLCT/LMCT	3.08	402
					T_{20}	LLCT/LMCT	3.09	401
					T_{21}	LLCT/LMCT	3.10	400
					T_{22}	LLCT/LMCT	3.11	399
					T_{23}	LLCT/LMCT	3.12	398
					T_{24}	LLCT/LMCT	3.11	398
					T_{46}	MC	3.33	373
					T_{47}	MC	3.33	372
					T_{48}	ILCT/MC	3.35	370
					T_{49}	ILCT/MC	3.35	370

Table S19. Electronic characters – as visualized by charge density differences (CDDs) – of prominent singlet-singlet ($S_0 \rightarrow S_i$) and singlet-triplet ($S_0 \rightarrow T_i$) excitations within the Franck-Condon geometry (i.e., S_0 equilibrium) of [Cr] as obtained by the B3LYP functional. Charge transfer takes place from red to blue.[30]

**singlet-singlet ($S_0 \rightarrow S_i$) excitations**			
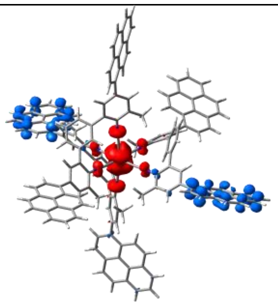 S_6 ($MLCT_{Pyr}$)	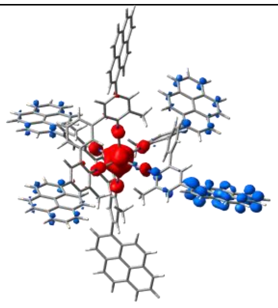 S_7 ($MLCT_{Pyr}$)	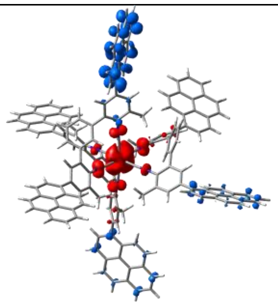 S_9 ($MLCT_{Pyr}$)	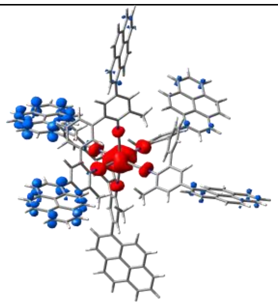 S_{10} ($MLCT_{Pyr}$)
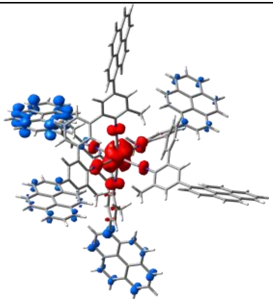 S_{11} ($MLCT_{Pyr}$)	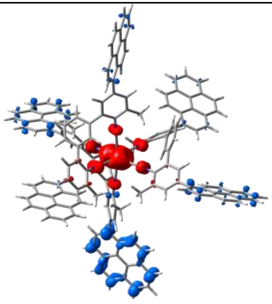 S_{12} ($MLCT_{Pyr}$)	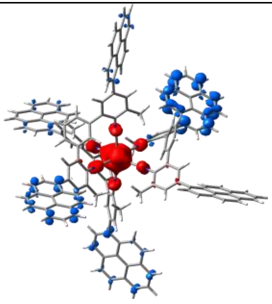 S_{13} ($MLCT_{Pyr}$)	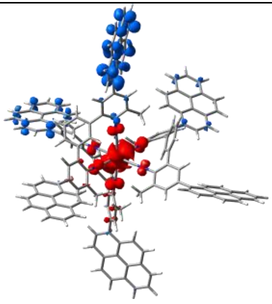 S_{14} ($MLCT_{Pyr}$)
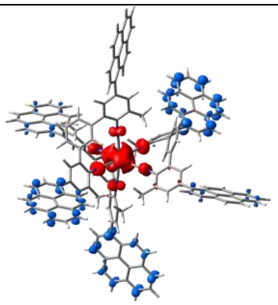 S_{16} ($MLCT_{Pyr}$)	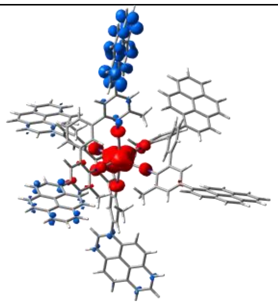 S_{17} ($MLCT_{Pyr}$)	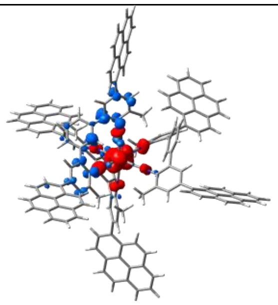 S_{34} ($MLCT_L$)	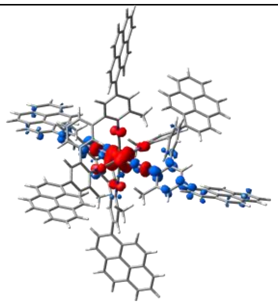 S_{35} ($MLCT_L$)
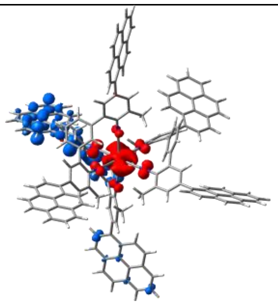 S_{37} ($MLCT_L$)	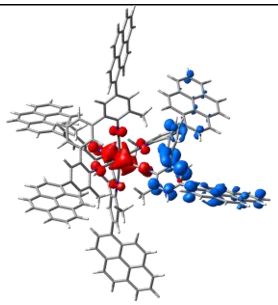 S_{39} ($MLCT_L$)	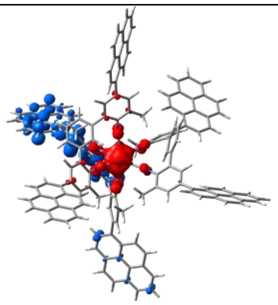 S_{40} ($MLCT_L$)	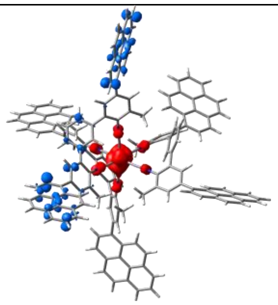 S_{43} ($MLCT_{Pyr}$)

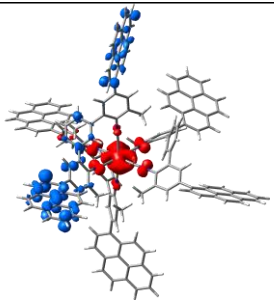 S_{44} ($MLCT_{Pyr}$)	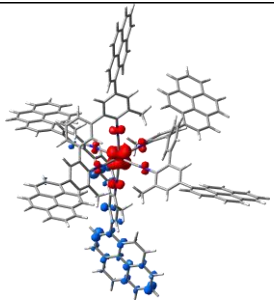 S_{46} ($MLCT_{Pyr}$)	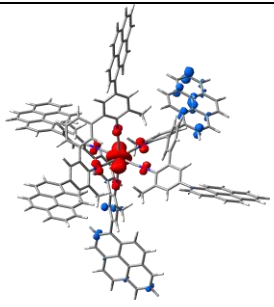 S_{48} ($MLCT_{Pyr}$)	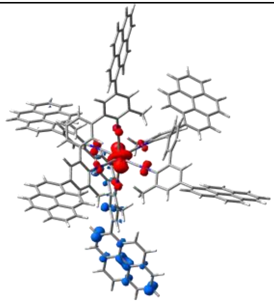 S_{49} ($MLCT_{Pyr}$)
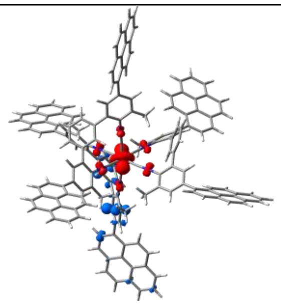 S_{50} ($MLCT_{Pyr}$)	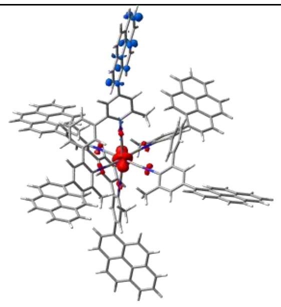 S_{51} ($MLCT_{Pyr}$)	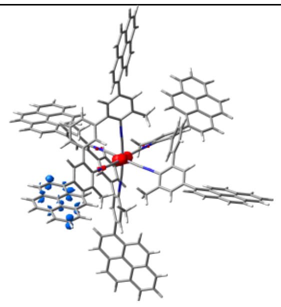 S_{52} ($MLCT_{Pyr}$)	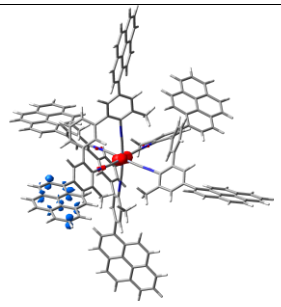 S_{53} ($MLCT_{Pyr}$)
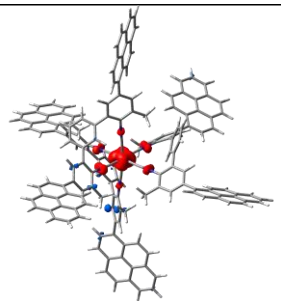 S_{58} ($MLCT_{Pyr}$)			
**singlet-triplet ($S_0 \rightarrow T_i$) excitations**			
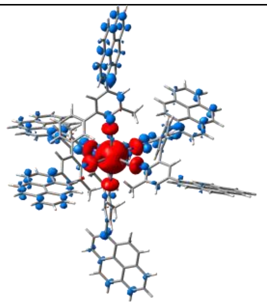 T_1 (IL_{Pyr}/$MLCT_{Pyr}$)	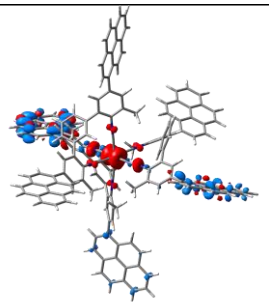 T_2 (IL_{Pyr}/$MLCT_{Pyr}$)	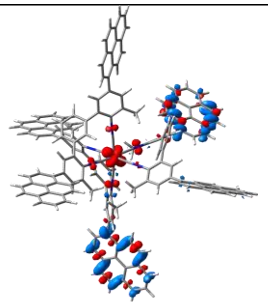 T_3 (IL_{Pyr}/$MLCT_{Pyr}$)	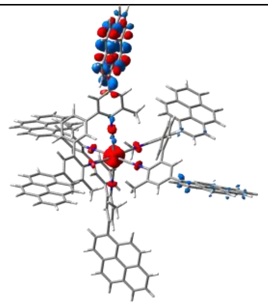 T_4 (IL_{Pyr}/$MLCT_{Pyr}$)

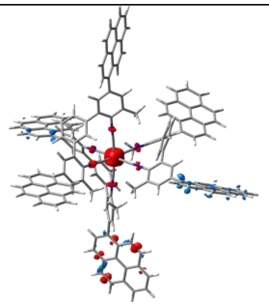

T_5 (IL_{Pyr}/$MLCT_{Pyr}$)

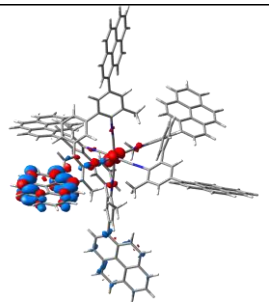

T_6 (IL_{Pyr}/$MLCT_{Pyr}$)

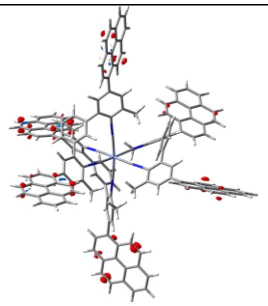

T_7 (IL_{Pyr})

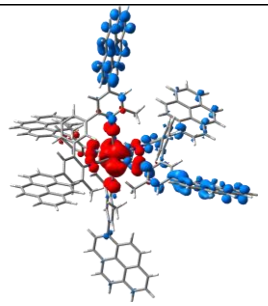

T_8 ($MLCT_{Pyr}$)

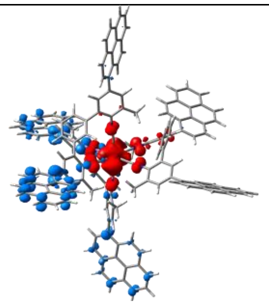

T_9 ($MLCT_{Pyr}$)

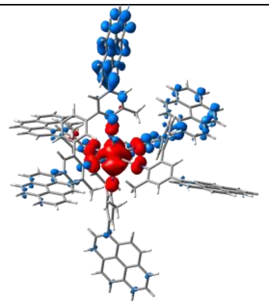

T_{10} ($MLCT_{Pyr}$)

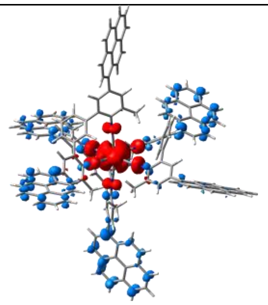

T_{11} ($MLCT_{Pyr}$)

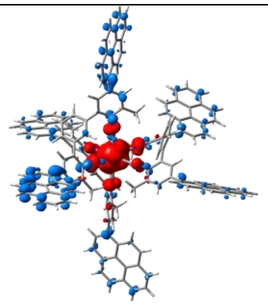

T_{12} ($MLCT_{Pyr}$)

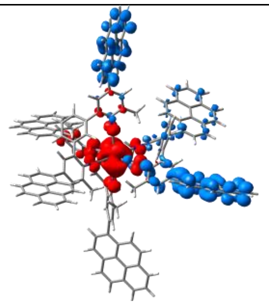

T_{13} ($MLCT_{Pyr}$)

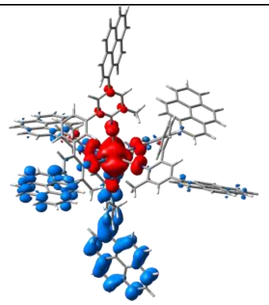

T_{14} ($MLCT_{Pyr}$)

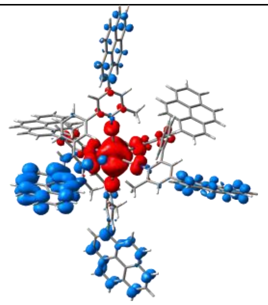

T_{15} ($MLCT_{Pyr}$)

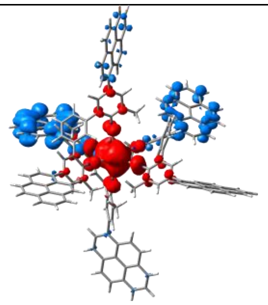

T_{16} ($MLCT_{Pyr}$)

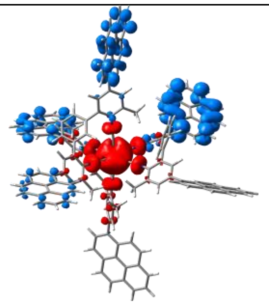

T_{17} ($MLCT_{Pyr}$)

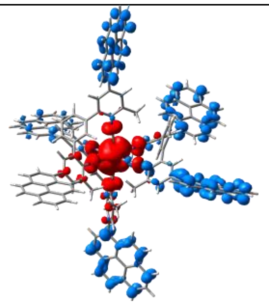

T_{18} ($MLCT_{Pyr}$)

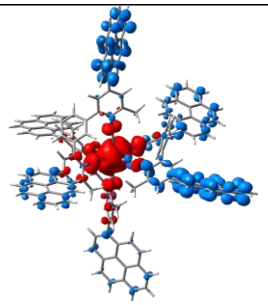

T_{19} ($MLCT_{Pyr}$)

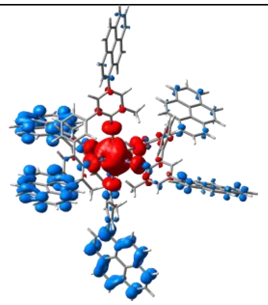

T_{20} ($MLCT_{Pyr}$)

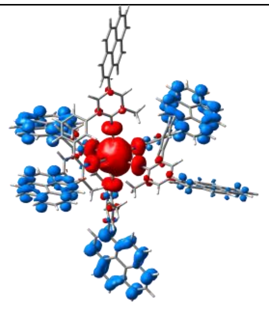

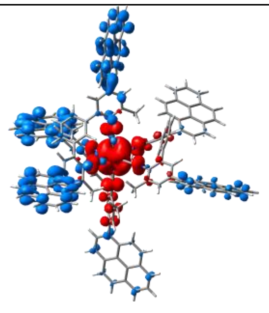

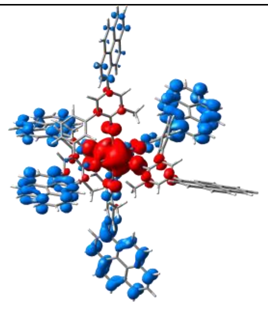

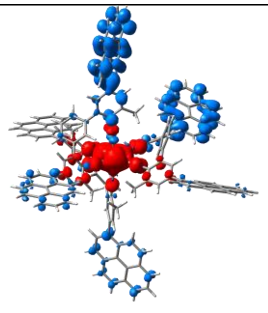

T_{21} ($MLCT_{Pyr}$)	T_{22} ($MLCT_{Pyr}$)	T_{23} ($MLCT_{Pyr}$)	T_{24} ($MLCT_{Pyr}$)
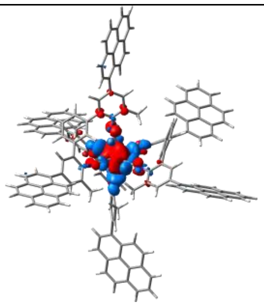<br>T_{137} (MC)	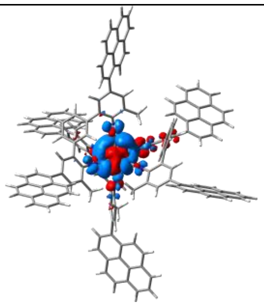<br>T_{138} (MC)	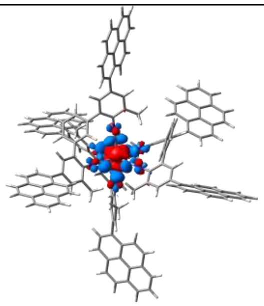<br>T_{139} (MC)	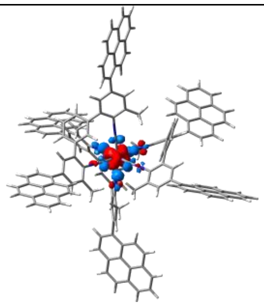<br>T_{140} (MC)
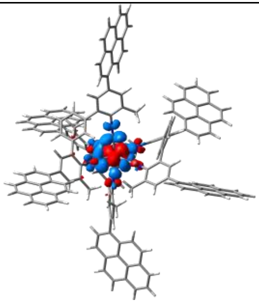<br>T_{141} (MC)			

Table S20. Electronic characters – as visualized by charge density differences (CDDs) – of prominent singlet-singlet ($S_0 \rightarrow S_i$) and singlet-triplet ($S_0 \rightarrow T_i$) excitations within the Franck-Condon geometry (i.e., S_0 equilibrium) of $[Mn]^+$ as obtained by the B3LYP functional. Charge transfer takes place from red to blue.[30]

**singlet-singlet ($S_0 \rightarrow S_i$) excitations**			
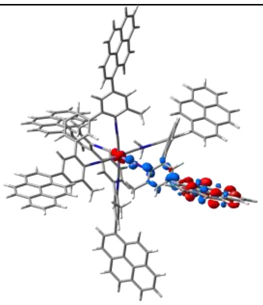<br>S_1 (IL_{Pyr}/$MLCT_{Pyr}$)	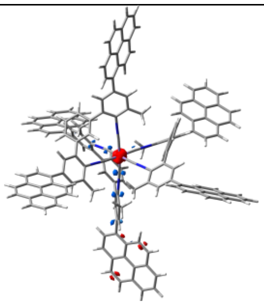<br>S_2 (IL_{Pyr}/$MLCT_{Pyr}$)	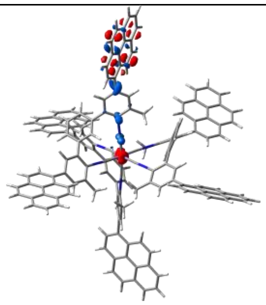<br>S_3 (IL_{Pyr}/$MLCT_{Pyr}$)	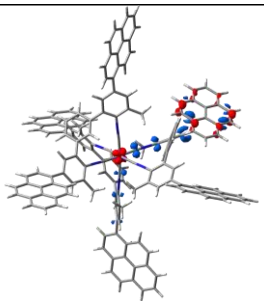<br>S_4 (IL_{Pyr}/$MLCT_{Pyr}$)

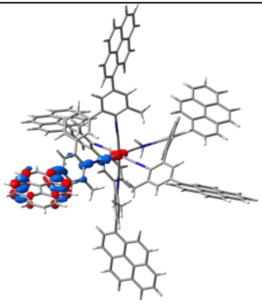<br>S_5 (IL_{Pyr}/$MLCT_{Pyr}$)	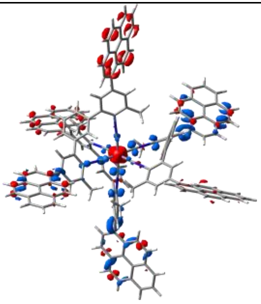<br>S_{13} (IL_{Pyr}/$MLCT_{Pyr}$)	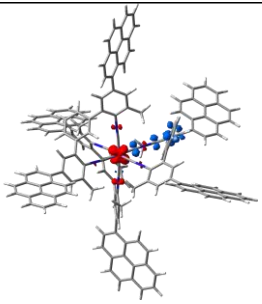<br>S_{57} ($MLCT_L$)	
**singlet-triplet ($S_0 \rightarrow T_i$) excitations**			
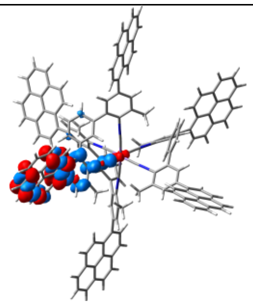<br>T_1 ($MLCT_{Pyr}$)	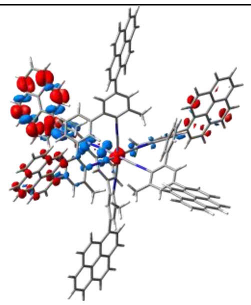<br>T_2 (IL_{Pyr})	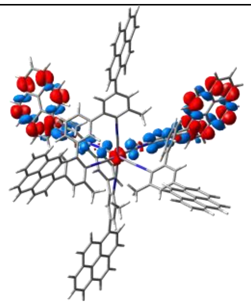<br>T_3 (IL_{Pyr})	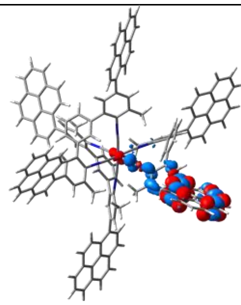<br>T_4 (IL_{Pyr})
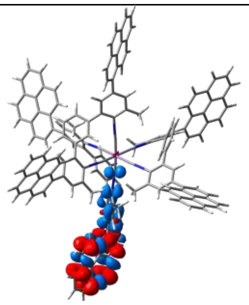<br>T_5 (IL_{Pyr})	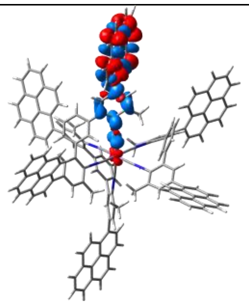<br>T_6 (IL_{Pyr})	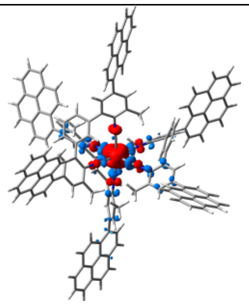<br>T_7 ($MLCT_L$/MC)	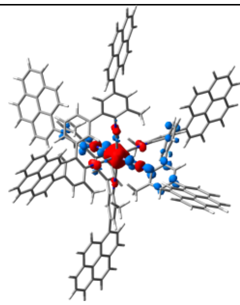<br>T_8 ($MLCT_L$)
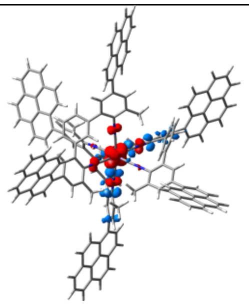<br>T_9 ($MLCT_L$)	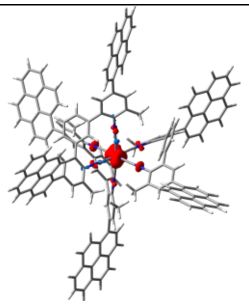<br>T_{10} ($MLCT_L$)	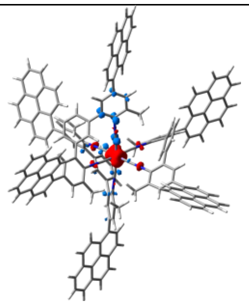<br>T_{11} ($MLCT_L$)	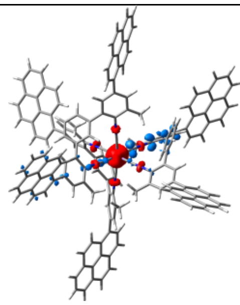<br>T_{12} ($MLCT_L$)

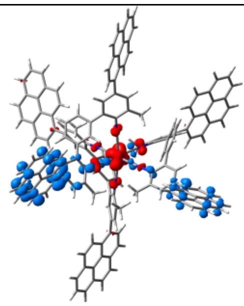 T_{13} ($MLCT_{Pyr}$)	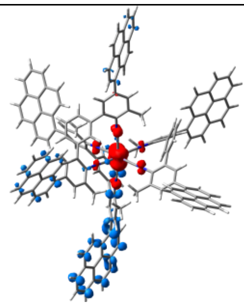 T_{14} ($MLCT_{Pyr}$)	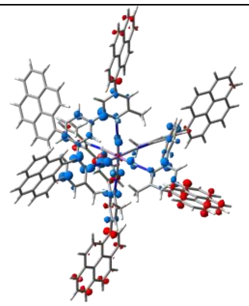 T_{15} (Mixed)	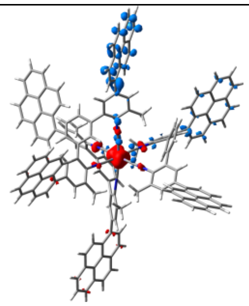 T_{16} ($MLCT_{Pyr}$/MC)
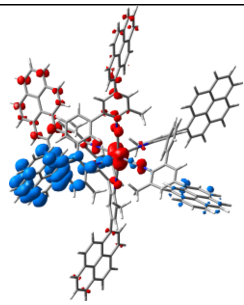 T_{17} ($MLCT_{Pyr}$/$LLCT_{Pyr}$)	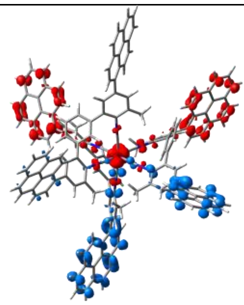 T_{18} ($MLCT_{Pyr}$/$LLCT_{Pyr}$)	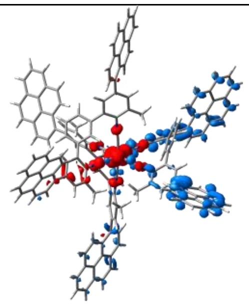 T_{19} ($MLCT_{Pyr}$/$LLCT_{Pyr}$)	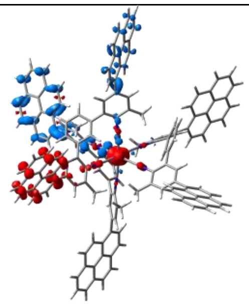 T_{20} ($MLCT_{Pyr}$/$LLCT_{Pyr}$)
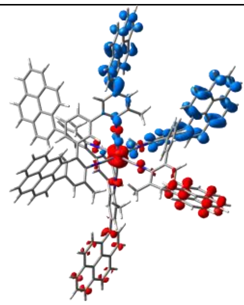 T_{21} ($MLCT_{Pyr}$/$LLCT_{Pyr}$)	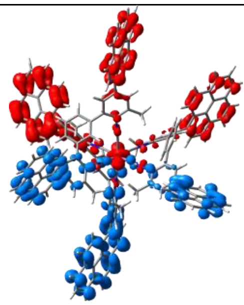 T_{22} ($MLCT_{Pyr}$/$LLCT_{Pyr}$)	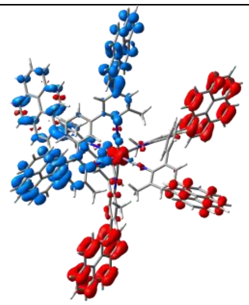 T_{23} ($MLCT_{Pyr}$/$LLCT_{Pyr}$)	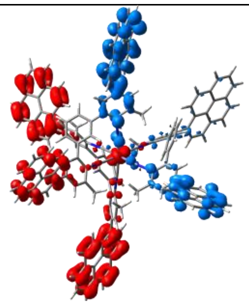 T_{24} ($MLCT_{Pyr}$/$LLCT_{Pyr}$)
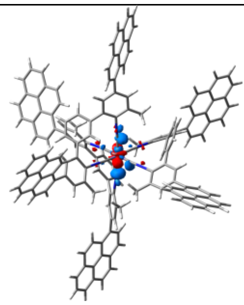 T_{124} (MC)	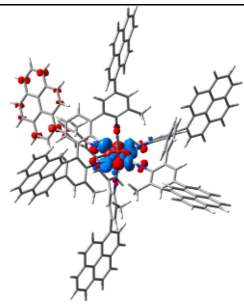 T_{125} (MC)	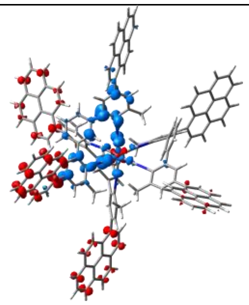 T_{129} (MC/$LLCT_{Pyr}$)	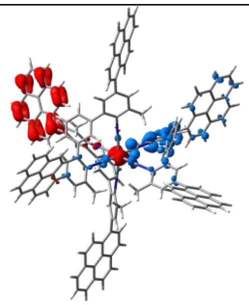 T_{135} (MC/$LLCT_{Pyr}$)

Table S21. Electronic characters – as visualized by charge density differences (CDDs) – of prominent singlet-singlet ($S_0 \rightarrow S_i$) and singlet-triplet ($S_0 \rightarrow T_i$) excitations within the Franck-Condon geometry (i.e., S_0 equilibrium) of $[Fe]^{2+}$ as obtained by the B3LYP functional. Charge transfer takes place from red to blue.[30]

**singlet-singlet ($S_0 \rightarrow S_i$) excitations**			
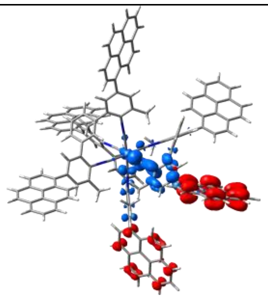 S_1 (ILCT/LMCT)	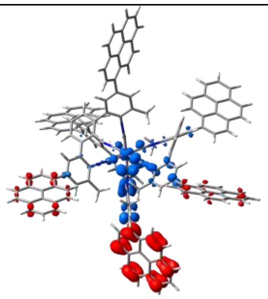 S_2 (ILCT/LMCT)	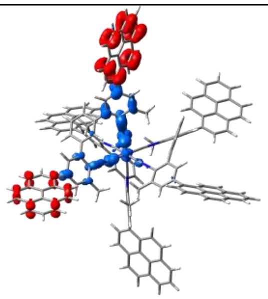 S_3 (ILCT/LMCT)	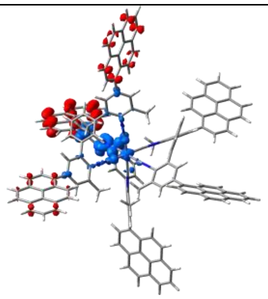 S_4 (ILCT/LMCT)
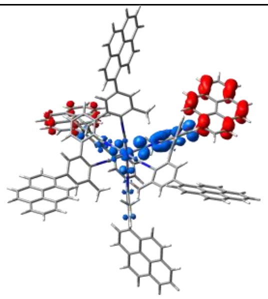 S_5 (ILCT/LMCT)	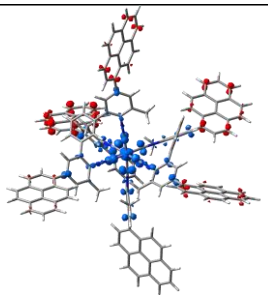 S_6 (ILCT/LMCT)	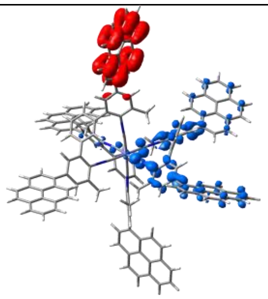 S_{19} (LLCT)	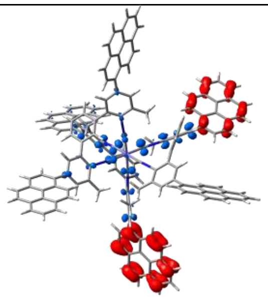 S_{20} (ILCT)
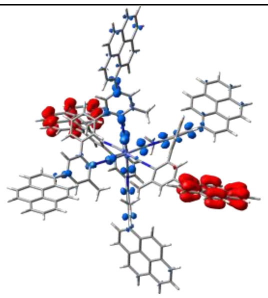 S_{22} (ILCT)	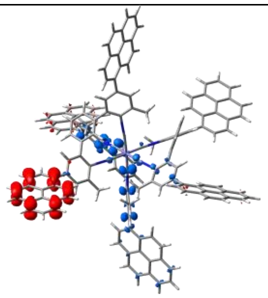 S_{23} (ILCT)	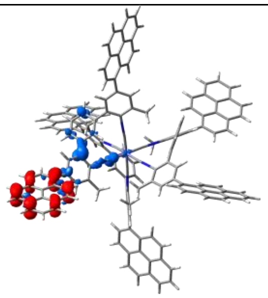 S_{37} (ILCT)	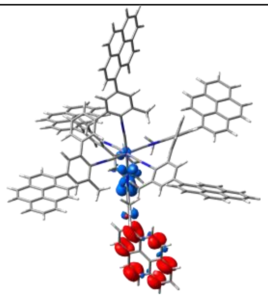 S_{38} (ILCT/LMCT)
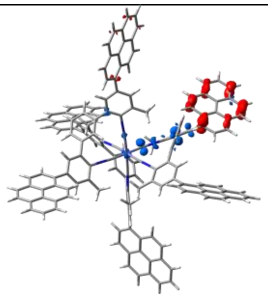 S_{39} (ILCT)	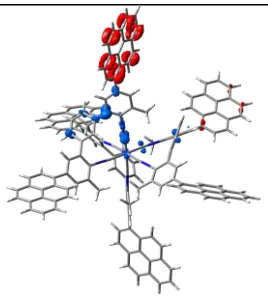 S_{40} (ILCT)	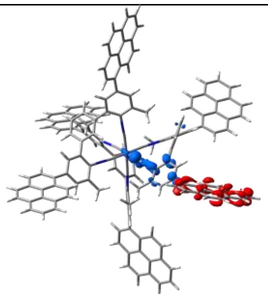 S_{41} (ILCT/LMCT)	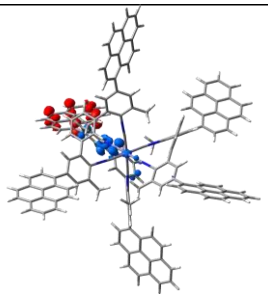 S_{42} (ILCT/LMCT)

**singlet-triplet ($S_0 \rightarrow T_i$) excitations**			
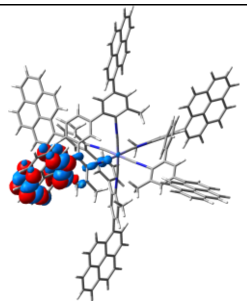<br>T_1 (IL_{Pyr})	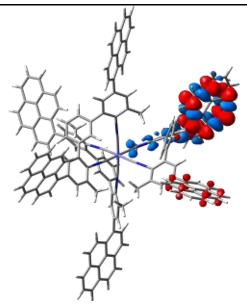<br>T_2 (IL_{Pyr})	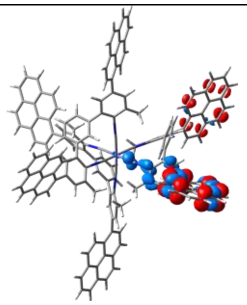<br>T_3 (IL_{Pyr})	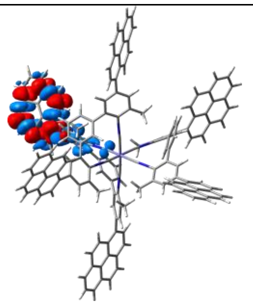<br>T_4 (IL_{Pyr})
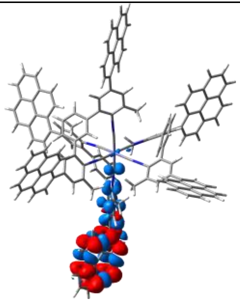<br>T_5 (IL_{Pyr})	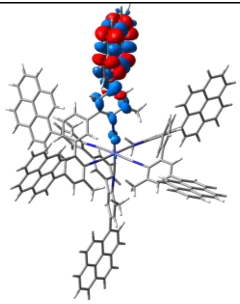<br>T_6 (IL_{Pyr})	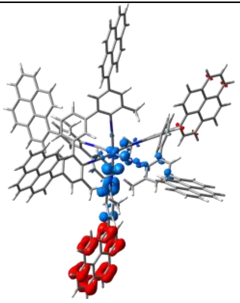<br>T_7 (ILCT/LMCT)	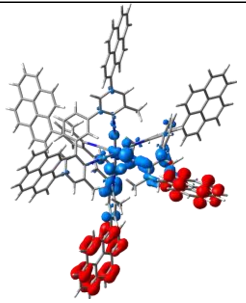<br>T_8 (ILCT/LMCT)
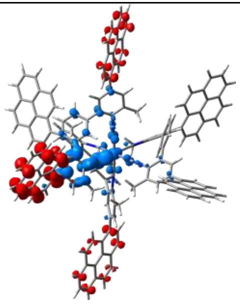<br>T_9 (ILCT/LMCT)	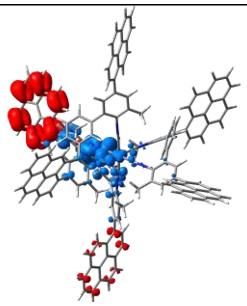<br>T_{10} (ILCT/LMCT)	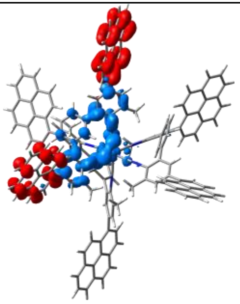<br>T_{11} (ILCT/LMCT)	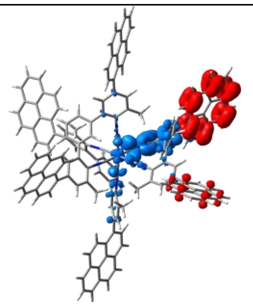<br>T_{12} (ILCT/LMCT)
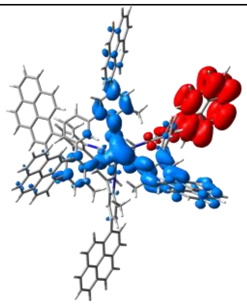<br>T_{13} (LLCT/LMCT)	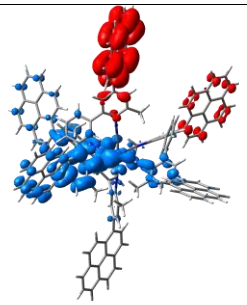<br>T_{14} (LLCT/LMCT)	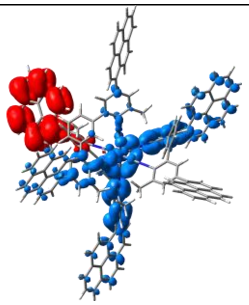<br>T_{15} (LLCT/LMCT)	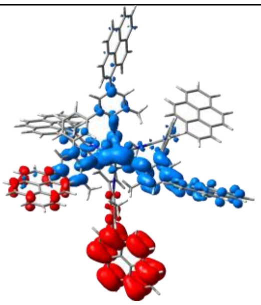<br>T_{16} (LLCT/LMCT)

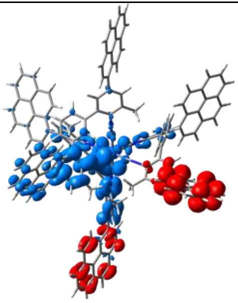	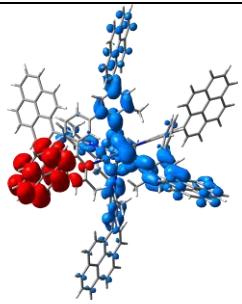	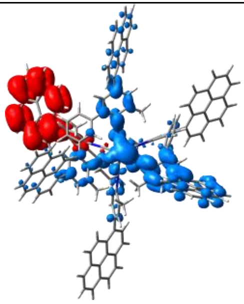	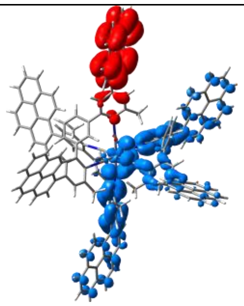
T_{17} (LLCT/LMCT)	T_{18} (LLCT/LMCT)	T_{19} (LLCT/LMCT)	T_{20} (LLCT/LMCT)
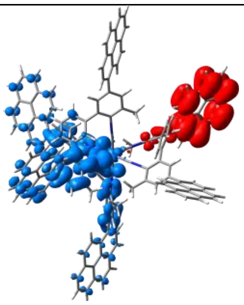	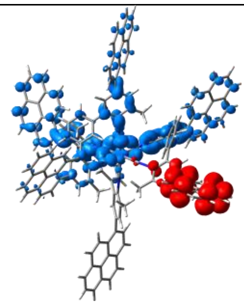	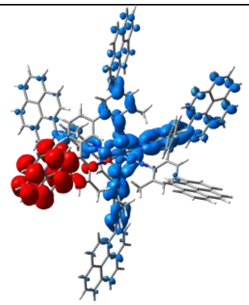	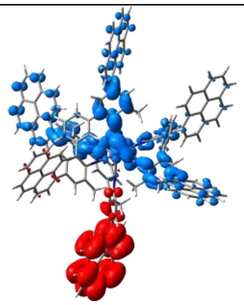
T_{21} (LLCT/LMCT)	T_{22} (LLCT/LMCT)	T_{23} (LLCT/LMCT)	T_{24} (LLCT/LMCT)
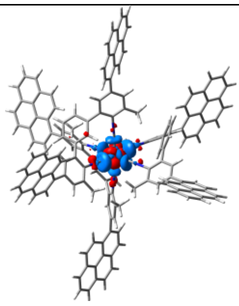	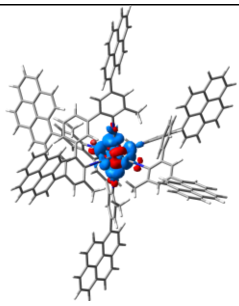	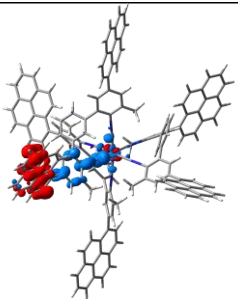	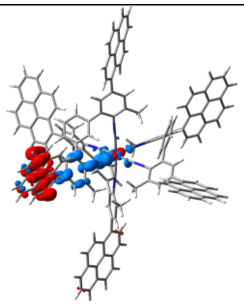
T_{46} (MC)	T_{47} (MC)	T_{48} (ILCT/MC)	T_{49} (ILCT/MC)

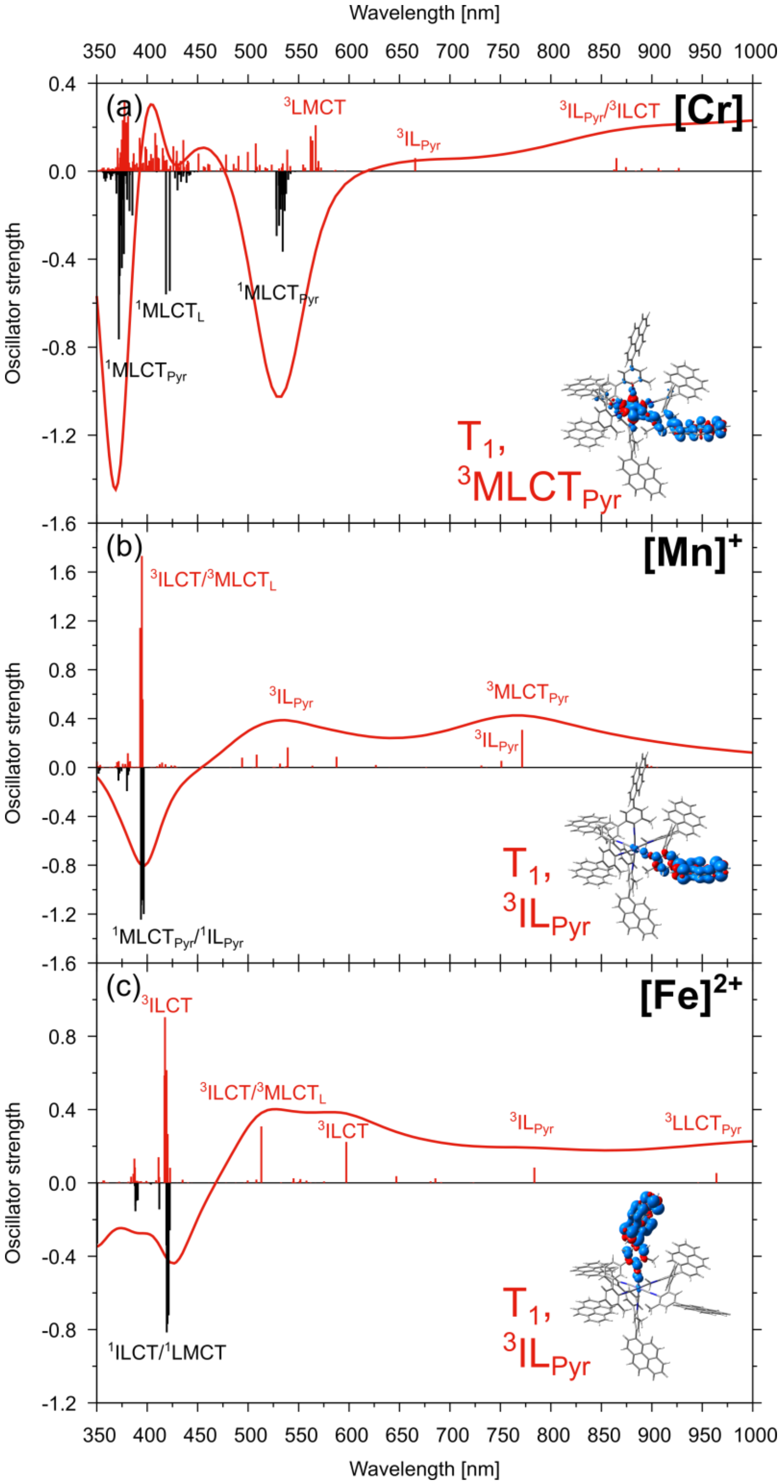

Figure S80. Simulated transient absorption spectra of (a) [Cr], (b) $[Mn]^{+}$ and (b) $[Fe]^{2+}$ obtained at the B3LYP/def2-SVP level of theory (in THF). Excited-state absorption is estimated by spin and dipole-allowed triplet-triplet transitions (in red) as obtained within the fully relaxed T_1 structure. Contributions of ground-state bleach are given by the dipole-allowed singlet-singlet transitions at the Franck-Condon point. Characters of the lowest triplet state (T_1) are indicated as spin density insets.

Table S22. Simulated excited-state absorption of [Cr] as obtained by the B3LYP functional. Prominent spin and dipole-allowed triplet-triplet transitions contributing the TA-UV-Vis are summarized. DFT-optimized triplet ground state (T_1, ^{3}MLCT) is calculated to 1.56 eV (λ_{em} = 793 nm) above the S_0 in its optimized geometry.

Excitation $T_1 \rightarrow T_i$	Character	ΔE [eV]	λ [nm]	f	$\langle s^2 \rangle$
T_{14}	IL_{Pyr}/ILCT	0.96	1285	0.3421	2.04
T_{25}	IL_{Pyr}/ILCT	1.43	865	0.0600	2.05
T_{28}	IL_{Pyr}	1.86	666	0.0598	2.05
T_{46}	LMCT	2.19	567	0.2090	2.38
T_{47}	LMCT	2.20	564	0.1395	2.40
T_{48}	LMCT	2.20	562	0.1595	2.56
T_{52}	LMCT	2.30	539	0.0981	2.57
T_{61}	$MLCT_{Pyr}$	2.44	508	0.1268	3.33
T_{88}	$MLCT_{Pyr}$	2.85	436	0.1419	2.84
T_{105}	$MLCT_{Pyr}$	3.02	410	0.0627	2.78
T_{124}	LMCT	3.16	393	0.1519	2.75
T_{136}	LMCT	3.25	381	0.2510	2.42
T_{139}	LMCT	3.26	381	0.0809	2.71
T_{141}	LMCT	3.28	378	0.3142	2.66
T_{142}	LMCT	3.28	377	0.2607	2.60
T_{143}	LLCT	3.30	376	0.1851	2.83
T_{144}	LLCT	3.30	376	0.2327	2.47
T_{146}	LMCT	3.31	375	0.1446	2.84

Table S23. Simulated excited-state absorption of $[Mn]^{+}$ as obtained by the B3LYP functional. Prominent spin and dipole-allowed triplet-triplet transitions contributing the TA-UV-Vis are summarized. DFT-optimized triplet ground state (T_1, $^3IL_{Pyr}$) is calculated to 1.98 eV (λ_{em} = 627 nm) above the S_0 in its optimized geometry.

Excitation $T_1 \rightarrow T_i$	Character	ΔE [eV]	λ [nm]	f	$\langle s^2 \rangle$
T_{13}	$MLCT_{Pyr}$	1.61	772	0.3081	2.06
T_{14}	IL_{Pyr}	1.65	751	0.0550	2.07
T_{30}	IL_{Pyr}	2.11	588	0.0879	2.07
T_{32}	IL_{Pyr}	2.30	539	0.1639	2.07
T_{80}	ILCT/$MLCT_L$	3.13	396	0.5584	2.04
T_{81}	ILCT/$MLCT_L$	3.14	395	1.7308	2.04
T_{82}	ILCT/$MLCT_L$	3.16	394	1.0838	2.06
T_{83}	ILCT/$MLCT_L$	3.15	393	1.1427	2.04

Table S24. Simulated excited-state absorption of $[Fe]^{2+}$ as obtained by the B3LYP functional. Prominent spin and dipole-allowed triplet-triplet transitions contributing the TA-UV-Vis are summarized. DFT-optimized triplet ground state (T_1, $^3IL_{Pyr}$) is calculated to 1.95 eV (λ_{em} = 636 nm) above the S_0 in its optimized geometry.

Excitation $T_1 \rightarrow T_i$	Character	ΔE [eV]	λ [nm]	f	$\langle s^2 \rangle$
T_5	LLCT	1.29	964	0.0541	2.05
T_{13}	IL_{Pyr}	1.58	784	0.0841	2.06
T_{18}	IL_{Pyr}/LLCT	1.81	686	0.0254	2.05
T_{21}	ILCT	1.92	647	0.0367	2.08
T_{27}	ILCT	2.08	597	0.2237	2.07
T_{42}	ILCT/$MLCT_L$	2.42	513	0.3082	2.07
T_{67}	LLCT	2.93	423	0.0823	2.06
T_{71}	ILCT	2.95	420	0.2671	2.04
T_{74}	ILCT	2.96	419	0.6152	2.04
T_{75}	ILCT	2.97	418	0.9044	2.04
T_{76}	ILCT	2.97	417	0.5866	2.04

Table S25. Electronic characters of the lowest triplet state (T_1, spin density) and of prominent spin and dipole-allowed triplet-triplet ($T_1 \rightarrow T_i$, charge density differences) excitations within the fully equilibrated T_1 structure of [Cr] as obtained by the B3LYP functional. Charge transfer takes place from red to blue.[30]

**triplet-triplet ($T_1 \rightarrow T_i$) excitations**			
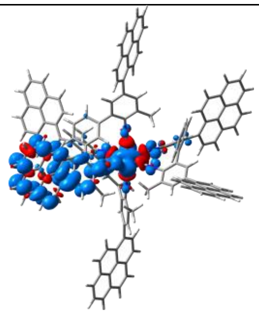<br>T_1 (MLCT$_{Pyr}$)	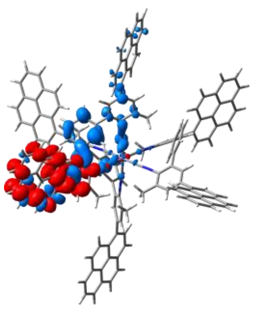<br>T_{14} (IL$_{Pyr}$/ILCT)	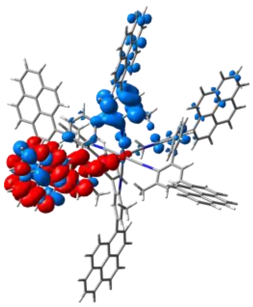<br>T_{25} (IL$_{Pyr}$/ILCT)	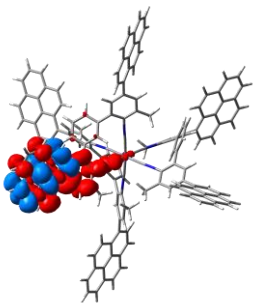<br>T_{28} (IL$_{Pyr}$)
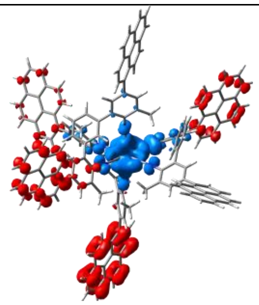<br>T_{46} (LMCT)	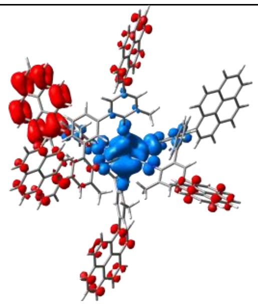<br>T_{47} (LMCT)	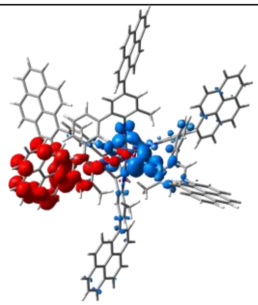<br>T_{48} (LMCT)	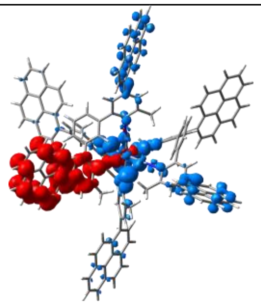<br>T_{52} (LMCT)
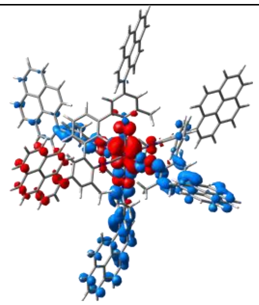<br>T_{61} (MLCT$_{Pyr}$)	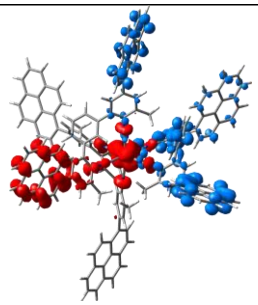<br>T_{88} (MLCT$_{Pyr}$)	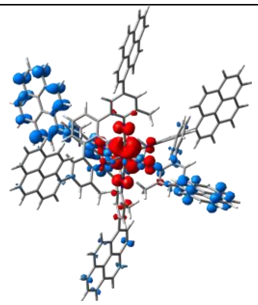<br>T_{108} (MLCT$_{Pyr}$)	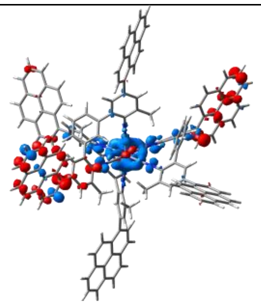<br>T_{124} (LMCT)
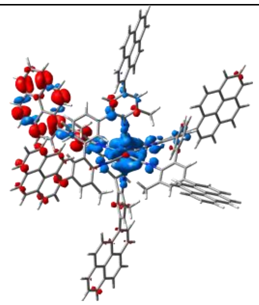<br>T_{136} (LMCT)	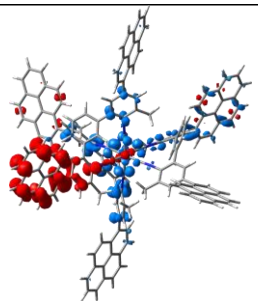<br>T_{139} (LMCT)	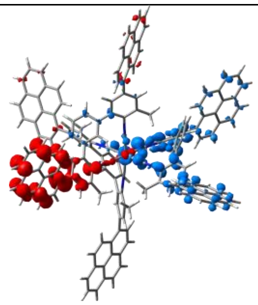<br>T_{141} (LMCT)	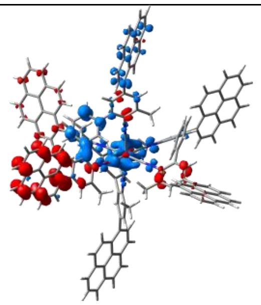<br>T_{142} (LMCT)

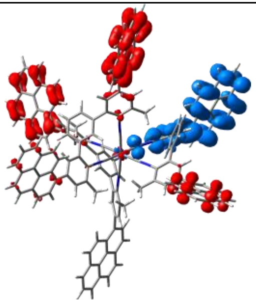<br>T_{143} (LLCT)	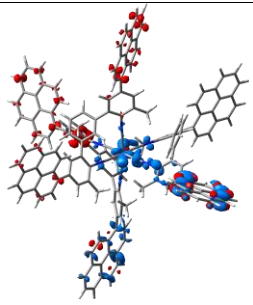<br>T_{144} (LLCT)	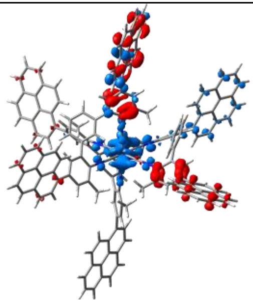<br>T_{146} (LMCT)	

Table S26. Electronic characters of the lowest triplet state (T_1, spin density) and of prominent spin and dipole-allowed triplet-triplet ($T_1 \rightarrow T_i$, charge density differences) excitations within the fully equilibrated T_1 structure of $[Mn]^+$ as obtained by the B3LYP functional. Charge transfer takes place from red to blue.[30]

**triplet-triplet ($T_1 \rightarrow T_i$) excitations**			
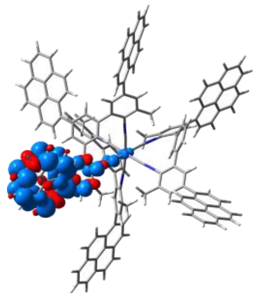<br>T_1 (IL_{Pyr})	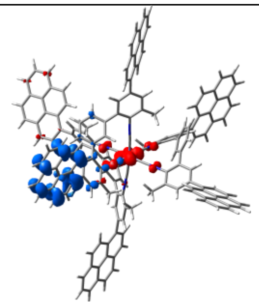<br>T_{13} ($MLCT_{Pyr}$)	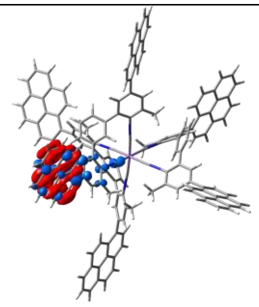<br>T_{14} (IL_{Pyr})	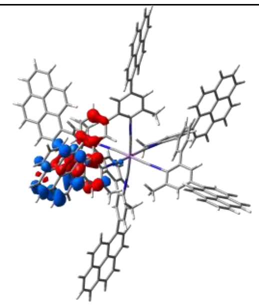<br>T_{30} (IL_{Pyr})
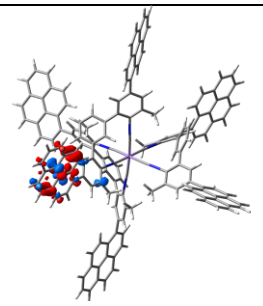<br>T_{32} (IL_{Pyr})	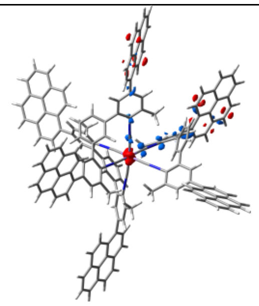<br>T_{80} (ILCT/$MLCT_L$)	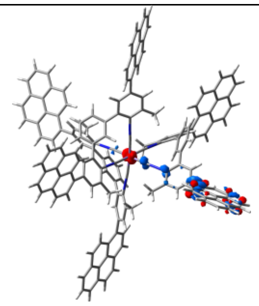<br>T_{81} (ILCT/$MLCT_L$)	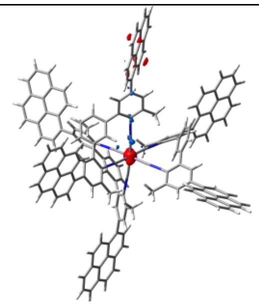<br>T_{82} (ILCT/$MLCT_L$)
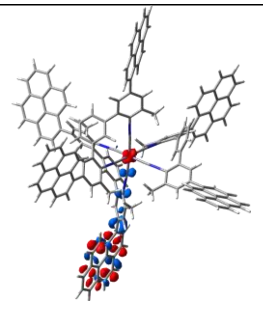<br>T_{83} (ILCT/$MLCT_L$)			

Table S27. Electronic characters of the lowest triplet state (T_1, spin density) and of prominent spin and dipole-allowed triplet-triplet ($T_1 \rightarrow T_i$, charge density differences) excitations within the fully equilibrated T_1 structure of $[Fe]^{2+}$ as obtained by the B3LYP functional. Charge transfer takes place from red to blue.[30]

**triplet-triplet ($T_1 \rightarrow T_i$) excitations**			
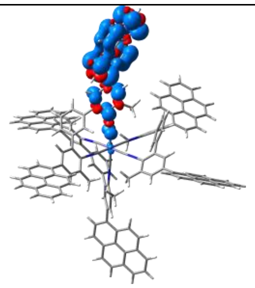<br>T_1 (IL_{Pyr})	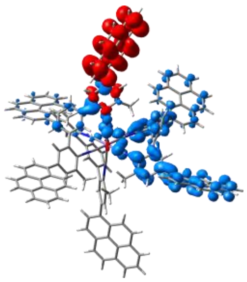<br>T_5 (LLCT)	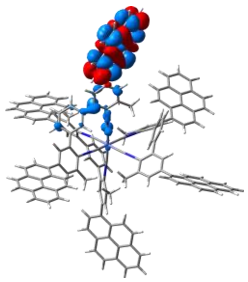<br>T_{13} (IL_{Pyr})	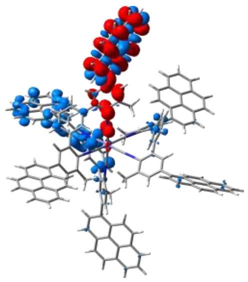<br>T_{18} (IL_{Pyr}/LLCT)
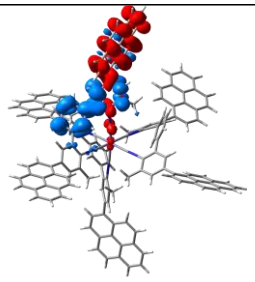<br>T_{21} (ILCT)	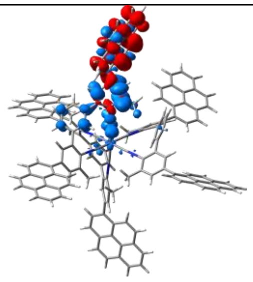<br>T_{27} (ILCT)	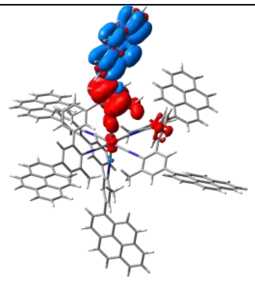<br>T_{42} (ILCT/$MLCT_L$)	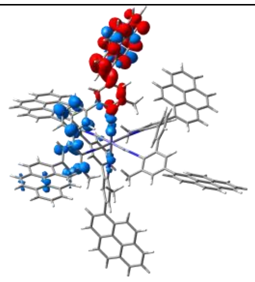<br>T_{67} (LLCT)
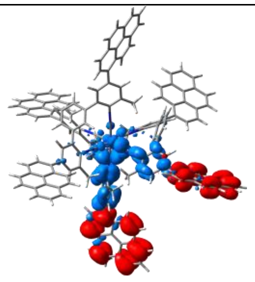<br>T_{71} (ILCT)	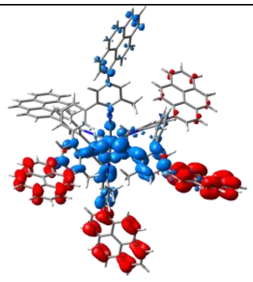<br>T_{74} (ILCT)	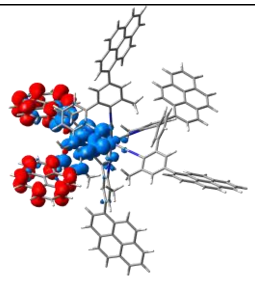<br>T_{75} (ILCT)	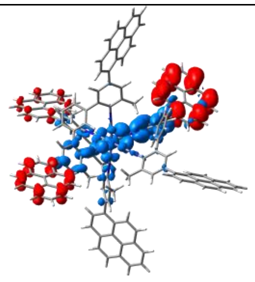<br>T_{76} (ILCT)

10. References

(1) Sinha, N.; Wegeberg, C.; Häussinger, D.; Prescimone, A.; Wenger, O. S. Photoredox-Active Cr(0) Luminophores Featuring Photophysical Properties Competitive with Ru(II) and Os(II) Complexes. *Nat. Chem.* **2023**. DOI: 10.1038/s41557-023-01297-9.

(2) Wei, Y.; Zheng, M.; Chen, L.; Zhou, X.; Liu, S. Near-Infrared to Violet Triplet-Triplet Annihilation Fluorescence Upconversion of Os(II) Complexes by Strong Spin-Forbidden Transition. *Dalton Trans.* **2019**, *48* (31), 11763–11771. DOI: 10.1039/c9dt02276g.

(3) Pfund, B.; Steffen, D. M.; Schreier, M. R.; Bertrams, M.-S.; Ye, C.; Börjesson, K.; Wenger, O. S.; Kerzig, C. UV Light Generation and Challenging Photoreactions Enabled by Upconversion in Water. *J. Am. Chem. Soc.* **2020**, *142* (23), 10468–10476. DOI: 10.1021/jacs.0c02835.

(4) Meech, S. R.; Phillips, D. Photophysics of Some Common Fluorescence Standards. *Journal of Photochemistry* **1983**, *23* (2), 193–217. DOI: 10.1016/0047-2670(83)80061-6.

(5) Montalti, M.; Credi, A.; Prodi, L.; Gandolfi, M. T. Handbook of Photochemistry 3rd Edition. *CRC Press, Taylor & Francis Group, Boca Raton, FL 33487-2742* **2006**.

(6) Lakowicz, J. R. *Principles of Fluorescence Spectroscopy*, 3rd ed.; Springer US: Boston, MA, 2006. DOI: 10.1007/978-0-387-46312-4.

(7) Ossinger, S.; Prescimone, A.; Häussinger, D.; Wenger, O. S. Manganese(I) Complex with Monodentate Arylisocyanide Ligands Shows Photodissociation Instead of Luminescence. *Inorg. Chem.* **2022**, *61* (27), 10533–10547. DOI: 10.1021/acs.inorgchem.2c01438.

(8) Rodriguez, T. M.; Deegbey, M.; Chen, C.-H.; Jakubikova, E.; Dempsey, J. L. Isocyanide Ligands Promote Ligand-to-Metal Charge Transfer Excited States in a Rhenium(II) Complex. *Inorg. Chem.* **2023**, *62* (17), 6576–6585. DOI: 10.1021/acs.inorgchem.2c03193.

(9) Herr, P.; Kerzig, C.; Larsen, C. B.; Häussinger, D.; Wenger, O. S. Manganese(I) Complexes with Metal-to-Ligand Charge Transfer Luminescence and Photoreactivity. *Nat. Chem.* **2021**, *13* (10), 956–962. DOI: 10.1038/s41557-021-00744-9.

(10) Hofbeck, T.; Yersin, H. The Triplet State of *Fac*-Ir(ppy)$_3$. *Inorg. Chem.* **2010**, *49* (20), 9290–9299. DOI: 10.1021/ic100872w.

(11) Büldt, L. A.; Guo, X.; Vogel, R.; Prescimone, A.; Wenger, O. S. A Tris(Diisocyanide)Chromium(0) Complex Is a Luminescent Analog of Fe(2,2’-Bipyridine)$_3^{2+}$. *J. Am. Chem. Soc.* **2017**, *139* (2), 985–992. DOI: 10.1021/jacs.6b11803.

(12) Caspar, J. V.; Kober, E. M.; Sullivan, B. P.; Meyer, T. J. Application of the Energy Gap Law to the Decay of Charge-Transfer Excited States. *J. Am. Chem. Soc.* **1982**, *104* (2), 630–632. DOI: 10.1021/ja00366a051.

(13) Bhasikuttan, A. C.; Suzuki, M.; Nakashima, S.; Okada, T. Ultrafast Fluorescence Detection in Tris(2,2’-Bipyridine)Ruthenium(II) Complex in Solution: Relaxation Dynamics Involving Higher Excited States. *J. Am. Chem. Soc.* **2002**, *124* (28), 8398–8405. DOI: 10.1021/ja026135h.

(14) Gawelda, W.; Cannizzo, A.; Pham, V.-T.; van Mourik, F.; Bressler, C.; Chergui, M. Ultrafast Nonadiabatic Dynamics of $[Fe^{II}(bpy)_3]^{2+}$ in Solution. *J. Am. Chem. Soc.* **2007**, *129* (26), 8199–8206. DOI: 10.1021/ja070454x.

(15) Kvapilová, H.; Sattler, W.; Sattler, A.; Sazanovich, I. V.; Clark, I. P.; Towrie, M.; Gray, H. B.; Záliš, S.; Vlček, A. Electronic Excited States of Tungsten(0) Arylisocyanides. *Inorg. Chem.* **2015**, *54* (17), 8518–8528. DOI: 10.1021/acs.inorgchem.5b01203.

(16) Liard, D. J.; Busby, M.; Matousek, P.; Towrie, M.; Vlček, A. Picosecond Relaxation of ^{3}MLCT Excited States of $[Re(Etpy)(CO)_3(Dmb)]^+$ and $[Re(Cl)(CO)_3(bpy)]$ as Revealed by Time-Resolved Resonance Raman, UV−vis, and IR Absorption Spectroscopy. *J. Phys. Chem. A* **2004**, *108* (13), 2363–2369. DOI: 10.1021/jp0366320.

(17) Damrauer, N. H.; Cerullo, G.; Yeh, A.; Boussie, T. R.; Shank, C. V.; McCusker, J. K. Femtosecond Dynamics of Excited-State Evolution in $[Fe(bpy)_3]^{2+}$. *Science* **1997**, *275* (5296), 54–57. DOI: 10.1126/science.275.5296.54.

(18) Patil, P.; Ahmadian-Moghaddam, M.; Dömling, A. Isocyanide 2.0. *Green Chem.* **2020**, *22* (20), 6902–6911. DOI: 10.1039/D0GC02722G.

(19) Mukhopadhyay, S.; Patro, A. G.; Vadavi, R. S.; Nembenna, S. Coordination Chemistry of Main Group Metals with Organic Isocyanides. *Eur. J. Inorg. Chem.* **2022**, *2022* (31). DOI: 10.1002/ejic.202200469.

(20) Frisch, M. J.; Trucks, G. W.; Schlegel, H. B.; Scuseria, G. E.; Robb, M. A.; Cheeseman, J. R.; Scalmani, G.; Barone, V.; Petersson, G. A.; Nakatsuji, H.; Li, X.; Caricato, M.; Marenich, A. V.; Bloino, J.; Janesko, B. G.; Gomperts, R.; Mennucci, B.; Hratchian, H. P.; Ortiz, J. V.; Izmaylov, A. F.; Sonnenberg, J. L.; Williams-Young, D.; Ding, F.; Lipparini, F.; Egidi, F.; Goings, J.; Peng, B.; Petrone, A.; Henderson, T.; Ranasinghe, D.; Zakrzewski, V. G.; Gao, J.; Rega, N.; Zheng, G.; Liang, W.; Hada, M.; Ehara, M.; Toyota, K.; Fukuda, R.; Hasegawa, J.; Ishida, M.; Nakajima, T.; Honda, Y.; Kitao, O.; Nakai, H.; Vreven, T.; Throssell, K.; Montgomery, Jr., J. A.; Peralta, J. E.; Ogliaro, F.; Bearpark, M. J.; Heyd, J. J.; Brothers, E. N.; Kudin, K. N.; Staroverov, V. N.; Keith, T. A.; Kobayashi, R.; Normand, J.; Raghavachari, K.; Rendell, A. P.; Burant, J. C.; Iyengar, S. S.; Tomasi, J.; Cossi, M.; Millam, J. M.; Klene, M.; Adamo, C.; Cammi, R.; Ochterski, J. W.; Martin, R. L.; Morokuma, K.; Farkas, O.; Foresman, J. B.; Fox, D. J. *Gaussian 16*; Gaussian, Inc., Wallingford CT, 2016.

(21) Herr, P.; Schwab, A.; Kupfer, S.; Wenger, O. S. Deep-Red Luminescent Molybdenum(0) Complexes with Bi- and Tridentate Isocyanide Chelate Ligands. *ChemPhotoChem* **2022**. DOI: 10.1002/cptc.202200052.

(22) Becke, A. D. Density-Functional Thermochemistry. III. The Role of Exact Exchange. *J. Chem. Phys.* **1993**, *98* (7), 5648. DOI: 10.1063/1.464913.

(23) Becke, A. D. Density-Functional Exchange-Energy Approximation with Correct Asymptotic Behavior. *Phys. Rev. A Gen. Phys.* **1988**, *38* (6), 3098–3100. DOI: 10.1103/physreva.38.3098.

(24) Lee, C.; Yang, W.; Parr, R. G. Development of the Colle-Salvetti Correlation-Energy Formula into a Functional of the Electron Density. *Phys Rev, B Condens Matter* **1988**, *37* (2), 785–789. DOI: 10.1103/PhysRevB.37.785.

(25) Weigend, F.; Ahlrichs, R. Balanced Basis Sets of Split Valence, Triple Zeta Valence and Quadruple Zeta Valence Quality for H to Rn: Design and Assessment of Accuracy. *Phys. Chem. Chem. Phys.* **2005**, *7* (18), 3297–3305. DOI: 10.1039/b508541a.

(26) Weigend, F. Accurate Coulomb-Fitting Basis Sets for H to Rn. *Phys. Chem. Chem. Phys.* **2006**, *8* (9), 1057–1065. DOI: 10.1039/b515623h.

(27) Grimme, S.; Ehrlich, S.; Goerigk, L. Effect of the Damping Function in Dispersion Corrected Density Functional Theory. *J. Comput. Chem.* **2011**, *32* (7), 1456–1465. DOI: 10.1002/jcc.21759.

(28) Marenich, A. V.; Cramer, C. J.; Truhlar, D. G. Universal Solvation Model Based on Solute Electron Density and on a Continuum Model of the Solvent Defined by the Bulk Dielectric Constant and Atomic Surface Tensions. *J. Phys. Chem. B* **2009**, *113* (18), 6378–6396. DOI: 10.1021/jp810292n.

(29) Mennucci, B.; Cappelli, C.; Guido, C. A.; Cammi, R.; Tomasi, J. Structures and Properties of Electronically Excited Chromophores in Solution from the Polarizable Continuum Model Coupled to the Time-Dependent Density Functional Theory. *J. Phys. Chem. A* **2009**, *113* (13), 3009–3020. DOI: 10.1021/jp8094853.

(30) Kupfer, S. Simulated Electronic Properties of [Cr], $[Mn]^{+}$ and $[Fe]^{2+}$. *Zenodo* **2023**. DOI: 10.5281/zenodo.8246834.

(31) Yu, H. S.; He, X.; Li, S. L.; Truhlar, D. G. MN15: A Kohn–Sham Global-Hybrid Exchange–Correlation Density Functional with Broad Accuracy for Multi-Reference and Single-Reference Systems and Noncovalent Interactions. *Chem. Sci.* **2016**, *7* (8), 5032–5051. DOI: 10.1039/C6SC00705H.

(32) Yanai, T.; Tew, D. P.; Handy, N. C. A New Hybrid Exchange–Correlation Functional Using the Coulomb-Attenuating Method (CAM-B3LYP). *Chem. Phys. Lett.* **2004**, *393* (1–3), 51–57. DOI: 10.1016/j.cplett.2004.06.011.

(33) Zedler, L.; Mengele, A. K.; Ziems, K. M.; Zhang, Y.; Wächtler, M.; Gräfe, S.; Pascher, T.; Rau, S.; Kupfer, S.; Dietzek, B. Unraveling the Light-Activated Reaction Mechanism in a Catalytically Competent Key Intermediate of a Multifunctional Molecular Catalyst for Artificial Photosynthesis. *Angew. Chem. Int. Ed* **2019**, *58* (37), 13140–13148. DOI: 10.1002/anie.201907247.

(34) Shillito, G. E.; Hall, T. B. J.; Preston, D.; Traber, P.; Wu, L.; Reynolds, K. E. A.; Horvath, R.; Sun, X. Z.; Lucas, N. T.; Crowley, J. D.; George, M. W.; Kupfer, S.; Gordon, K. C. Dramatic Alteration of ^{3}ILCT Lifetimes Using Ancillary Ligands in $[Re(L)(CO)_3(phen\text{-}TPA)]^{n+}$ Complexes: An Integrated Spectroscopic and Theoretical Study. *J. Am. Chem. Soc.* **2018**, *140* (13), 4534–4542. DOI: 10.1021/jacs.7b12868.

(35) Ziegler, T.; Rauk, A.; Baerends, E. J. On the Calculation of Multiplet Energies by the Hartree-Fock-Slater Method. *Theor. Chim. Acta* **1977**, *43* (3), 261–271. DOI: 10.1007/BF00551551.

(36) Merrick, J. P.; Moran, D.; Radom, L. An Evaluation of Harmonic Vibrational Frequency Scale Factors. *J. Phys. Chem. A* **2007**, *111* (45), 11683–11700. DOI: 10.1021/jp073974n.

(37) Johnson, R. D. Computational Chemistry Comparison and Benchmark Database, NIST Standard Reference Database 101. *National Institute of Standards and Technology* **2022**. DOI: 10.18434/t47c7z.
